# Pollen-mediated gene flow from transgenic perennial creeping bentgrass and hybridization at the landscape level

**DOI:** 10.1371/journal.pone.0173308

**Published:** 2017-03-03

**Authors:** María Luz Zapiola, Carol Ann Mallory-Smith

**Affiliations:** Department of Crop and Soil Science, Oregon State University, Corvallis, Oregon, United States of America; Washington University, UNITED STATES

## Abstract

The planting of 162 ha of transgenic glyphosate-resistant creeping bentgrass (*Agrostis stolonifera*) near Madras, OR, USA, allowed a unique opportunity to study gene flow over time from a perennial outcrossing species at the landscape level. While conducting a four year *in situ* survey, we collected panicles and leaf tissue samples from creeping bentgrass and its sexually compatible species. Seeds from the panicles were planted, and seedlings were tested in the greenhouse for expression of the transgene. Gene flow via pollen was found in all four years, at frequencies of 0.004 to 2.805%. Chloroplast markers, in combination with internal transcribed spacer nuclear sequence analysis, were used to aid in identification of transgenic interspecific and intergeneric hybrid seedlings found during the testing and of established plants that could not be positively identified in the field. Interspecific transgenic hybrids produced on redtop (*Agrostis gigantea*) plants *in situ* were identified three of the four years and one intergeneric transgenic creeping bentgrass x rabbitfoot grass (*Polypogon monspeliensis*) hybrid was identified in 2005. In addition, we confirmed a non-transgenic creeping bentgrass x redtop hybrid *in situ*, demonstrating that interspecific hybrids have established in the environment outside production fields. Results of this study should be considered for deregulation of transgenic events, studies of population dynamics, and prediction of gene flow in the environment.

## Introduction

Gene flow, defined as the change in gene frequency due to movement of gametes, individuals or groups of individuals from one place to another [1], has a role in plant evolution [2]. Gene flow is a naturally occurring phenomenon that can occur between plants of the same species or between plants of sexually compatible species, producing interspecific and occasionally intergeneric hybrids [3, 4]. Gene flow from transgenic crops to feral populations, compatible crops, naturalized or wild sexually compatible species, is one of the main concerns which should be considered before the deregulation of a new genetically engineered event [5, 6, 7, 8]. When analyzing the risks of gene flow from transgenic crops, it is important to determine how the transgene will spread, if the transgenic crop will hybridize with compatible relatives, and how critical a problem, if any, the transgene would be when it escapes [9].

One of the challenges of gene flow studies is that they are performed either in greenhouses or at reduced scales, which are too small to capture the extent of gene movement [10]. Therefore, the evaluation of gene flow under controlled conditions is not always applicable to situations at the landscape level. Gene flow studies at the landscape level are a key component for understanding the probability of gene flow in the environment and its potential impact. Transgenic crops provide a useful system to study gene flow because the presence of the transgene is easy to track. The study of such systems also contributes to understanding evolutionary dynamics. In addition, information about gene flow at the landscape level contributes to a better understanding of gene flow from weed populations that evolve herbicide resistance and of implications for resistance prevention and management.

Creeping bentgrass (*Agrostis stolonifera* L.) is a perennial, outcrossing, wind pollinated, tetraploid, cosmopolitan, small seeded species that propagates through seeds and stolons [11]. Pollen viability in creeping bentgrass was reported to be 1% at 2 h after release [12, 13]. Creeping bentgrass can establish feral populations outside of cultivation and form a complex with other sexually compatible *Agrostis* spp., including the rhizomonous perennial redtop (*A*. *gigantea* Roth), which are often sympatric in areas where creeping bentgrass seed is produced. The species in the complex produce interspecific hybrids with varying degrees of pollen fertility and seed set [14, 15, 16]. Creeping bentgrass also can hybridize with rabbitfoot grass (*Polypogon monspeliensis* (L.) Desfontaines) [14] resulting in the intergeneric hybrid perennial beard-grass (x *Agropogon littoralis* (Sm.) Hubbard) [17]. The fact that *Agrostis* species are extremely morphologically variable combined with interspecific and intergeneric hybridization, contribute to the difficulty of species identification [18].

Transgenic glyphosate resistant creeping bentgrass (GRCB) was developed by The Scotts Company and Monsanto Company. In 2002, while still a regulated event, 162 ha of GRCB were planted for seed production within a 4,500 ha seed production control area established by the Oregon Department of Agriculture (ODA) north of Madras, Oregon, USA. Seed production practices within the control area, including isolation distances, were regulated in an attempt to prevent transgene flow to compatible species [19]. Feral creeping bentgrass and at least two compatible species, redtop and rabbitfoot grass, were present in the region where the GRCB seed production control area was established. The transgenic GRCB fields produced pollen and seed for one year and strong winds, characteristic of the area, moved swathed panicles filled with ripe seeds off the production fields in 2003. The GRCB crops were terminated after harvest in summer 2003. A mitigation program was put in place by The Scotts Company to remove escaped transgenic plants established *in situ*.

The introduction of transgenic GRCB into the environment, on such a large scale, presented a unique opportunity to study gene flow over time from an outcrossing perennial species. From 2003 through 2006, we conducted an *in situ* survey within and up to 5 km outside the GRCB seed production control area to determine the number and proportion of transgenic plants established outside of cultivation. Results of the survey were presented in [20]. As a complement to the survey, we conducted this four-year project to assess the occurrence of pollen-mediated gene flow at the landscape level and confirm the introgression of the transgene in feral populations of creeping bentgrass and related species.

## Materials and methods

### Plant material

Panicles and tissue samples from creeping bentgrass, redtop, rabbitfoot grass, and other *Agrostis* spp. identified during the 2003–2006 *in situ* survey [20] were collected in paper envelopes. The area surveyed totaled 610 km of irrigation canals, ditch and pond banks, roadsides and pipelines, within and up to 5 km outside the GRCB seed production control area established by the ODA, north of Madras (44° 38’ 1” N, 121° 7’ 42” W), Oregon, USA [20]. The survey was conducted mainly on public access areas and along the North Unit Irrigation District’s canals and ditches, where we had permission to survey. When we entered private property, we did so at the owner’s request or with permission. The surveyed area did not involve endangered or protected species. Panicles collected from 437 glyphosate-susceptible and 80 glyphosate-resistant plants were hand-threshed and an identification number was given by plant.

### Herbicide screening

**Fig 1:**
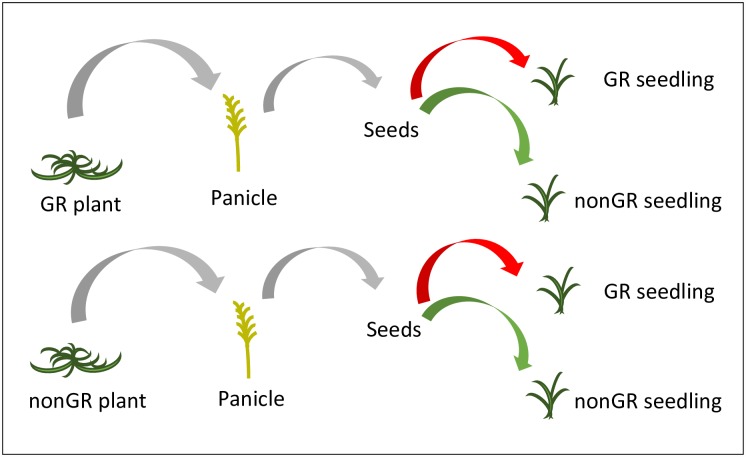
Schematics of seed collection and herbicide screening. Panicles collected from transgenic glyphosate-resistant (GR) and glyphosate-susceptible (non GR) plants established *in situ* were threshed and the seeds were screened for the presence of the glyphosate resistance transgene.

Glyphosate resistance was screened at three levels: phenotype, protein expression, and molecular. Seeds, originating from glyphosate-susceptible and glyphosate-resistant plants were planted in nine rows in 27.5 x 52.5 x 5 cm trays filled with potting mix (Sunshine Mix #1/LC, Sun Gro, Bellevue, WA) and grown in the greenhouse for glyphosate screening (Fig 1). Two weeks after planting, when most seedlings were at the two-leaf stage, seedlings were counted and sprayed with glyphosate at 1.68 kg a.e. ha^-1^. Fourteen days after application, surviving seedlings were counted and resprayed with the same rate of glyphosate. Twenty-one days after the second application, surviving seedlings were counted and tested with Trait✓^®^ RUR lateral flow strips (Strategic Diagnostics, Newark, DE, USA) to confirm the expression of the glyphosate-resistant 5-enolpyruvyl-shikimate-3-phosphate synthase protein from *Agrobacterium* sp. strain CP4 (CP4 EPSPS). Finally, the presence of the *cp4 epsps* transgene was confirmed using molecular analysis.

### Molecular analysis

Molecular markers were used to test for transgene presence and for hybrid confirmation. Total genomic DNA was extracted, using the DNeasy 96 Plant kit (Qiagen, Valencia, CA) and DNeasy Plant Mini Kit (Qiagen), from fresh young leaves of seedlings that survived the herbicide screening in the greenhouse and from dried young leaves of plants tested *in situ*.

#### *cp4* *epsps*

The presence of the glyphosate resistance transgene was confirmed by PCR. We designed and used primers CP4F ACGATTTCGACAGCACCTTC and CP4R TGCAGCATCTTTTCCGTATG. The PCR mixture (15 μL) contained 2–5 ng of genomic DNA, 1x CoralLoad buffer, 1 x Q-solution, 200 μM of each dNTP, 0.5 μM of each primer, and 0.5 U reaction^-1^ *Taq* DNA Polymerase (Qiagen). The PCR program consisted of: 3 min at 94°C, followed by 30 cycles of 30 s at 94°C, 20 s at 55°C, and 30 s at 72°C, with a final extension of 10 min at 72°C, using a C1000TM Thermal Cycler (Bio-Rad, Hercules, CA). Presence of PCR amplicons (286 bp) was confirmed by UV fluorescence after electrophoresis on 2% agarose gels stained with ethidium bromide.

#### Species identification

Definitive species identification of some samples based solely on morphological characteristics was difficult, and even more complicated because of the potential for interspecific and intergeneric hybridization. Therefore, we used chloroplast microsatellites (cpSSR) markers and a *mat*K insertion-deletion (indel) marker, in combination with nuclear ribosomal DNA internal transcribed spacer (ITS) sequences, to aid in identification of 13 seedlings that survived the herbicide screening and 11 plants found *in situ*.

cpSSR. The *Agrostis* chloroplast microsatellites (cpSSRs) markers Acp12, Acp18, Acp19, Acp22, Acp23, Acp24, Acp26b, Acp27b, Acp28b, Acp30b, Acp31, and Acp32 developed by [21] were used to identify the chloroplast type. The PCR reaction mixture (15 μL) contained 2–5 ng of genomic DNA, 1 x Phusion HF buffer, 200 μM each dNTP, 0.4 μM each primer, and 0.02 U μL^-1^ Phusion^™^ High-Fidelity DNA Polymerase (Finnzymes). The PCR program consisted of: 2 min at 98°C, followed by 30 cycles of 10 s at 98°C, 20 s at 60–63°C (primer-specific), and 20 s at 72°C, with a final extension of 10 min at 72°C, using a C1000TM Thermal Cycler (Bio-Rad). Uniformity of PCR amplification products was confirmed by UV fluorescence after electrophoresis on 2% agarose gel with ethidium bromide. PCR products were multiplexed in two sets (1:400–1:1000 dilution) and were screened by capillary electrophoresis using an ABI Prism^®^ 3100 Genetic Analyzer. Genotypes were scored manually using ABI Prism^®^ Genotyper version 3.7 NT (Applied Biosystems).

*matK* indel. Based on alignments of *mat*K sequences (GeneBank accessions DQ146797-DQ146826) from velvet bentgrass (*A*. *canina L*.), colonial bentgrass (*A*. *capillaris* L.), dryland bentgrass (*A*. *castellana* Boiss. and Reuter), spike bentgrass (*A*. *exarata* Trin.), redtop, Idaho bentgrass (*A*. *idahoensis* Nash), northern bentgrass (*A*. *mertensii* Trin.), seashore bentgrass (*A*. *pallens* Trin.), rough bentgrass (*A*. *scabra* Willd.), creeping bentgrass, brown bentgrass (*A*. *vinealis* Schreber), rabbitfoot grass, and water bent (*P*. *viridis* (Gouan) Breistr.), a pair of primers was designed to amplify a segment that contains a 18bp duplication for some, but not all of the species. Primers mkD1F CACGACTGATCCTCAAGGGTA and mkD1R TTCACTAGACCCAGAAAATCGT were designed using Primer3 [22]. The PCR mixture (15 μL) contained 2–5 ng of genomic DNA, 1x CoralLoad buffer, 200 μM each dNTP, 0.5 μM each primer, and 0.5 U reaction^-1^ *Taq* DNA Polymerase (Qiagen). The PCR program consisted of: 2 min at 94°C, followed by 30 cycles of 20 s at 94°C, 20 s at 56°C, and 35 s at 72°C, with a final extension of 10 min at 72°C, using a C1000TM Thermal Cycler (Bio-Rad). PCR amplification products (106 and 124 bp) were visualized by UV fluorescence after electrophoresis on 4% agarose gels stained with ethidium bromide.

ITS. Primers used to amplify the nuclear ribosomal DNA internal transcribed spacer ITS1-5.8S-ITS2 (ITS) sequence were ITS5 GGAAGTAAAAGTCGTAACAAGG [23] and P216R CGTCGTGCGCACCGTTCAWAGGG [24]. PCR mixture of 25 μL was used following a modification of the [24] protocol, using 30 s at 56°C for annealing. PCR amplicons were cloned with TOPO TA Cloning Kit for Sequencing (Invitrogen, Carlsbad, CA). Eight to 10 clones per plant were screened by PCR. PCR products were purified using QIAquick PCR Purification Kits (Qiagen), and sequenced at the Center for Genome Research and Biocomputing Core Laboratories at Oregon State University on an ABI Prism^®^ 3100 Genetic Analyzer (Applied Biosystems, Carlsbad, CA).

#### Analysis

Plants were separated into two groups based on their matK indel marker amplification size (124 for creeping bentgrass, and 106 for redtop and rabbitfoot grass) and cpSSR data were analyzed in Structurama [25] using a model with expected number of populations as a random variable and Markov chain Monte Carlo of 10,000 cycles to determine the number of subpopulations (K). A set of 23 additional creeping bentgrass, 16 redtop, and 12 rabbitfoot grass plants was used as a reference.

Nuclear ITS sequences were aligned in S CLC Sequence Viewer 5.0 (www.clcbio.com) for each plant and a consensus sequence was generated. When two distinct alleles were observed, implying hybridization, a consensus sequence for each allele was generated. The UPGMA tree was created in S CLC Sequence Viewer 5.0 using the ITS sequences (GeneBank accessions DQ146766-DQ146796) from velvet bentgrass, colonial bentgrass, dryland bentgrass, spike bentgrass, redtop, Idaho bentgrass, northern bentgrass, creeping bentgrass, rough bentgrass, and rabbitfoot grass, as references.

Species, interspecific, and intergeneric hybrids produced or established *in situ*, were confirmed based on placement of the plants in the chloroplast type group versus the nuclear ITS tree in combination with plant morphology.

## Results

**Fig 2:**
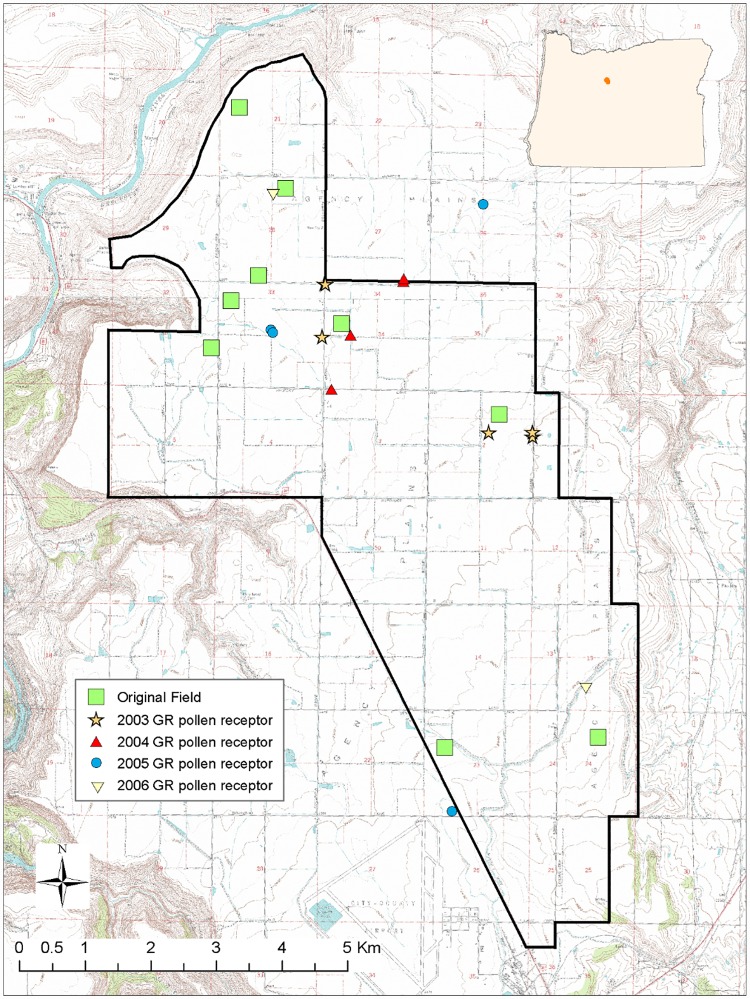
Location of susceptible plants that produced transgenic Glyphosate-Resistant (GR) seedlings (GR pollen receptor) in the four years. The black line is the limit of the transgenic glyphosate resistant creeping bentgrass (GRCB) seed production control area. Green squares represent the location of the original GRCB fields established in 2002 and removed after seed harvest in 2003. Stars, triangles, circles and inverted triangles represent the location of GR pollen receptor plants for 2003, 2004, 2005 and 2006, respectively. A single point may represent more than one plant. The inserted map shows the location of the control area in relation to the state of Oregon, USA.

Evidence of gene flow via pollen was found in all four years. Although we did not find any glyphosate-resistant (GR) seedlings originating from rabbitfoot grass plants, we did find GR seedlings originating from redtop and creeping bentgrass plants in three of the four years and GR seedlings originating from other *Agrostis* spp. or potential hybrids with creeping bentgrass in 2004 and 2006 (Table 1; Fig 2).

**Table 1:** Glyphosate screening of seedlings from susceptible established plants. Number of seedlings sprayed (Seedlings) and glyphosate-resistant (R) seedlings, of seed collected *in situ* from susceptible plants of each species from 2003 through 2006.

Species	2003		2004		2005		2006	
	Seedlings	R	Seedlings	R	Seedlings	R	Seedlings	R
Rabbitfoot grass	528	0	62,999	0	13,847	0	45,852	0
Redtop	14,351	29	138,960	51	21,585	0	51,277	2
Creeping bentgrass	1,063	29	719	9	6,394	16	3,494	0
*Agrostis* spp.	21	0	4,293	6	2,431	0	4,209	4
Total	15,963	58	206,971	66	44,257	16	104,832	6

For seeds collected from susceptible redtop plants, the percentages of GR seedlings were 0.202, 0.037, 0, and 0.004% for 2003, 2004, 2005, and 2006, respectively (Table 1). For seeds collected from susceptible creeping bentgrass plants, the percentages of GR seedlings were 2.73, 1.25, 0.25, and 0% for 2003, 2004, 2005, and 2006, respectively. The percentage of GR seedlings originating from susceptible plants that were identified only to *Agrostis* spp. were 0, 0.14, 0, and 0.095% for 2003, 2004, 2005, and 2006, respectively.

The number of susceptible plants from which panicles were collected during the four years was 39 rabbitfoot grass, 263 redtop, 89 creeping bentgrass and 46 *Agrostis* spp. (Table 2). The percentages of susceptible redtop plants sampled *in situ* that produced GR seedlings were 23.53,1.39, and 1.87% for 2003, 2004, and 2006, respectively. For creeping bentgrass the percentages of susceptible plants that produced GR seedlings were 75, 60, and 7.55% for 2003, 2004, and 2005, respectively. For *Agrostis* spp. 20 and 6.67% of the susceptible plants from which panicles were collected produced GR seedlings in 2004 and 2006, respectively. Because the area sampled was not the same for each year [20], direct comparison of the results from different years was not possible.

**Table 2:** Percentage of susceptible established plants that produced at least one transgenic seedling. Number of susceptible plants that produced transgenic glyphosate-resistant seedlings (R) of the total number of glyphosate-susceptible plants (Plants) of each species from which panicles were collected and seed tested for 2003 through 2006. The % R is the percentage of susceptible plants sampled that produced glyphosate-resistant seedlings.

Species	2003			2004			2005			2006		
	Plants	R	% R	Plants	R	% R	Plants	R	% R	Plants	R	% R
Rabbitfoot grass	1	0	0	11	0	0	5	0	0	22	0	0
Redtop	17	4	23.53	72	1	1.39	67	0	0	107	2	1.87
Creeping bentgrass	4	3	75.00	5	3	60.00	53	4	7.55	27	0	0
*Agrostis* spp.	3	0	0	10	2	20.00	18	0	0	15	1	6.67
Total	25	7	28.00	98	6	6.12	143	4	2.80	171	3	1.75

The percentage of resistant seedlings from seed originated from established GRCB plants was 99% of 451, 90% of 3,591, and 94% of 484 seedlings screened for 2004, 2005, and 2006, respectively. The number of GRCB plants that produced GR seedlings was 10 of 10, 53 of 56, and 11 of 14 plants from which panicles were collected for 2004, 2005, and 2006, respectively. In 2003, as we expected, no GRCB plants were found established outside of the GRCB fields [20]; therefore, no panicles were collected from GRCB plants that year.

**Fig 3:**
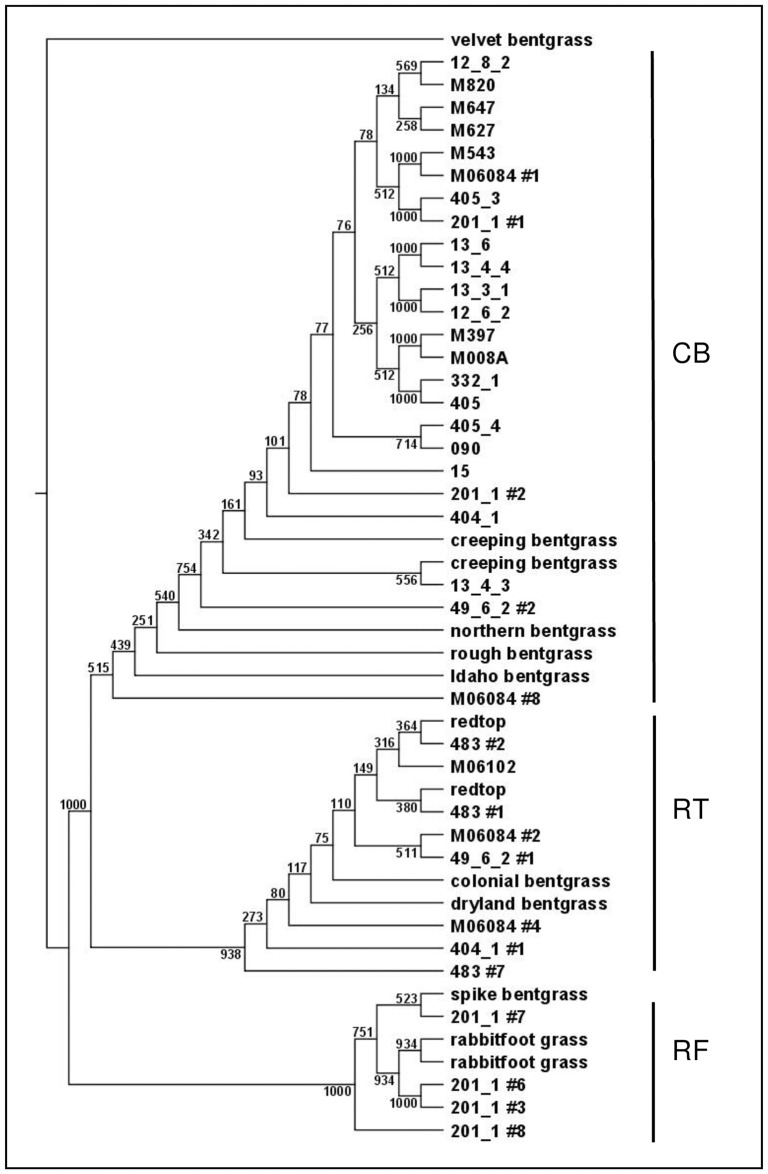
Bootstrapped UPGMA tree for ITS sequences. The number following # is the clone number presented when sequences were different. The number following the last underscore implies the plant survived the greenhouse screening. No underscore means the mother plant, except for 15 that was a single seedling (15_1). Numbers on branches are Bootstrap values.

Chloroplast data (*matK* indel and cpSSRs), combined with the presence of the GR transgene, confirmed 82 transgenic seedlings from seed produced *in situ* on susceptible redtop plants (S1 Table). Two transgenic GR redtop x creeping bentgrass hybrid seedlings were further characterized using ITS nuclear sequences (Table 3; Fig 3; S1 Appendix). Of the 52 transgenic seedlings produced *in situ* on susceptible creeping bentgrass and putative creeping bentgrass plants, four creeping bentgrass x GRCB seedlings were characterized. We were unable to confirm as hybrids six seedlings that were suspected to be hybrids based on morphology, as they were GR and had rhizomes, but they had a creeping bentgrass chloroplast haplotype and only creeping bentgrass sequences for the ITS clones that we sequenced (S1 Table; S1 Appendix). The seedlings could have been a result of a creeping bentgrass x redtop susceptible hybrid pollinated by GR creeping bentgrass, or a susceptible creeping bentgrass pollinated by a GR hybrid. The ploidy levels, creeping bentgrass (4x) and redtop and rabbitfoot grass (6x), combined with the unpredictable crossing result in many possible allele combinations. In addition, the unknown background of the plants established and the potential that some were backcrosses made it extremely difficult to determine, in many cases, the direction and level of crossing. From the testing of seedlings originating from GRCB plants *in situ*, an off type seedling was further studied and confirmed as a GRCB x rabbitfoot grass intergeneric hybrid [26].

**Table 3:** Characteristics of seedlings screened. Seedling ID (Plant ID), year of identification (Year), type of mother plant (Mpl), glyphosate-resistance trait of mother plant (GRMpl), presence of rhizomes and stolons in the seedling, glyphosate-resistance trait of seedling (GR), hypothesized seedling type (Seedling), *matK* indel allele (matK), chloroplast haplotype (cpSSR), ITS sequence type (ITS), and species/hybrid call based on all information (Call).

Plant ID	Year	Mpl	GRMpl	Rhizomes	Stolons	GR	Seedling	matK	cpSSR	ITS	Call
49–6_2	2003	RT	no	yes	yes	yes	RT x CB	106	RT	RT, CB	RT x GR CB
404_1	2006	RT	no	yes	no	yes	RT x CB	106	RT	RT, CB	RT x GR CB
15_1	2003	CB	no	no	yes	yes	CB x CB	124	CB	CB	CB x GR CB
12–6_2	2004	put_CB	no	no	yes	yes	put_CB x CB	124	CB	CB	CB x GR CB
12–8_2	2004	put_CB	no	no	yes	yes	put_CB x CB	124	CB	CB	CB x GR CB
13–3_1	2004	put_CB	no	no	yes	yes	put_CB x CB	124	CB	CB	CB x GR CB
13–4_3	2004	put_Hyb	no	weak	yes	yes	put_Hyb x CB	124	CB	CB	Agrostis x GR Agrostis
13–4_4	2004	put_Hyb	no	yes	yes	yes	put_Hyb x CB	124	CB	CB	Agrostis x GR Agrostis
13–6_1	2004	put_Hyb	no	yes	yes	yes	put_Hyb x CB	124	CB	CB	Agrostis x GR Agrostis
405_3	2006	put_Hyb	no	yes	yes	yes	put_Hyb x CB	124	CB	CB	Agrostis x GR Agrostis
405_4	2006	put_Hyb	no	weak	yes	yes	put_Hyb x CB	124	CB	CB	Agrostis x GR Agrostis
332_1	2005	CB	no	yes	yes	yes	CB x Hyb	124	CB	CB	Agrostis x GR Agrostis
201_1	2005	CB	yes	no	yes	yes	CB x RF	124	CB	CB, RF	GR CB x RF

RT = redtop, CB = creeping bentgrass, RF = rabbitfoot grass, put_CB = putative creeping bentgrass, put_ Hyb = putative hybrid, Agrostis = *Agrostis* spp. unable to confirm species or hybrid, GR = transgenic glyphosate-resistant.

Using the DNA from tissue samples collected from established plants, we further characterized seven putative hybrids and one putative creeping bentgrass found *in situ* and compared them to a typical creeping bentgrass and two redtop plants. The use of *matK* indel, cpSSR markers and ITS sequences in combination with morphology, mainly the presence/absence of stolons and rhizomes, were used to identify three of the plants (Table 4; Fig 3; S1 Table; S1 Appendix). A putative interspecific, non-transgenic, hybrid between creeping bentgrass and redtop found *in situ* was confirmed, demonstrating that there are interspecific hybrids established *in situ*. However, there were four putative hybrids where the data were not enough to conclude whether they were hybrids (Table 4). We ran the nuclear SSR markers we previously developed [26] on this set of plants, but even though they contributed to identifying rabbitfoot grass from creeping bentgrass and redtop, they did not separate redtop from creeping bentgrass. Greater power to distinguish the nuclear composition is needed as some plants could be backcrosses of these polyploid species.

**Table 4:** Characteristics of plants established *in situ*. Plant ID (PlantID), year of identification (Year), type of plant based on phenotype (Type), glyphosate-resistance trait (GR), *matK* indel allele (matK), chloroplast haplotype (cpSSR), ITS sequence type (ITS), species/hybrid call based on all information (Call), and comments.

Plant ID	Year	Type	GR	matK	cpSSR	ITS	Call	Comments
483	2006	RT	no	106	RT	RT	RT	produced GR seedlings
M06102	2006	RT	no	n/a	n/a	RT	RT	no GR seedlings
M397	2005	CB	no	124	CB	CB	CB	produced GR seedlings
090	2005	put_CB	yes	124	CB	CB	GR CB	produced GR seedlings
M008A	2005	put_Hyb	no	124	CB	CB	CB	produced GR seedlings
M543	2006	put_Hyb	yes	124	CB	CB	GR CB	no seedlings tested
M06084	2006	put_Hyb	no	124	CB	CB, RT	CB x RT	no seedlings tested
405	2006	put_Hyb	no	124	CB	CB	Agrostis	produced GR seedlings
M627	2006	put_Hyb	yes	124	CB	CB	GR Agrostis	no seedlings tested
M647	2006	put_Hyb	yes	124	CB	CB	GR Agrostis	no seedlings tested
M820	2006	put_Hyb	yes	124	CB	CB	GR Agrostis	no seedlings tested

RT = redtop, CB = creeping bentgrass, put_CB = putative creeping bentgrass, put_Hyb = putative hybrid, Agrostis = *Agrostis* spp. unable to confirm species or hybrid, GR = transgenic glyphosate-resistant, n/a = not available.

## Discussion

Pollen-mediated gene flow is very dependent on a species’ characteristics. Creeping bentgrass has a particularly large potential for pollen-mediated gene flow because it is an outcrossing, wind pollinated, perennial that establishes feral populations and has numerous compatible relatives, with which it forms hybrids.

Other main factors that affect pollen-mediated gene flow are pollen source size, distance from source, pollen receptor size, and environmental conditions. Because the GRCB seed production fields were removed after harvest in 2003, the density of GR plants, and therefore the size of the pollen source, were clearly different in 2003 compared with the other years. In addition, GR plants were being removed as part of the mitigation program conducted by The Scotts Company. However, having susceptible redtop, creeping bentgrass, and putative hybrid plants that produced GR seedlings every year means that, even after the GRCB fields were taken out of production, and despite the ongoing mitigation program, there was enough GR pollen produced to allow for the persistence, introgression and further dissemination of the transgene in the environment.

Our results confirm the introgression of the glyphosate-resistance transgene in resident *Agrostis* spp. populations. Feral GR plants established along irrigation canals, ditches, pipes and any other water bodies in the area were a continuous source of pollen that allowed pollen-mediated gene flow to occur over the four years. Although not part of the original survey, we visited the area and tested for established transgenic *Agrostis* spp. plants at least once every year from 2008 through 2016 and identified GR plants *in situ* every year. These results are a consequence of a single introduction of GRCB at a large scale. However, if GRCB was commonly and extensively planted the frequency of transgenic pollen would increase significantly, especially when paired with glyphosate as a selection pressure.

The 2.8 and 0.2% pollen-mediated gene flow we found in 2003 is orders of magnitude greater than the 0.03 and 0.04% hybridization for resident creeping bentgrass and redtop, respectively, reported in 2003 by [27]. This difference could be due to the fact that they studied plants solely outside the control area, while we collected panicles from plants located closer to the pollen source, the GRCB production fields [20], representing different distances from the original pollen source.

There is a large difference between the low percentage of gene flow via pollen found in the herbicide screening of seedlings originating from susceptible creeping bentgrass mother plants (Table 1) and the 62% of established GRCB plants we found in 2006 during the *in situ* survey [20]. The level of pollen-mediated gene flow to susceptible creeping bentgrass plants reported here cannot account for all the GR plants found *in situ*. There are other means of gene flow, mainly seeds, that played a role in perpetuating the transgene in the environment. In fact, between 90 and 99% of the seeds produced on established GRCB plants from 2004 to 2006 were transgenic, demonstrating the great potential of established plants to contribute GR seeds into the environment. Seed-mediated gene flow differs from pollen-mediated gene flow in that there is no need for sympatric pollen donor and pollen receptor plants with overlapping flowering periods. In seed-mediated gene flow, the gene could be contributed by either a self-pollinated or cross-pollinated mother plant. Seed-mediated gene flow could result in gene movement in both space and time because seeds remain viable longer [28] than pollen grains. The lack of availability of the original transgenic seed planted or produced in the area greatly limited our ability to use the cpSSR markers and nuclear ITS sequence to identify the degree of relationship of tested plants to the original transgenic cultivar and prevented the determination of gene flow via seed versus pollen.

These findings show the potential for gene flow via pollen, but also question the feasibility of male sterility to contain a transgene [29], because of other means that allow a transgene to move. In fact, for the GR intergeneric hybrid produced *in situ*, the transgene source was the GRCB mother plant, not the rabbitfoot grass, which was the pollen donor [26]. The same situation applies for the high proportion of GR seedlings originating from GR plants established *in situ*, where the means of gene flow would be mainly seeds or potentially vegetative propagules.

Regardless on how the gene or transgene moves, the frequency of a gene or transgene in a population and the fate of a trait in the environment depend on competitive advantage, selection pressure, and life cycle of the species. Glyphosate-resistant creeping bentgrass plants do not have a competitive advantage over non-transgenic plants unless glyphosate is applied [30, 31], nor do they have a fitness cost in the absence of glyphosate [30, 31]. In this particular case, the selection pressure of glyphosate applications by growers and the irrigation district staff, combined with the fact that the perennial plants thrive and produce seeds for several years, would have contributed to the elevated frequency of transgenic plants found *in situ* when compared with the frequencies of gene flow through pollen.

The GR seedlings originating from susceptible redtop plants, based on cpSSR and *mat*K indel markers, emphasize the potential of creeping bentgrass to produce interspecific hybrids with sympatric compatible species. The hybrid seedlings were vigorous and long-lived, and had both stolons and rhizomes with varying degrees of development. The confirmation of an interspecific creeping bentgrass x redtop hybrid established *in situ*, that could not be confirmed based solely on morphological characteristics, demonstrated that there were interspecific hybrids already established that could receive and in future generations produce transgenic pollen.

Not only did we confirm 82 transgenic interspecific hybrids produced *in situ* on susceptible redtop plants, but also an intergeneric GRCB x rabbitfoot grass hybrid (perennial beard-grass) produced on a resident GRCB plant as previously described [26]. The GR interspecific and intergeneric hybrids produced *in situ* confirm the complexity of the system and highlight the difficulties for mitigation practices because plants with intermediate characteristics may not be identified and destroyed. Because of the long history of coexistence of creeping bentgrass, redtop and rabbitfoot grass *in situ*, the limited nature of our survey, and the potential for misidentification, we are confident that there were other interspecific, and potentially intergeneric, hybrids established *in situ* that we did not find or identify during the survey.

## Conclusions

This research confirmed the occurrence of pollen-mediated gene flow between creeping bentgrass and related species at the landscape level over time, and contributes to the understanding of the fate of the *cp4 epsps* transgene. Thirteen years after the removal of the GRCB production fields, the glyphosate-resistance transgene persists in the environment. This study demonstrates that analyzing gene flow through pollen alone, even at a landscape level, is not a good prediction of the fate of a gene or transgene in the environment. The potential for gene flow through pollen, seeds and vegetative propagules, as well as the competitive advantage of the trait without and, more importantly, with selection pressure, and finally the life cycle of the species, should be considered to better understand and predict the fate of a gene or transgene in the environment. Results of this study should be considered when deregulating transgenic outcrossing, wind-pollinated, perennial crops, and to analyze the potential for coexistence of transgenic and non-transgenic outcrossing grass seed crops. Despite the low frequencies of pollen-mediated gene flow we report and the ongoing mitigation program, 62% of the creeping bentgrass plants tested *in situ* during the survey in 2006 were transgenic. These results emphasize that gene flow studies, even at the landscape level, need to consider more than just pollen movement. Gene flow through pollen, seeds, and potentially vegetative propagules, along with the fact that creeping bentgrass is a perennial promiscuous outcrossing species, combined with the selection pressure of glyphosate being used often on the waterways, contributed to the continuous presence of the transgene in the environment.

## Supporting information

S1 Appendix ITS sequence data. (PDF)

S1 Table Chloroplast data. Plant and seedling ID (Sample Name), presence of the *cp4 epsps* transgene (GR), allele for the *matK* indel marker (matKdel), alleles for each of the 12 *Agrostis* chloroplast SSR markers (Acp_), and chloroplast haplotype (cpSSR).
(PDF)
