# Supplementary material for: Pollen-mediated gene flow from transgenic perennial creeping bentgrass and hybridization at the landscape level
Source: PLoS One. 2017 Mar 3;12(3):e0173308. doi: 10.1371/journal.pone.0173308 (PMC5336273; doi:10.1371/journal.pone.0173308)
Supplement: S1 Appendix — (PDF) [file pone.0173308.s001.pdf]

>ITS.332_1-C1 7638_G04_010-G05_018rc.complete
GGAAGTAAAAGTCGTAACAAGGTTTCCGTAGGTGAACCTGCGGAAGGATCATTGTCGTGA
CCCTGACCAAAATAGACCGTGCACGCGTCATCCAGCCTGCCGGCGGCGGCACCGTTCGTC
GCTCGGCCAAGTCCTCGACAACCTCCTCTCCTCGGAGTGGGGGCTCGGGGTAAAAGAACC
CACGACGCCTAAGGCGTCAAGGAACACTGTGCCTAGCCCGGGGACGCGGACGGCTTGCTG
GCCGCCCCCCGTGCTGCAATGCTATTTAATCCACACGACTCTCGGCAACGGATATCTCGG
CTCTCGCATCGATGAAGAACGTAGCGAAATGCGATACCTGGTGTGAATTGCAGAATCCCG
CGAACCATCGAGTCTTTGAACGCAAGTTGCGCCCGAGGCCATTCGGCTGAGGGCACGCCT
GCCTGGGCGTCACGCAAAACACGCTCCCACACCCTCATCGGGTAGCGGGACGCGGCATGT
GGTCCCCCGTAGCGCAAGCTGCGGTGGGCCGAAGATATGGCTGCCGGCGTATCGTGCCGG
ACACAGCGCGTGGTGGGCGACCTCGCTTTACTTACCGCAGTGCATCCGGGGCGTAGCCGA
CGTGATGGCCTCTAAGGACCCTATGAACGGTGCGCACGACG
>ITS.332_1-C2 7638_H04_009-H05_017rc.complete
GGAAGTAAAAGTCGTAACAAGGTTTCCGTAGGTGAACCTGCGGAAGGATCATTGTCGTGA
CCCTGACCAAAATAGACCGTGCACGCGTCATCCAGCCTGCCGGCGGCGGCACCGTTCGTC
GCTCGGCCAAGTCCTCGACAACCTCCTCTCCTCGGAGTGGGGGCTCGGGGTAAAAGAACC
CACGACGCCTAAGGCGTCAAGGAACACTGTGCCTAGCTCGGGGACGCGGACGGCTTGCTG
GCCGCCCCTCGTGCTGCAATGCTATTTAATCCACACGACTCTCGGCAACGGATATCTCGG
CTCTCGCATCGATGAAGAACGTAGCGAAATGCGATACCTGGTGTGAATTGCAGAATCCCG
CGAACCATCGAGTCTTTGAACGCAAGTTGCGCCCGAGGCCATTCGGCTGAGGGCACGCCT
GCCTGGGCGTCACGCAAAACACGCTCCCACACCCTCATTGGGTAGCGGGACGCGGCATGT
GGTCCCCCGTAGCGCAAGCTGCGGTGGGCCGAAGATATGGCTGCCGGCGTATCGTGCCGG
ACACAGCGCGTGGTGGGCGACCTCGCTTTACTTACCGCAGTGCATCCGGGGCGTAGCCGA
CGTGATGGCCTCTAAGGACCCTATGAACGGTGCGCACGACG
>ITS.332_1-C3 7638_A05_024-A06_024rc.complete
GGAAGTAAAAGTCGTAACAAGGTTTCCGTAGGTGAACCTGCGGAAGGATCATTGTCGTGA
CCCTGACCAAAATAGACCGTGCACGCGTCATCCAGCCTGCCGGCGGCGGCACCGTTCGTC
GCTCGGCCAAGTCCTCGACAACCTCCTCTCCTCGGAGTGGGGGCTCGGGGTAAAAGAACC
CACGACGCCTAAGGCGTCAAGGAACACTGTGCCTAGCCCGGGGACGCGGACGGCTTGCTG
GCCGCCCCCCCGTGCTGCAATGCTATTTAATCCACACGACTCTCGGCAACGGATATCTCG
GCTCTCGCATCGATGAAGAACGTAGCGAAATGCGATACCTGGTGTGAATTGCAGAATCCC
GCGAACCATCGAGTCTTTGAACGCAAGTTGCGCCCGAGGCCATTCGGCTGAGGGCACGCC
TGCCTGGGCGTCACGCAAAACACGCTCCCACACCCTCATCGGGTAGCGGGACGCGGCATG
TGGTCCCCCGTAGCGCAAGCTGCGGTGGGCCGAAGATATGGCTGCCGGCGTATCGTGCCG
GACACAGCGCGTGGTGGGCGACCTCGCTTTACTTACCGCAGTGCATCCGGGGCGTAGCCG
ACGTGATGGCCTCTAAGGACCCTATGAACGGTGCGCACGACG
>ITS.332_1-C4 7638_B05_023-B06_023rc.complete
GGAAGTAAAAGTCGTAACAAGGTTTCCGTAGGTGAACCTGCGGAAGGATCATTGTCGTGA
CCCTGACCAAAATAGACCGTGCACGCGTCATCCAGCCTGCCGGCGGCGGCACCGTTCGTC
GCTCGGCCAAGTCCTCGACAACCTCCTCTCCTCGGAGTGGGGGCTCGGGGTAAAAGAACC
CACGACGCCTAAGGCGTCAAGGAACACTGTGCCTAGCCCGGGGACGCGGACGGCTTGCTG
GCCGCCCCCCGTGCTGCAATGCTATTTAATCCACACGACTCTCGGCAACGGATATCTCGG
CTCTCGCATCGATGAAGAACGTAGCGAAATGCGATACCTGGTGTGAATTGCAGAATCCCG
CGAACCATCGAGTCTTTGAACGCAAGTTGCGCCCGAGGCCATTCGGCTGAGGGCACGCCT
GCCTGGGCGTCACGCAAAACACGCTCCCACACCCTCATCGGGTAGCGGGACGCGGCATGT
GGTCCCCCGTAGCGCAAGCTGCGGTGGGCCGAAGATATGGCTGCCGGCGTATCGTGCCGG
ACACAGCGCGTGGTGGGCGACCTCGCTTTACTTACCGCAGTGCATCCGGGGCGTAGCCGA
CGTGATGGCCTCTAAGGACCCTATGAACGGTGCGCACGACG
>ITS.332_1-C5 7638_C06_022-C05_022rc.complete
GGAAGTAAAAGTCGTAACAAGGTTTCCGTAGGTGAACCTGCGGAAGGATCATTGTCGTGA
CCCTGACCAAAATAGACCGTGCACGCGTCATCCAGCCTGCCGGCGGCGGCACCGTTCGTC
GCTCGGCCAAGTCCTCGACAACCTCCTCTCCTCGGAGTGGGGGCTCGGGGTAAAAGAACC
CACGACGCCTAAGGCGTCAAGGAACACTGTGCCTAGCCCGGGGACGCGGACGGCTTGCTG
GCCGCCCCCCGTGCTGCAATGCTATTTAATCCACACGACTCTCGGCAACGGATATCTCGG

CTCTCGCATCGATGAAGAACGTAGCGAAATGCGATACCTGGTGTGAATTGCAGAATCCCG
CGAACCATCGAGTCTTTGAACGCAAGTTGCGCCCGAGGCCATTCGGTTGAGGGCACGCCT
GCCTGGGCGTCACGCAAAACACGCTCCCACACCCTCATCGGGTAGCGGGACGCGGCATGT
GGTCCCCCGTAGCGCAAGCTGCGGTGGGCCGAAGATATGGCTGCCGGCGTATCGTGCCGG
ACACAGCGCGTGGTGGGCGACCTCGCTTTACTTACCGCAGTGCATCCGGGGCGTAGCCGA
CGTGATGGCCTCTAAGGACCCTATGAACGGTGCGCACGACG
>ITS.332_1-C6 7638_E05_020-D05_021rc.complete
GGAAGTAAAAGTCGTAACAAGGTTTCCGTAGGTGAACCTGCGGAAGGATCATTGTCGTGA
CCCTGACCAAAATAGACCGTGCACGCGTCATCCAGCCTGCCGGCGGCGGCACCGTTCGTC
GCTCGGCCAAGTCCTCGACAACCTCCTCTCCTCGGAGTGGGGGCTCGGGGTAAAAGAACC
CACGACGCCTAAGGCGTCAAGGAACACTGTGCCTAGCCCGGGGACGCGGACGGCTTGCTG
GCCGCCCCCCGTGCTGCAATGCTATTTAATCCACACGACTCTCGGCAACGGATATCTCGG
CTCTCGCATCGATGAAGAACGTAGCGAAATGCGATACCTGGTGTGAATTGCAGAATCCCG
CGAACCATCGAGTCTTTGAACGCAAGTTGCGCCCGAGGCCATTCGGTTGAGGGCACGCCT
GCCTGGGCGTCACGCAAAACACGCTCCCACACCCTCATCGGGTAGCGGGACGCGGCATGT
GGTCCCCCGTAGCGCAAGCTGCGGTGGGCCGAAGATATGGCTGCCGGCGTATCGTGCCGG
ACACAGCGCGTGGTGGGCGACCTCGCTTTACTTACCGCAGTGCATCCGGGGCGTAGCCGA
CGTGATGGCCTCTAAGGACCCTTTGAACGGTGCGCACGACG
>ITS.332_1-C7 7638_E06_020-E05_020rc.complete
GGAAGTAAAAGTCGTAACAAGGTTTCCGTAGGTGAACCTGCGGAAGGATCATTGTCGTGA
CCCTGACCAAAATAGACCGTGCACGCGTCATCCAGCCTGCCGGCGGCGGCACCGTTCGTC
GCTCGGCCAAGTCCTCGACAACCTCCTCTCCTCGGAGTGGGGGCTCGGGGTAAAAGAACC
CACGACGCCTAAGGCGTCAAGGAACACTGTGCCTAGCCCGGGGACGCGGACGGCTTGCTG
GCCGCCCCCCGTGCTGCAATGCTATTTAATCCACACGACTCTCGGCAACGGATATCTCGG
CTCTCGCATCGATGAAGAACGTAGCGAAATGCGATACCTGGTGTGAATTGCAGAATCCCG
CGAACCATCGAGTCTTTGAACGCAAGTTGCGCCCGAGGCCATTCGGTTGAGGGCACGCCT
GCCTGGGCGTCACGCAAAACACGCTCCCACACCCTCATCGGGTAGCGGGACGCGGCATGT
GGTCCCCCGTAGCGCAAGCTGCGGTGGGCCGAAGATATGGCTGCCGGCGTATCGTGCCGG
ACACAGCGCGTGGTGGGCGACCTCGCTTTACTTACCGCAGTGCATCCGGGGCGTAGCCGA
CGTGATGGCCTCTAAGGACCCTTTGAACGGTGCGCACGACG
>ITS.332_1-C8 7638_F06_019-F05_019rc.complete
GGAAGTAAAAGTCGTAACAAGGTTTCCGTAGGTGAACCTGCGGAAGGATCATTGTCGTGA
CCCTGACCAAAATAGACCGTGCACGCGTCATCCAGCCTGCCGGCGGCGGCACCGTTCGTC
GCTCGGCCAAGTCCTCGACAACCTCCTCTCCTCGGAGTGGGGGCTCGGGGTAAAAGAACC
CACGACGCCTAAGGCGTCAAGGAACACTGTGCCTAGCCCGGGGACGCGGACGGCTTGCTG
GCCGCCCCCCGTGCTGCAATGCTATTTAATCCACACGACTCTCGGCAACGGATATCTCGG
CTCTCGCATCGATGAAGAACGTAGCGAAATGCGATACCTGGTGTGAATTGCAGAATCCCG
CGAACCATCGAGTCTTTGAACGCAAGTTGCGCCCGAGGCCATTCGGTTGAGGGCACGCCT
GCCTGGGCGTCACGCAAAACACGCTCCCACACCCTCATCGGGTAGCGGGACGCGGCATGT
GGTCCCCCGTAGCGCAAGCTGCGGTGGGCCGAAGATATGGCTGCCGGCGTATCGTGCCGG
ACACAGCGCGTGGTGGGCGACCTCGCTTTACTTACCGCAGTGCATCCGGGGCGTAGCCGA
CGTGATGGCCTCTAAGGACCCTTTGAACGGTGCGCACGACG
>ITS.404_1-C1 7638_G02_002-G03_010rc.complete
GGAAGTAAAAGTCGTAACAAGGTTTCCGTAGGTGAACCTGCGGAAGGATCATTGTCGTGA
CCCTGACCAAAATAGACCGCGCACGCGTAATCCAGCCTGCCGGCGATGGCATCGTCCGTC
GCTCGGCCAAGTCCTCGACAACCTCATCTCCTCGGAGTGGGGCTCGGGGTAAAAGAACCC
ACGACGCCTAAGGCGTCAAGGAACACTGTGCCTAGCCCGGGGACGCGGACGGCTTGCTGG
CCGCCCCCCGTGCTGCAATGCTATTTAATCCACACGACTCTCGGCAACGGATATCTCGGC
TCTCGCATCGATGAAGAACGTAGCGAAATGCGATACCTGGTGTGAATTGCAGAATCCCGC
GAACCATCGAGTCTTTGAACGCAAGTTGCGCCCGAGGCCATTCGGCTGAGGGCACGCCTG
CCTGGGCGTCACGCAAAACACGCTCCCACCCCCTCATCGGGTAGCGGGACGCGGCATGTG
GTCCCCCGTAGCGCAAGCTGCGGTGGGCCGAAGATATGGCTGCCGGCGTATCGTGCCGGA
CACAGCGCGTGGTGGGCGACCTCGCTTTACTTACCGCAGTGCATCCGGGGCGTAGCTGAC
GTGATGGCCTCTAAGGACCCTATGAACGGTGCGCACGACG

>ITS.404_1-C2 7638_H03_009-H02_001rc.complete
GGAAGTAAAAGTCGTAACAAGGTTTCCGTAGGTGAACCTGCGGAAGGATCATTGTCGTGA
CCCTGACCAAAATAGACCGTGCACGCGTCATCCAGCCTGCCGGCGGCGGCACCGTTCGTC
GCTCGGCCAAGTCCTCGACAACCTCCTCTCCTCGGAGTGGGGGCTCGGGGTAAAAGAACC
CACGACGCCTAAGGCGTCAAGGAACACTGTGCCTAGCCCGGGGACGCGGACGGCTTGCTG
GCCGCCCCCCGTGCTGCAATGCTATTTAATCCACACGACTCTCGGCAACGGATATCTCGG
CTCTCGCATCGATGAAGAACGTAGCGAAATGCGATACCTGGTGTGAATTGCAGAATCCCG
CGAACCATCGAGTCTTTGAACGCAAGTTGCGCCCGAGGCCATTCGGCTGAGGGCACGCCT
GCCTGGGCGTCACGCAAAACACGCTCCCACCCCCTCATCGGGTAGCGGGACGCGGCATGT
GGCCCCCCGTAGCGCAAGCTGCGGTGGGCCGAAGATATGGCTGCCGGCGTATCGTGCCGG
ACACAGCGCGTGGTGAGCGACCTCGCTTTACTTACCGCAGTGCATCCGGGGCGTAGCCGA
CGTGATGGCCTCTAAGGACCCTATGAACGGTGCGCACGACG
>ITS.404_1-C3 7638_A04_016-A03_016rc.complete
GGAAGTAAAAGTCGTAACAAGGTTTCCGTAGGTGAACCTGCGGAAGGATCATTGTCGTGA
CCCTGACCAAAATAGACCGCGCACACGTAATCCAGCCTGCCGGCGATGGCACCGTCCGTC
GCTCGGCCAAGTCCTCGACAACCTCATCTCCTCGGAGTGGGGCTCGGGGTAAAAGAACCC
ACGACGCCTAAGGCGTCAAGGAACACTGTGCCTAGCCCGGGGACGCGGACGGCTTGCTGG
CCGCCCCCCGTGCTGCAATGCTATTTAATCCACACGACTCTCGGCAACGGATATCTCGGC
TCTCGCATCGATGAAGAACGTAGCGAAATGCGATACCTGGTGTGAATTGCAGAATCCCGC
GAACCATCGAGTCTTTGAACGCAAGTTGCGCCCGAGGCCATTCGGCTGAGGGCACGCCTG
CCTGGGCGTCACGCAAAACACGCTCCCACCCCCTCATCGGGTAGCGGGACGCGGCATGTG
GCCCCCCGTAGCGCAAGCTGCGGTGGGCCGAAGATATGGCTGCCGGCGTATCGTGCCGGA
CACAGCGCGTGGTGAGCGACCTCGCTTTACTTACCGCAGTGCATCCGGGGCGTAGCCGAC
GTGATGGCCTCCAAGGACCCTTTGAACGGTGCGCACGACG
>ITS.404_1-C4 7638_B04_015-B03_015rc.complete
GGAAGTAAAAGTCGTAACAAGGTTTCCGTAGGTGAACCTGCGGAAGGATCATTGTCGTGA
CCCTGACCAAAATAGACCGTGCACGCGTCATCCAGCCTGCCGGCGGCGGCACCGTTCGTC
GCTCGGCCAAGTCCTCGACAACCTCCTCTCCTCGGAGTGGGGGCTCGGAGTAAAAGAACC
CACGACGCCTAAGGCGTCAAGGAACACTGTGCCTAGCCCGGGGACGCGGACGGCTTGCTG
GCCGCCCCCCGTGCTGCAATGCTATTTAATCCACACGACTCTCGGCAACGGATATCTCGG
CTCTCGCATCGATGAAGAACGTAGCGAAATGCGATACCTGGTGTGAATTGCAGAATCCCG
CGAACCATCGAGTCTTTGAACGCAAGTTGCGCCCGAGGCCATTCGGCTGAGGGCACGCCT
GCCTGGGCGTCACGCGAAACACGCTCCCACACCCTCATCGGGTAGCGGGACGCGGCATGT
GGTCCCCCGTAGCGCAAGCTGCGGTGGGCCGAAGATATGGCTGCCGGCGTATCGTGCCGG
ACACAGCGCGTGGTGGGCGACCTCGCTTTACTTACCGCAGTGCATCCGGGGCGTAGCCGA
CGTGATGGCCTCTAAGGACCCTTTGAACGGTGCGCACGACG
>ITS.404_1-C5 7638_C04_014-C03_014rc.complete
GGAAGTAAAAGTCGTAACAAGGTTTCCGTAGGTGAACCTGCGGAAGGATCATTGTCGTGA
CCCTGACCAAAATAGACCGTGCACGCGTCATCCAGCCTGCCGGCGGCGGCACCGTTCGTC
GCTCGGCCAAGTCCTCGACAACCTCCTCTCCTCGGAGTGGGGGCTCGGGGTAAAAGAACC
CACGACGCCTAAGGCGTCAAGGAACACTGTGCCTAGCCCGGGGACGCGGACGGCTTGCTG
GCCGCCCCCCGTGCTGCAATGCTATTTAATCCACACGACTCTCGGCAACGGATATCTCGG
CTCTCGCATCGATGAAGAACGTAGCGAAATGCGATACCTGGTGTGAATTGCAGAATCCCG
CGAACCATCGAGTCTTTGAACGCAAGTTGCGCCCGAGGCCATTCGGCTGAGGGCACGCCT
GCCTGGGCGTCACGCAAAACACGCTCCCACCCCCTCATCGGGTAGCGGGACGCGGCATGT
GGTCCCCCGTAGCGCAAGCTGCGGTGGGCCGAAGATATGGCTGCCGGCGTATCGTGCCGG
ACACAGCGCGTGGTGGGCGACCTCGCTTTACTTACCGCAGTGCATCCGGGGCGTAGCCGA
CGTGATGGCCTCTAAGGACCCTTTGAACGGTGCGCACGACG
>ITS.404_1-C6 7638_D03_013-D04_013rc.complete
GGAAGTAAAAGTCGTAACAAGGTTTCCGTAGGTGAACCTGCGGAAGGATCATTGTCGTGA
CCCTGACCAAAATAGACCGCGCACGCGTAATCCAGCCTGCCGGCGATGGCACCGTCCGTC
GCTCGGCCAAGTCCTCGACAACCTCATCTCCTCGGAGTGGGGCTCGGGGTAAAAGAACCC
ACGACGCCTAAGGCGTCAAGGAACACTGTGCCTAGCCCGGGGACGCGGACGGCTTGCTGG
CCGCCCCCCGTGCTGCAATGCTATTTAATCCACACGACTCTCGGCAACGGATATCTCGGCT

CTCGCATCGATGAAGAACGTAGCGAAATGCGATACCTGGTGTGAATTGCAGAATCCCGCG
AACCATCGAGTCTTTGAACGCAAGTTGCGCCCGAGGCCATTCGGCTGAGGGCACGCCTGC
CTGGGCGTCACGCAAAACACGCTCCCACCCCCTCATCGGGTAGCGGGACGCGGCATGTGG
CCCCCCGTAGCGCAAGCTGCGGTGGGCCGAAGATATGGCTGCCGGCGTATCGTGCCGGAC
ACAGCGCGTGGTGAGCGACCTCGCTTTACTTACCGCAGTGCATCCGGGGCGTAGCCGACG
TGATGGCCTCTAAGGACCCTATGAACGGTGCGCACGACG
>ITS.404_1-C7 7638_D04_012-E03_012rc.complete
GGAAGTAAAAGTCGTAACAAGGTTTCCGTAGGTGAACCTGCGGAAGGATCATTGTCGTGA
CCCTGACCAAAATAGACCGCGCACACGTAATCCAGCCTGCCGGCGATGGCACCGTCCATT
GCTCGGCCAAGTCCTCGACAACCTCATCTCCTCGGAGTGGGGCTCGGGGTAAAAGAACCC
ACGACGCCTAAGGCGTCAAGGAACACTGTGCCTAGCCCGGGGACGCGGACGGCTTGCTGG
CCGCCCCCCGTGCTGCAATGCTATTTAATCCACACGACTCTCGGCAACGGATATCTCGGC
TCTCGCATCGATGAAGAACGTAGCGAAATGCGATACCTGGTGTGAATTGCAGAATCCCGC
GAACCATCGAGTCTTTGAACGCAAGTTACGCCTGAGGCCATTCGGCTGAGGGCATGCCTG
TCTAGGCGTCATGGAAAACACACTCCCACCCCCTCATCAGGTAGCGGGACGCGGCATGTG
GCCCCCCGTAGCGCAAGCTGCGGTGGGCCAAAGATATGGCTGCCGGCGTATCGTGCCGGA
CACAGTGCGTGGTGAGCAACCTCGCTTTACTTACCGCAGTGCATCCGGGGCGTAGCCGAC
GTGATGGCCTCCAAGGACCCTTTGAACGGTGCGCACGACG
>ITS.404_1-C8* 7638_F03_011-F04_011rc.complete
GGAAGTAAAAGTCGTAACAAGGTTTCCGTAGGTGAACCTGCGGAAGGATCATTGTCGTGA
CCCTGACCAAAATAGACCGCGCACGCGTAATCCAGCCTGCCGGCGATGGCATCGTCCGTC
GCCCGGCCAAGTCCTCGACAACCTCATCTCCTCGGAGTGGGGCTCGGGGTAAAAGAACCC
ACGACGCCTAAGGCGTCAAGGAACACTGTGCCTAGCCCGGGGACGCGGACGGCTTGCTGG
CCGCCCCCCGTGCTGCAATGCTATTTAATCCACACGACTCTCGGCAACGGATATCTCGGC
TCTCGCATCGATGAAGAACGTAGCGAAATGCGATACCTGGTGTGAATTGCAGAATCCCGC
GAACCATCGAGTCTTTGAACGCAAGTTGCGCCCGAGGCCATTCGGCTGAGGGCACGCCTG
CCTGGGCGTCACGCAAAACACGCTCCCACCCCCTCATCGGGTAGCGGGACGCGGCATGTG
GCCCCCCGTAGCGCAAGCTGCGGTGGGCCGAAGATATGGCTGCCGGCGTATCGTGCCGGA
CACAGCGCGTGGTGAGCGACCTCGCTTTACTTACCGCAGTGCATCCGGGGCGTAGCCGAC
GTGATGGCCTCCAAGGACCCTATGAACGGTGCGCACGACG
>ITS.405-C1 7638_H12_041-F12_043rc.complete
GGAAGTAAAAGTCGTAACAAGGTTTCCGTAGGTGAACCTGCGGAAGGATCATTGTCGTGA
CCCTGACCAAAATAGACCGTGCACGCGTCATCCAGCCTGCCGGCGGCGGCACCGTTCGTC
GCTCGGCCAAGTCCTCGACAACCTCCTCTCCTCGGAGTGGGGGCTCGGGGTAAAAGAACC
CACGACGCCTAAGGCGTCAAGGAACACTGTGCCTAGCCCGGGGACGCGGACGGCTTGCTG
GCCGCCCCCCGTGCTGCAATGCTATTTAATCCACACGACTCTCGGCAACGGATATCTCGG
CTCTCGCATCGATGAAGAATGTAGCGAAATGCGATACCTGGTGTGAATTGCAGAATCCCG
CGAACCATCGAGTCTTTGAACGCAAGTTGCGCCCGAGGCCATTCGGCTGAGGGCACGCCT
GCCTGGGCGTCACGCAAAACACGCTCCCACACCCTCATCGGGTAGCGGGACGCGGCATGT
GGTCCCCCGTAGCGCAAGCTGCGGTGGGCCGAAGATATGGCTGCCGGCGTATCGTGCCGG
ACACAGCGCGTGGTGGGCGACCTCGCTTTACTTACCGCAGTGCATCCGGGGCGTAGCCGA
CGTGATGGCCTCTAAGGACCCTTTGAACGGTGCGCACGACG
>ITS.405-C2 7638_G12_042-H01_001rc.complete
GGAAGTAAAAGTCGTAACAAGGTTTCCGTAGGTGAACCTGCGGAAGGATCATTGTCGTGA
CCCTGACCAAAATAGACCGTGCACGCGTCATCCAGCCTGCCGGCGGCGGCACCGTTCGTC
GCTCGGCCAAGTCCTCGACAGCCTCCTCTCCTCGGAGTGGGGGCTCGGGGTAAAAGAACC
CACGACGCCTAAGGCGTCAAGGAACACTGTGCCTAGCCCGGGGACGCGGACGGCTTGCTG
GCCGCCCCCCGTGCTGCAATGCTATTTAATCCACACGACTCTCGGCAACGGATATCTCGG
CTCTCGCATCGATGAAGAACGTAGCGAAATGCGATACCTGGTGTGAATTGCAGAATCCCG
CGAACCATCGAGTCTTTGAACGCAAGTTGCGCCCGAGGCCATTCGGTTGAGGGCACGCCT
GCCTGGGCGTCACGCAAAACACGCTCCCACACCCTCATCGGGTAGCGGGGCGCGGCATGT
GGTCCCCCGTAGCGCAAGCTGCGGTGGGCCGAAGATATGGCTGCCGGCGTATCGTGCCGG
ACACAGCGCGTGGTGGGCGACCTCGCTTTACTTACCGCAGTGCATCCGGGGCGTAGCCGA
CGTGATGGCCTCTAAGGACCCTATGAACGGTGCGCACGACG

>ITS.405-C3 7638_A02_008-B01_007rc.complete
GGAAGTAAAAGTCGTAACAAGGTTTCCGTAGGTGAACCTGCGGAAGGATCATTGTCGTGA
CCCTGACCAAAATAGACCGTGCACGCGTCATCCAGCCTGCCGGCGGCGGCACCGTTCGTC
GCTCGGCCAAGTCCTCGACAACCTCCTCTCCTCGGAGTGGGGGCTCGGGGTAAAAGAACC
CACGACGCCTAAGGCGTCAAGGAACACTGTGCCTAGCCCGGGGACGCGGACGGCTTGCTG
GCCGCCCCCCGTGCTGCAATGCTATTTAATCCACACGACTCTCGGCAACGGATATCTCGG
CTCTCGCATCGATGAAGAACGTAGCGAAATGCGATACCTGGTGTGAATTGCAGAATCCCG
CGAACCATCGAGTCTTTGAACGCAAGTTGCGCCCGAGGCCATTCGGCTGAGGGCACGCCT
GCCTGGGCGTCACGCAAAACACGCTCCCACACCCTCATCGGGTAGCGGGACGCGGCATGT
GGTCCCCCGTAGCGCAAGCTGCGGTGGGCCGAAGATATGGCTGCCGGCGTATCGTGCCGG
ACACAGCGCGTGGTGGGCGACCTCGCTTTACTTACCGCAGTGCATCCGGGGCGTAGCCGA
CGTGATGGCCTCTAAGGACCCTATGAACGGTGCGCACGACG
>ITS.405-C4 7638_B02_007-C01_006rc.complete
GGAAGTAAAAGTCGTAACAAGGTTTCCGTAGGTGAACCTGCGGAAGGATCATTGTCGTGA
CCCTGACCAAAATAGACCGTGCACGCGTCATCCAGCCTGCCGGCGGCGGCACCGTTCGTC
GCTCGGCCAAGTCCTCGACAACCTCCTCTCCTCGGAGTGGGGGCTCGGGGTAAAAGAACC
CACGACGCCTAAGGCGTCAAGGAACACTGTGCCTAGCCCGGGGACGCGGACGGCTTGCTG
GCCGCCCCCCGTGCTGCAATGCTATTTAATCCACACGACTCTCGGCAACGGATATCTCGG
CTCTCGCATCGATGAAGAACGTAGCGAAATGCGATACCTGGTGTGAATTGCAGAATCCCG
CGAACCATCGAGTCTTTGAACGCAAGTTGCGCCCGAGGCCATTCGGCTGAGGGCACGCCT
GCCTGGGCGTCACGCAAAACACGCTCCCACACCCTCATCGGGTAGCGGGACGCGGCATGT
GGTCCCCCGTAGCGCAAGCTGCGGTGGGCCGAAGATATGGCTGCCGGCGTATCGTGCCGG
ACACAGCGCGTGGTGGGCGACCTCGCTTTACTTACCGCAGTGCATCCGGGGCGTAGCCGA
CGTGATGGCCTCTAAGGACCCTATGAACGGTGCGCACGACG
>ITS.405-C5 7638_C02_006-D01_005rc.complete
GGAAGTAAAAGTCGTAACAAGGTTTCCGTAGGTGAACCTGCGGAAGGATCATTGTCGTGA
CCCTGACCAAAATAGACCGTGCACGCGTCATCCAGCCTGCCGGCGGCGGCACCGTTCGTC
GCTCGGCTAAGTCCTCGACAACCTCCTCTCCTCGGAGTGGGGGCTCGGGGTAAAAGAACC
CACGACGCCTAAGGCGTCAAGGAACACTGTGCCTAGCCCGGGGACGCGGACGGCTTGCTG
GCCGCCCCCCGTGCTGCAATGCTATTTAATCCACACGACTCTCGGCAACGGATATCTCGG
CTCTCGCATCGATGAAGAACGTAGCGAAATGCGATACCTGGTGTGAATTGCAGAATCCCG
CGAACCATCGAGTCTTTGAACGCAAGTTGCGCCCGAGGCCATTCGGCTGAGGGCACGCCT
GCCTGGGCGTCACGCAAAACACGCTCCCACACCCTCATCGGGTAGCGGGACGCGGCATGT
GGTCCCCCGTAGCGCAAGCTGCGGTGGGCCGAAGATATGGCTGCCGGCGTATCGTGCCGG
ACACAGCGCGTGGTGGGCGACCTCGCTTTACTTACCGCAGTGCATCCGGGGCGTAGCCAA
CGTGATGGCCTCTAAGGACCCTTTGAACGGTGCGCACGACG
>ITS.405-C6 7638_D02_005-E01_004rc.complete
GGAAGTAAAAGTCGTAACAAGGTTTCCGTAGGTGAACCTGCGGAAGGATCATTGTCGTGA
CCCTGACCAAAATAGACCGTGCACGCGTCATCCAGCCTGCCGGCGGCGGCACCGTTCGTC
GCTCGGCCAAGTCCTCGACAACCTCCTCTCCTCGGAGTGGGGGCTCGGGGTAAAAGAACC
CACGACGCCTAAGGCGTCAAGGAACACTGTGCCTAGCCCGGGGACGCGGACGGCTTGCTG
GCCGCCCCCCGTGCTGCAATGCTATTTAATCCACACGACTCTCGGCAACGGATATCTCGG
CTCTCGCATCGATGAAGAACGTAGCGAAATGCGATACCTGGTGTGAATTGCAGAATCCCG
CGAACCATCGAGTCTTTGAACGCAAGTTGCGCCCGAGGCCATTCGGCTGAGGGCACGCCT
GCCTGGGCGTCACGCAAAACACGCTCCCACACCCTCATTGGGTAGCGGGACGCGGCATGT
GGTCCCCCGTAGCGCAAGCTGCGGTGGGCCGAAGATATGGCTGCCGGCGTATCGTGCCGG
ACACAGCGCGTGGTGGGCGACCTCGCTTTACTTACCGCAGTGCATCCGGGGCGTAGCCGA
CGTGATGGCCTCTAAGGACCCTATGAACGGTGCGCACGACG
>ITS.405-C7 7638_F01_003-E02_004rc.complete
GGAAGTAAAAGTCGTAACAAGGTTTCCGTAGGTGAACCTGCGGAAGGATCATTGTCGTGA
CCCTGACCAAAATAGACCGTGCACGCGTCATCCAGCCTGCCGGCGGCGGCACCGTTCGTC
GCTCGGCTAAGTCCTCGACAACCTCCTCTCCTCGGAGTGGGGGCTCGGGGTAAAAGAACC
CACGACGCCTAAGGCGTCAAGGAACACTGTGCCTAGCCCGGGGACGCGGACGGCTTGCTG
GCCGCCCCCCGTGCTGCAATGCTATTTAATCCACACGACTCTCGGCAACGGATATCTCGG

CTCTCGCATCGATGAAGAACGTAGCGAAATGCGATACCTGGTGTGAATTGCAGAATCCCG
CGAACCATCGAGTCTTTGAACGCAAGTTGCGCCCGAGGCCATTCGGCTGAGGGCACGCCT
GCCTGGGCGTCACGCAAAACACGCTCCCACACCCTCATCGGGTAGCGGGACGCGGCATGT
GGTCCCCCGTAGCGCAAGCTGCGGTGGGCCGAAGATATGGCTGCCGGCGTATCGTGCCGG
ACACAGCGCGTGGTGGGCGACCTCGCTTTACTTACCGCAGTGCATCCGGGGCGTAGCCAA
CGTGATGGCCTCTAAGGACCCTATGAACGGTGCGCACGACG
>ITS.405-C8 7638_G01_002-F02_003rc.complete
GGAAGTAAAAGTCGTAACAAGGTTTCCGTAGGTGAACCTGCGGAAGGATCATTGTCGTGA
CCCTGACCAAAATAGACCGTGCACGCGTCATCCAGCCTGCCGGCGGCGGCACCGTTCGTC
GCTCGGCCAAGTCCTCGACAACCTCCTCTCCTCGGAGTGGGGGCTCGGGGTAAAAGAACC
CACGACGCCTAAGGCGTCAAGGAACACTGTGCCTAGCCCGGGGACGCGGACGGCTTGCTG
GCCGCCCCCCGTGCTGCAATGCTATTTAATCCACACGACTCTCGGCAACGGATATCTCGG
CTCTCGCATCGATGAAGAACGTAGCGAAATGCGATACCTGGTGTGAATTGCAGAATCCCG
CGAACCATCGAGTCTTTGAACGCAAGTTGCGCCCGAGGCCATTCGGCTGAGGGCACGCCT
GCCTGGGCGTCACGCAAAACACGCTCCCACACCCTCATCGGGTAGCGGGACGCGGCATGT
GGTCCCCCGTAGCGCAAGCTGCGGTGGGCCGAAGATATGGCTGCCGGCGTATCGTGCCGG
ACACAGCGCGTGGTGGGCGACCTCGCTTTACTTACCGCAGTGCATCCGGGGCGTAGCCAA
CGTGATGGCCTCTAAGGACCCTATGAACGGTGCGCACGACG
>ITS.483-C1 7638_49rc-57
GGAAGTAAAAGTCGTAACAAGGTTTCCGTAGGTGAACCTGCGGAAGGATCATTGTCGTGA
CCCTGACCAAAATAGACCGCGCACGCGTAATCCAGCCTGCCGGCGATGGCATCGTCCGTC
GCTCGGCCAAGTCCTCGACAACCTCATCTCCTCGGAGTGGGGCTCGGGGTAAAAGAACCC
ACGACGCCTAAGGCGTCAAGGAACACTGTGCCTAGCCCGGGGACGCGGACGGCTTGCTGG
CCGCCCCCCGTGCTGCAATGCTATTTAATCCACACGACTCTCGGCAACGGATATCTCGGC
TCTCGCATCGATGAAGAACGTAGCGAAATGCGATACCTGGTGTGAATTGCAGAATCCCGC
GAACCATCGAGTCTTTGAACGCAAGTTGCGCCCGAGGCCATTCGGCTGAGGGCACGCCTG
CCTGGGCGTCACGCAAAACACGCTCCCACCCCCTCATCGGGTAGAGGGACGCGGCATGTG
GCCCCCCGTAGCGCAAGCTGCGGTGGGCCGAAGATATGGCTGCCGGCGTATCGTGCCGGA
CACAGCGTGTGGTGAGCGACCTCGCTTTACTTACCGCAGTGCATCCGGGGCGTAGCCGAC
GTGATGGCCTCCAAGGACCCTATGAACGGTGCGCACGACG
>ITS.483-C2 7638_50-58rc
GGAAGTAAAAGTCGTAACAAGGTTTCCGTAGGTGAACCTGCGGAAGGATCATTGTCGTGA
CCCTGACCAAAATAGACCGCGCACGCGTAATCCAGCCTGCCGGCGATGGCATCGTCCGTC
GCTCGGCCAAGTCCTCGACAACCTCATCTCCTCGGAGTGGGGCTCGGGGTAAAAGAACCC
ACGACGCCTAAGGCGTCAAGGAACACTGTGCCTAGCCCGGGGACGCGGACGGCTTGCTGG
CCGCCCCCCGTGCTGCAATGCTATTTAATCCACACGACTCTCGGCAACGGATATCTCGGC
TCTCGCATCGATGAAGAACGTAGCGAAATGCGATACCTGGTGTGAATTGCAGAATCCCGC
GAACCATCGAGTCTTTGAACGCAAGTTGCGCCCGAGGCCATTCGGCTGAGGGCACGCCTG
CCTGGGCGTCACGCAAAACACGCTCCCACCCCCTCATCGGGTAGCGGGACGCGGCATGTG
GCCCCCCGTAGCGCAAGCTGCGGTGGGCCGAAGATATGGCTGCCGGCGTATCGTGCCGGA
CACAGCGCGTGGTGAGCGACCTCGCTTTACTTACCGCAGTGCATCCGGGGCGTAGCCGAC
GTGATGGCCTCTAAGGACCCTATGAACGGTGCGCACGACG
>ITS.483-C3 7638_51-59rc
GGAAGTAAAAGTCGTAACAAGGTTTCCGTAGGTGAACCTGCGGAAGGATCATTGTCGTGA
CCCTGACCAAAATAGACCGCGCACGCGTAATCCAGCCTGCCGGCGATGGCATCGTCCGTC
GCTCGGCCAAGTCCTCGACAACCTCATCTCCTCGGAGTGGGGCTCGGGGTAAAAGAACCC
ACGACGCCTAAGGCGTCAAGGAACACTGTGCCTAGCCCGGGGACGCGGACGGCTTGCTGG
CCGCCCCCCGTGCTGCAATGCTATTTAATCCACACGACTCTCGGCAACGGATATCTCGGC
TCTCGCATCGATGAAGAACGTAGCGAAATGCGATACCTGGTGTGAATTGCAGAATCCCGC
GAACCATCGAGTCTTTGAACGCAAGTTGCGCCCGAGGCCATTCGGCTGAGGGCACGCCTG
CCTGGGCGTCACGCAAAACACGCTCCCACCCCCTCATCGGGTAGCGGGACGCGGCATGTG
GCCCCCCGTAGCGCAAGCTGCGGTGGGCCGAAGATATGGCTGCCGGCGTATCGTGCCGGA
CACAGCGCGTGGTGAGCGACCTCGCTTTACTTACCGCAGTGCATCCGGGGCGTAGCCGAC
GTGATGGCCTCTAAGGACCCTATGAACGGTGCGCACGACG

>ITS.483-C4 7638_52rc-60
GGAAGTAAAAGTCGTAACAAGGTTTCCGTAGGTGAACCTGCGGAAGGATCATTGTCGTGA
CCCTGACCAAAATAGACCGCGCACGCGTAATCCAGCCTGCCGGCGACGGCACCGTCCGTC
GCTCGGCCAAGTCCTCGACAACCTCATCTCCTCGGAGTGGGGCTCGGGGTAAAAGAACCC
ACGACGCCTAAGGCGTCAAGGAACACTGTGCCTAGCCCGGGGACGCGGACGGCTTGCTGG
CCGCCCCCCGTGCTGCAATGCTATTTAATCCACACGACTCTCGGCAACGGATATCTCGGC
TCTCGCATCGATGAAGAACGTAGCGAAATGCGATACCTGGTGTGAATTGCAGAATCCCGC
GAACCATCGAGTCTTTGAACGCAAGTTGCGCCCGAGGCCATTCGGCTGAGGGCACGCCTG
CCTGGGCGTCACGCAAAACACGCTCCCACCCCCTCATCGGGTAGCGGGACGCGGCATGTG
GCCCCCCGTAGCGCAAGCTGCGGTGGGCCGAAGATATGGCTGCCGGCGTATCGTGCCGGA
CACAGCGTGTGGTGAGCGACCTCGCTTTACTTACCGCAGTGCATCCGGGGCGTAGCCGAC
GTGATGGCCTCCAAGGACCCTATGAACGGTGCGCACGACG
>ITS.483-C5 7638_53-61rc
GGAAGTAAAAGTCGTAACAAGGTTTCCGTAGGTGAACCTGCGGAAGGATCATTGTCGTGA
CCCTGACCAAAATAGACCGCGCACGCGTAATCCAGCCTGCCGGCGATGGCACCGTTCGTC
GCTCGGCCAAGTCCTCGACAACCTCATCTCCTCGGAGTGGGGCTCGGGGTAAAAGAACCC
ACGACGCCTAAGGCGTCAAGGAACACTGTGCCTAGCCCGGGGACGCGGACGGCTTGCTGG
CCGCCCCCGTGCTGCAATGCTATTTAATCCACACGACTCTCGGCAACGGATATCTCGGCT
CTCGCATCGATGAAGAACGTAGCGAAATGCGATATCTGGTGTGAATTGCAGAATCCCGCG
AACCATCGAGTCTTTGAACGCAAGTTGCGCCCGAGGCCATTCGGCTGAGGGCACGCCTGC
CTGGGCGTCACGCAAAACACGCTCCCACCCCCTCATCGGGTAGCGGGACGCGGCATGTGG
CCCCCCGTAGCGCAAGCTGCGGTGGGCCGAAGATATGGCTGCCGGCGTATCGTGCCGGAC
ACAGCGCGTGGTGAGCGACCTCGCTTTACTTACCGCAGTGCATCCGGGGCGTAGCCGACG
TGATGGCCTCCAAGGACCCTATGAACGGTGCGCACGACG
>ITS.483-C6 7638_54-62rc
GGAAGTAAAAGTCGTAACAAGGTTTCCGTAGGTGAACCTGCGGAAGGATCATTGTCGTGA
CCCTGACCAAAATAGACCGCGCACGCGTAATCCAGCCTGCCGGCGATGGCACCGTCCGTC
GCTCGGCCAAGTCCTCGACAACCTCATCTCCTCGGAGTGGGGCTCGGGGTAAAAGAACCC
ACGACGCCTAAGGCGTCAAGGAACACTGTGCCTAGCCCGGGGACGCGGACGGCTTGCTGG
CCGCCCCCCGTGCTGCAATGCTATTTAATCCACACGACTCTCGGCAACGGATATCTCGGC
TCTCGCATCGATGAAGAACGTAGCGAAATGCGATACCTGGTGTGAATTGCAGAATCCCGC
GAACCATCGAGTCTTTGAACGCAAGTTGCGCCCGAGGCCATTCGGCTGAGGGCACGCCTG
CCTGGGCGTCACGCAAAACACGCTCCCACCCCCTCATCGGGTAGCGGGACGCGGCATGTG
GCCCCCCGTAGCGCAAGCTGCGGTGGGCCGAAGATATGGCTGCCGGCGTATCGTGCCGGA
CACAGCGCGTGGTGAGCGACCTCGCTTTACTTACCGCAGTGCATCCGGGGCGTAGCCGAC
GTGATGGCCTCCAAGGACCCTATGAACGGTGCGCACGACG
>ITS.483-C7 7638_55-63rc
GGAAGTAAAAGTCGTAACAAGGTTTCCGTAGGTGAACCTGCGGAAGGATCATTGTCGTGA
CCCTGACCAAAATAGACCGCGCACGCGTAATCCAGCCTGCCGGCGACGGCACCGTCCGTC
GCTCGGCCAAGTCCTCGACAACCTCATCTCCTCGGAGTGGGGCTCGGGGTAAAAGAACCC
ACGACGCCTAAGGCGTCAAGGAACACTGTGCCTAGCCCGGGGACGCGGACGGCTTGCTGG
CCGCCCCCCGTGCTGCAATGCTATTTAATCCACACGACTCTCGGCAACGGATATCTCGGC
TCTCGCATCGATGAAGAACGTAGCGAAATGCGATACCTGGTGTGAATTGCAGAATCCCGC
GAACCATCGAGTCTTTGAACGCAAGTTGCGCCCGAGGCCATTCGGCTGAGGGCACGCCTG
CCTGGGCGTCACGCAAAACACGCTCCCACCCCCTCATCGGGTAGCGGGACGCGGCATGTG
GCCCCCCGTAGCGCAAGCTGCGGTGGGCCGAAGATATGGCTGCCGGCGTATCGTGCCGGA
CACAGCGCGTGGTGAGCGACCTCACCTTACTTACCGCAGTGCATCCGGGGCGTAGCCGAC
GTGATGGCCTCCAAGGACCCTATGAACGGTGCGCACGACG
>ITS.483-C8 7638_56-64rc
GGAAGTAAAAGTCGTAACAAGGTTTCCGTAGGTGAACCTGCGGAAGGATCATTGTCGTGA
CCCTGACCAAAATAGACCGCGCACGCGTAATCCAGCCTGCCGGCGATGGCATCGTCCGTC
GCTCGGCCAAGTCCTCGACAACCTCATCTCCTCGGAGTGGGGCTCGGGGTAAAAGAACCC
ACGACGCCTAAGGCGTCAAGGAACACTGTGCCTAGCCCGGGGACGCGGACGGCTTGCTGG
CCGCCCCCCGTGCTGCAATGCTATTTAACCCACACGACTCTCGGCAACGGATATCTCGGC

TCTCGCATCGATGAAGAACGTAGCGAAATGCGATACCTGGTGTGAATTGCAGAATCCCGC
GAACCATCGAGTCTTTGAACGCAAGTTGCGCCCGAGGCCATTCGGCTGAGGGCACGCCTG
CCTGGGCGTCACGCAAAACACGCTCCCACCCCCTCATCGGGTAGCGGGACGCGGCATGTG
GCCCCCCGTAGCGCAAGCTGCGGTGGGCCGAAGATATGGCTGCCGGCGTATCGTGCCGGA
CACAGCGCGTGGTGAGCGACCTCGCTTTACTTACCGCAGTGCATCCGGGGCGTAGCCGAC
GTGATGGCCTCTAAGGACCCTATGAACGGTGCGCACGACG
>ITS.M008A-C1 7638_65rc-73.complete
GGAAGTAAAAGTCGTAACAAGGTTTCCGTAGGTGAACCTGCGGAAGGATCATTGTCGTGA
CCCTGACCAAAATAGACCGTGCACGCGTCATCCAGCCTGCCGGCGGCGGCACCGTTCGTC
GCTCGGCCAAGTCCTCGACAACCTCCTCTCCTCGGAGTGGGGGCTCGGGGTAAAAGAACC
CACGACGCCTAAGGCGTCAAGGAACACTGTGCCTAGCCCGGGGACGCGGACGGCTTGCTG
GCCGCCCCCCGTGCTGCAATGCTATTTAATCCACACGACTCTCGGCAACGGATATCTCGG
CTCTCGCATCGATGAAGAACGTAGCGAAATGCGATACCTGGTGTGAATTGCAGAATCCCG
CGAACCATCGAGTCTTTGAACGCAAGTTGCGCCCGAGGCCATTCGGCTGAGGGCACGCCT
GCCTGGGCGTCACGCAAAGCACGCTCCCACACCCTCATCGGGTAGCGGGACGCGGCATGT
GGTCCCCCGTAGCGCAAGCTGCGGTGGGCCGAAGATATGGCTGCCGGCGTATCGTGCCGG
ACACAGCGCGTGGTGGGCGACCTCGCTTTACTTACCGCAGTGCATCCGGGGCGTAGCCGA
CGTGATGGCCTCTAAGGACCCTATGAACGGTGCGCACGACG
>ITS.M008A-C2 7638_66rc-74.complete
GGAAGTAAAAGTCGTAACAAGGTTTCCGTAGGTGAACCTGCGGAAGGATCATTGTCGTGA
CCCTGACCAAAATAGACCGTGCACGCGTCATCCAGCCTGCCGGCGGCGGCACCGTTCGTC
GCTCGGCCAAGTCCTCGACAACCTCCTCTCCTCGGAGTGGGGGCTCGGGGTAAAAGAACC
CACGACGCCTAAGGCGTCAAGGAACACTGTGCCTAGCCCGGGGACGCGGACGGCTTGCGG
GCCGCCCCCCGTGCTGCAATGCTATTTAATCCACACGACTCTCGGCAACGGATATCTCGG
CTCTCGCATCGATGAAGAACGTAGCGAAATGCGATACCTGGTGTGAATTGCAGAATCCCG
CGAACCATCGAGTCTTTGAACGCAAGTTGCGCCCGAGGCCATTCGGCTGAGGGCACGCCT
GCCTGGGCGTCACGCAAAACACGCTCCCACACCCTCATCGGGTAGCGGGACGCGGCTTGT
GGTTCCCCGTAGCGCAAGCTGCGGTGGGCCGAAGATATGGCTGCCGGCGTATCGTGCCGG
ACACAGCGCGTGGTGGGCGACCTCGCTTTACTTACCGCAGTGCATCCGGGGCGTAGCCGA
CGTGATGGCCTCTAAGGACCCTTTGAACGGTGCGCACGACG
>ITS.M008A-C3 7638_67-75rc.complete
GGAAGTAAAAGTCGTAACAAGGTTTCCGTAGGTGAACCTGCGGAAGGATCATTGTCGTGA
CCCTGACCAAAATAGACCGTGCACGCGTCATCCAGCCTGCCGGCGGCGGCACCGTTCGTC
GCTCGGCCAAGTCCTCGACAACCTCCTCTCCTCGGAGTGGGGGCTCGGGGTAAAAGAACC
CACGACGCCTAAGGCGTCAAGGAACACTGTGCCTAGCTCGGGGACGCGGACGGCTTGCTG
GCCGCCCCTCGTGCTGCAATGCTATTTAATCCACACGACTCTCGGCAACGGATATCTCGG
CTCTCGCATCGATGAAGAACGTAGCGAAATGCGATACCTGGTGTGAATTGCAGAATCCCG
CGAACCATCGAGTCTTTGAACGCAAGTTGCGCCCGAGGCCATTCGGCTGAGGGCACGCCT
GCCTGGGCGTCACGCAAAACACGCTCCCACACCCTCATCGGGTAGCGGGACGCGGCATGT
GGTCCCCCGTAGCGCAAGCTGCGGTGGGCCGAAGATATGGCTGCCGGCGTATCGTGCCGG
ACACAGCGCGTGGTGGGCGACCTCGCTTTACTTACCGCAGTGCATCCGGGGCGTAGCCGA
CGTGATGGCCTCTAAGGACCCTATGAACGGTGCGCACGACG
>ITS.M008A-C4 7638_68-76rc.complete
GGAAGTAAAAGTCGTAACAAGGTTTCCGTAGGTGAACCTGCGGAAGGATCATTGTCGTGA
CCCTGACCAAAATAGACCGTGCACGCGTCATCCAGCCTGCCGGCGGCGGCACCGTTCGTC
GCTCGGCCAAGTCCTCGACAACCTCCTCTCCTCGGAGTGGGGGCTCGGGGTAAAAGAACC
CACGACGCCTAAGGCGTCAAGGAACACTGTGCCTAGCCCGGGGACGCGGACGGCTTGCTG
GCCGCCCCCCGTGCTGCAATGCTATTTAATCCACACGACTCTCGGCAACGGATATCTCGG
CTCTCGCATCGATGAAGAACGTAGCGAAATGCGATACCTGGTGTGAATTGCAGAATCCCG
CGAACCATCGAGTCTTTGAACGCAAGTTGCGCCCGAGGCCATTCGGCTGAGGGCACGCCT
GCCTGGGCGTCACGCAAAACACGCTCCCACACCCTCATCGGGTAGCGGGACGCGGCATGT
GGTCCCCCGTAGCGCAAGCTGCGGTGGGCCGAAGATATGGCTGCCGGCGTATCGTGCCGG
ACACAGCGCGTGGTGGGCGACCTCGCTTTACTTACCGCAGTGCATCCGGGGCGTAGCCGA
CGTGATGGCCTCTAAGGACCCTATGAACGGTGCGCACGACG

>ITS.M008A-C5 7638_69-77rc.complete
GGAAGTAAAAGTCGTAACAAGGTTTCCGTAGGTGAACCTGCGGAAGGATCATTGTCGTGA
CCCTGACCAAAATAGACCGTGCACGCGTCATCCAGCCTGCCGGCGGCGGCACCGTTCGTC
GCTCGGCCAAGTCCTCGACAACCTCCTCTCCTCGGAGTGGGGGCTCGGGGTAAAAGAACC
CACGACGCCTAAGGCGTCAAGGAACACTGTGCCTAGCCCGGGGACGCGGACGGCTTGCTG
GCCGCCCCCCGTGCTGCAATGCTATTTAATCCACACGACTCTCGGCAACGGATATCTCGG
CTCTCGCATCGATGAAGAACGTAGCGAAATGCGATACCTGGTGTGAATTGCAGAATCCCG
CGAACCATCGAGTCTTTGAACGCAAGTTGCGCCCGAGGCCATTCGGCTGAGGGCACGCCT
GCCTGGGCGTCACGCAAAACACGCTCCCACACCCTCATCGGGTAGCGGGACGCGGCATGT
GGTCCCCCGTAGCGCAAGCTGCGGTGGGCCGAAGATATGGCTGCCGGCGTATCGTGCCGG
ACACAGCGCGTGGTGGGCGACCTCGCTTTACTTACCGCAGTGCATCCGGGGCGTAGCCGA
CGTGATGGCCTCTAAGGACCCTATGAACGGTGCGCACGACG
>ITS.M008A-C6 7638_70rc-78.complete
GGAAGTAAAAGTCGTAACAAGGTTTCCGTAGGTGAACCTGCGGAAGGATCATTGTCGTGA
CCCTGACCAAAATAGACCGTGCACGCGTCATCCAGCCTGCCGGCGGCGGCACCGTTCGTC
GCTCGGCCAAGTCCTCGACAACCTCCTCTCCTCGGAGTGGGGGCTCGGGGTAAAAGAACC
CACGACGCCTAAGGCGTCAAGGAACACTGTGCCTAGCCCGGGGACGCGGACGGCTTGCTG
GCCGCCCCCCGTGCTGCAATGCTATTTAATCCACACGACTCTCGGCAACGGATATCTCGG
CTCTCGCATCGATGAAGAACGTAGCGAAATGCGATACCTGGTGTGAATTGCAGAATCCCG
CGAACCATCGAGTCTTTGAACGCAAGTTGCGCCCGAGGCCATTCGGCTGAGGGCACGCCT
GCCTGGGCGTCACGCAAAACACGCTCCCACACCCTCATCGGGTAGCGGGACGCGGCTTGT
GGTTCCCCGTAGCGCAAGCTGCGGTGGGCCGAAGATATGGCTGCCGGCGTATCGTGCCGG
ACACAGCGCGTGGTGGGCGACCTCGCTTTACTTACCGCAGTGCATCCGGGGCGTAGCCGA
CGTGATGGCCTCTAAGGACCCTTTGAACGGTGCGCACGACG
>ITS.M008A-C7 7638_71-79rc.complete
GGAAGTAAAAGTCGTAACAAGGTTTCCGTAGGTGAACCTGCGGAAGGATCATTGTCGTGA
CCCTGACCAAAATAGACCGTGCACGCGTCATCCAGCCTGCCGGCGGCGGCACCGTTCGTC
GCTCGGCCAAGTCCTCGACAACCTCCTCTCCTCGGAGTGGGGGCTCGGGGTAAAAGAACC
CACGGCGCCTAAGGCGTCAAGGAACACTGTGCCTAGCTCGGGGACGCGGACGGCTTGCTG
GCCGCCCCTCGTGCTGCAATGCTATTTAATCCACACGACTCTCGGCAACGGATATCTCGG
CTCTCGCATCGATGAAGAACGTAGCGAAATGCGATACCTGGTGTGAATTGCAGAATCCCG
CGAACCATCGAGTCTTTGAACGCAAGTTGCGCCCGAGGCCATTCGGCTGAGGGCACGCCT
GCCTGGGCGTCACGCAAAACACGCTCCCACACCCTCATCGGGTAGCGGGACGCGGCATGT
GGTCCCCCGTAGCGCAAGCTGCGGTGGGCCGAAGATATGGCTGCCGGCGTATCGTGCCGG
ACACAGCGCGTGGTGGGCGACCTCGCTTTACTTACCGCAGTGCATCCGGGGCGTAGCCGA
CGTGATGGCCTCTAAGGACCCTATGAACGGTGCGCACGACG
>ITS.M008A-C8 7638_72rc-80.complete
GGAAGTAAAAGTCGTAACAAGGTTTCCGTAGGTGAACCTGCGGAAGGATCATTGTCGTGA
CCCTGACCAAAATAGACCGTGCACGCGTCATCCAGCCTGCCGGCGGCGGCACCGTTCGTC
GCTCGGCCAAGTCCTCGACAACCTCCTCTCCTCGGAGTGGGGGCTCGGGGTAAAAGAACC
CACGACGCCTAAGGCGTCAAGGAACACTGTGCCTAGCCCGGGGACGCGGACGGCTTGCTG
GCCGCCCCCCGTGCTGCAATGCTATTTAATCCACACGACTCTCGGCAACGGATATCTCGG
CTCTCGCATCGATGAAGAACGTAGCGAAATGCGATACCTGGTGTGAATTGCAGAATCCCG
CGAACCATCGAGTCTTTGAACGCAAGTTGCGCCCGAGGCCATTCGGCTGAGGGCACGCCT
GCCTGGGCGTCACGCAAAACACGCTCCCACACCCTCATCGGGTAGCGGGACGCGGCTTGT
GGTTCCCCGTAGCGCAAGCTGCGGTGGGCCGAAGATATGGCTGCCGGCGTATCGTGCCGG
ACACAGCGCGTGGTGGGCGACCTCGCTTTACTTACCGCAGTGCATCCGGGGCGTAGCCGA
CGTGATGGCCTCTAAGGACCCTTTGAACGGTGCGCACGACG
>ITS.M397-C1 7638_81rc-89.complete
GGAAGTAAAAGTCGTAACAAGGTTTCCGTAGGTGAACCTGCGGAAGGATCATTGTCGTGA
CCCTGACCAAAATAGACCGTGCACGCGTCATCCAGCCTGCCGGCGGCGGCACCGTTCGTC
GCTCGGCCAAGTCCTCGACAACCTCCTCTCCTCGGAGTGGGGGCTCGGGGTAAAAGAACC
CACGACGCCTAAGGCGTCAAGGAACACTGTGCCTAGCCCGGGGACGCGGACGGCTTGCTG
GCCGCCCCCCGTGCTGCAATGCTATTTAATCCACACGACTCTCGGCAACGGATATCTCGG

CTCTCGCATCGATGAAGAACGTAGCGAAATGCGATACCTGGTGTGAATTGCAGAATCCCG
CGAACCATCGAGTCTTTGAACGCAAGTTGCGCCCGAGGCCATTCGGCTGAGGGCACGCCT
GCCTGGGCGTCACGCAAAACATGCTCCCACACCCTCATCGGGTAGCGGGACGCGGCATGT
GGTACCCCGTAGCGCAAGCTGAGGTGGGCCGAAGATATGGCTGCCGGCGTATCGTGCCGG
ACACAGCGCGTGGTGGGCGACCTCGCTTTACTTACCGCAGTGCATCCGGGGCGTAGCCGA
CGTGATGGCCTCTAAGGACCCTTTGAACGGTGCGCACGACG
>ITS.M397-C2 7638_82-90rc.complete
GGAAGTAAAAGTCGTAACAAGGTTTCCGTAGGTGAACCTGCGGAAGGATCATTGTCGTGA
CCCTGACCAAAATAGACCGTGCACGCGTCATCCAGCCTGCCGGCGGCGGCACCGTTCGTC
GCTCGGCCAAGTCCTCGACAACCTCCTCTCCTCGGAGTGGGGGCTCGGGGTAAAAGAACC
CACGACGCCTAAGGCGTCAAGGAACACTGTGCCTAGCCCGGGGACGCGGACGGCTTGCTG
GCCGCCCCCCGTGCTGCAATGCTATTTAATCCACACGACTCTCGGCAACGGATATCTCGG
CTCTCGCATCGATGAAGAACGTAGCGAAATGCGATACCTGGTGTGAATTGCAGAATCCCG
CGAACCATCGAGTCTTTGAACGCAAGTTGCGCCCGAGGCCATTCGGCTGAGGGCACGCCT
GCCTGGGCGTCACGCAAAACACGCTCCCACACCCTCATCGGGTAGCGGGACGCGGCATGT
GGTCCCCCGTAGCGCAAGCTGCGGTGGGCCGAAGATATGGCTGCCGGCGTATCGTGCCGG
ACACAGCGCGTGGTGGGCGACCTCGCTTTACTTACCGCAGTGCATCCGGGGCGTAGCCAA
CGTGATGGCCTCTAAGGACCCTATGAACGGTGCGCACGACG
>ITS.M397-C3 7638_83rc-91.complete
GGAAGTAAAAGTCGTAACAAGGTTTCCGTAGGTGAACCTGCGGAAGGATCATTGTCGTGA
CCCTGACCAAAATAGACCGTGCACGCGTCATCCAGCCTGCCGGCGGCGGCACTGTTCGTC
GCTCGGCCAAGTCCTCGACAACCTCCTCTCCTCGGAGTGGGGGCTCGGGGTAAAAGAACC
CACGACGCCTAAGGCGTCAAGGAACACTGTGCCTAGCCCGGGGACGCGGACGGCTTGCTG
GCCGCCCCCCGTGCTGCAATGCTATTTAATCCACACGACTCTCGGCAACGGATATCTCGG
CTCTCGCATCGATGAAGAACGTAGCGAAATGCGATACCTGGTGTGAATTGCAGAATCCCG
CGAACCATCGAGTCTTTGAACGCAAGTTGCGCCCGAGGCCATTCGGCTGAGGGCACGCCT
GCCTGGGCGTCACGCAAAACACGCTCCCACACCCTCATCGGGTAGCGGGACGCGGCATGT
GGTCCCCCGTAGCGCAAGCTGCGGTGGGCCGAAGATATGGCTGCCGGCGTATCGTGCCGG
ACACAGCGCGTGGTGGGCGACCTCGCTTTACTTACCGCAGTGCATCCGGGGCGTAGCCGA
CGTGATGGCCTCTAAGGACCCTTTGAACGGTGCGCACGACG
>ITS.M397-C4 7638_84rc-92.complete
GGAAGTAAAAGTCGTAACAAGGTTTCCGTAGGTGAACCTGCGAAAGGATCATTGTCGTGA
CGCTGACCAAAATAGACCATGCACGCGTCATCCAGCCTGCCGGCGGCGGCACCGTTCGTC
GCTCGGCCAAGTCCTCGACAACCTCCTCTCCTCGGAGTGGGGGCTCGAGGTAAAAGAACC
CACGACGCCTAAGGCGTCAAGGAACACTGTGCCTAGCCCGGGGACGCGGACGGCTTGCTG
GCCGCCCCTCGTGCTGCAATGCTATTTAATCCACACGACTCTCGGCAACGGATATCTCGG
CTCTCGCATCGATGAAGAACGTAGCGAAATGCGATACCTGGTGTGAATTGCAGAATCCCG
CGAACCATCGAGTCTTTGAACGCAAGTTGCGCCCGAGGCCATTCGGCTGAGGGCACGCCT
GCCTGGGCGTCACGCAAAACACGCTCCCACACCCTCATCGGGTAGCGGGACGCGGCATGT
GGTCCCCCGTAGCGCAAGCTGCGGTGGGCCGAAGATATGGCTGCCGGCGTATCGTGCCGG
ACACAGCGCGTGGTGGGCGACCTCGCTTTACTTACCGCAGTGCATCCGGGGCGTAGCCGA
CGTGATGGCCTCTAAGGACCCTATGAACGGTGCGCACGACG
>ITS.M397-C5 7638_85-93rc.complete
GGAAGTAAAAGTCGTAACAAGGTTTCCGTAGGTGAACCTGCGGAAGGATCATTGTCGTGA
CCCTGACCAAAATAGACCGTGCACGCGTCATCCAGCCTGCCGGCGGCGGCACCGTTCGTC
GCTCGGCCAAGTCCTCGACAACCTCCTCTCCTCGGAGTGGGGGCTCGGGGTAAAAGAACC
CACGACGCCTAAGGCGTCAAGGAACACTGTGCCTAGCCCGGGGACGCGGACGGCTTGCTG
GCCGCCCCCCGTGCTGCAATGCTATTTAATCCACACGACTCTCGGCAACGGATATCTCGG
CTCTCGCATCGATGAAGAACGTAGCGAAATGCGATACCTGGTGTGAATTGCAGAATCCCG
CGAACCATCGAGTCTTTGAACGCAAGTTGCGCCCGAGGCCATTCGGCTGAGGGCACGCCT
GCCTGGGCGTCACGCAAAACACGCTCCCACACCCTCATCGGGTAGCGGGACGCGGCATGT
GGTCCCCCGTAGCGCAAGCTGCGGTGGGCCGAAGATATGGCTGCCGGCGTATCGTGCCGG
ACACAGCGCGTGGTGGGCGACCTCGCTTTACTTACCGCAGTGCATCCGGGGCGTAGCCGA
CGTGATGGCCTCTAAGGACCCTATGAACGGTGCGCACGACG

>ITS.M397-C6 7638_86-94rc.complete
GGAAGTAAAAGTCGTAACAAGGTTTCCGTAGGTGAACCTGCGGAAGGATCATTGTCGTGA
CCCTGACCAAAATAGACCGTGCACGCGTCATCCAGCCTGCCGGCGGCGGCACCGTTCGTC
GCTCGGCCAAGTCCTCGACAACCTCCTCTCCTCGGAGTGGGGGCTCGGGGTAAAAGAACC
CACGACGCCTAAGGCGTCAAGGAACACTGTGCCTAGCCCGGGGACGCGGACGGCTTGCTG
GCCGCCCCCCGTGCTGCAATGCTATTTAATCCACACGACTCTCGGCAACGGATATCTCGG
CTCTCGCATCGATGAAGAACGTAGCGAAATGCGATACCTGGTGTGAATTGCAGAATCCCG
CGAACCATCGAGTCTTTGAACGCAAGTTGCGCCCGAGGCCATTCGGCTGAGGGCACGCCT
GCCTGGGCGTCACGCAAAACACGCTCCCACACCCTCATCGGGTAGCGGGACGCGGCATGT
GGTCCCCCGTAGCGCAAGCTGCGGTGGGCCGAAGATATGGCTGCCGGCGTATCGTGCCGG
ACACAGCGCGTGGTGGGCGACCTCGCTTTACTTACCGCAGTGCATCCGGGGCGTAGCCGA
CGTGATGGCCTCTAAGGACCCTATGAACGGTGCGCACGACG
>ITS.M397-C7 7638_87-95rc.complete
GGAAGTAAAAGTCGTAACAAGGTTTCCGTAGGTGAACCTGCGGAAGGATCATTGTCGTGA
CCCTGACCAAAATAGACCGTGCACGCGTCATCCAGCCTGCCGGCGGCGGCACCGTTCGTC
GCTCGGCCAAGTCCTCGACAGCCTCCTCTCCTCGGAGTGGGGGCTCGGGGTAAAAGAACC
CACGACGCCTAAGGCGTCAAGGAACACTGTGCCTAGCCCGGGGACGCGGACGGCTTGCTG
GCCGCCCCCCGTGCTGCAATGCTATTTAATCCACACGACTCTCGGCAACGGATATCTCGG
CTCTCGCATCGATGAAGAACGTAGCGAAATGCGATACCTGGTGTGAATTGCAGAATCCCG
CGAACCATCGAGTCTTTGAACGCAAGTTGCGCCCGAGGCCATTCGGCTGAGGGCACGCCT
GCCTGGGCGTCACGCAAAACACGCTCCCACACCCTCATCGGGTAGCGGGACGCGGCATGT
GGTCCCCCGTAGCGCAAGCTGCGGTGGGCCGAAGATATGGCTGCCGGCGTATCGTGCCGG
ACACAGCGCGTGGTGGGCGACCTCGCTCTACTTACCGCAGTGCATCCGGGGCGTAGCCGA
CGTGATGGCCTCTAAGGACCCTATGAACGGTGCGCACGACG
>ITS.15_C1_ITS.3-MLZ_7561 E04.012-F04_011.complete
GGAAGTAAAAGTCGTAACAAGGTTTCCGTAGGTGAACCTGCGGAAGGATCATTGTCGTGA
CCCTGACCAAAATAGACCGTGCACGCGTCATCCAGCCTGCCGGCGGCGGCACCGTTCGTC
GCTCGGCCAAGTCCTCGACAACCTCCTCTCCACGGAGTGGGGGCTCGGGGTAAAAGAACC
CACGACGCCTAAGGCGTCAAGGAACACTGTGCCTAGCCCGGGGACGCGGACGGCTTGCTG
GCCGCCCCCCGTGCTGCAATGCTATTTAATCCACACGACTCTCGGCAACGGATATCTCGG
CTCTCGCATCGATGAAGAACGTAGCGAAATGCGATACCTGGTGTGAATTGCAGAATCCCG
CGAACCATCGAGTCTTTGAACGCAAGTTGCGCCCGAGGCCATTCGGCTGAGGGCACGCCT
GCCTGGGCGTCACGCAAAACACGCTCCCACACCCTCATCGGGTAGCGGGACGCGGCATGT
GGTCCCCCGTAGCGCAAGCTGCGGTGGGCCGAAGATATGGCTGCCGGCGTATCGTGCCGG
ACACAGCGCGTGGTGGGCGACCTCGCTTTACTTACCGCAGTGCATCCGGGGCGTAGCCGA
CGTGATGGCCTCTAAGGACCCTATGAACGGTGCGCACGACG
>ITS.15_C2_ITS.4-MLZ_7561rc G04.010-H04.009.complete
GGAAGTAAAAGTCGTAACAAGGTTTCCGTAGGTGAACCTGCGGAAGGATCATTGTCGTGA
CCCTGACCAAAATAGACCGTGCACGCGTCATCCAGCCTGCCGGCGGCGGCACCGTTCGTC
GCTCGGCCAAGTCCTCGACAACCTCCTCTCCTCGGAGTGGGGGCTCGGGGTAAAAGAACC
CACGACGCCTAAGGCGTCAAGGAACACTGTGCCTAGCCCGGGGACGCGGACGGCTTGCTG
GCCGCCCCCCGTGCTGCAATGCTATTTAATCCACACGACTCTCGGCAACGGATATCTCGG
CTCTCGCATCGATGAAGAACGTAGCGAAATGCGATACCTGGTGTGAATTGCAGAATCCCG
CGAACCATCGAGTCTTTGAACGCAAGTTGCGCCCGAGGCCATTCGGTTGAGGGCACGCCC
GCCTGGGCGTCACGCAAAACACGCTCCCACACCCTCATCGGGTAGCGGGACGCGGCATGT
GGTCCCCCGTAGCGCAAGCTGCGGTGGGCCGAAGATATGGCTGCCGGCGTATCGTGCCGG
ACACAGCGCGTGGTGGGCGGCCTCGCTTTACTTACCGCAGTGCATCCGGGGCGTAGCCGA
CGTGATGGCCTCTAAGGACCCTTTGAACGGTGCGCACGACG
>ITS.49_6_2_C2_ITS.2-MLZ_7561 C04.014-D04.013. complete
GGAAGTAAAAGTCGTAACAAGGTTTCCGTAGGTGAACCTGCGGAAGGATCATTGTCGTGA
CCCTGACCAAAATAGACCGTGCACGCGTCATCCAGCCTGCCGGCGGCGGCACCGTTCGTC
GCTCGGCCAAGTCCTCGACAACCTCCTCTCCTCGGAGTGGGGGCTCGGGGTAAAAGAACC
CACGACGCCTAAGGCGTCAAGGAACACTGTGCCTAGCCCGGGGACGCGGACGGCTTGCTG
GCCGCCCCCCGTGCTGCAATGCTATTTAATCCACACGACTCTCGGCAACGGATATCTCGG

CTCTCGCATCGATGAAGAACGTAGCGAAATGCGATACCTGGTGTGAATTGCAGAATCCCG
CGAACCATCGAGTCTTTGAACGCAAGTTGCGCCCGAGGCCATTCGGCTGAGGGCACGACT
GCCTGGGCGTCACGCAAAACACGCTCCCACCCCCTCATCGGGTAGCGGGACGCGGCATGT
GGCCCCCCGTAGCGCAAGCTGCGGTGGGCCGAAGATATGGCTGCCGGCGTATCGTGCCGG
ACACAGCGCGTGGTGGGCGACCTCGCTTTACTTACCGCAGTGCATCCGGGGCGTAGCCGA
CGTGATGGCCTCTAAGGACCCTATGAACGGTGCGCACGACG
>ITS.49_6_2_ITS.1-MLZ_7561 B04.015-A04.016 complete
GGAAGTAAAAGTCGTAACAAGGTTTCCGTAGGTGAACCTGCGGAAGGATCATTGTCGTGA
CCCTGACCAAAATAGACCGCGCACGCGTAATCCAGCCTGCCGGCGATGGCACCGTCCGTC
GCTCGGCCAAGTCCTCGACAACCTCATCTCCTCGGAGTGGGGCTCGGGGTAAAAGAACCC
ACGACGCCTAAGGCGTCAAGGAACACTGTGCCTAGCCCGGGGACGCGGACGGCTTGCTGG
CCGCCCCCCGTGCTGCAATGCTATTTAATCCGCACGACTCTCGGCAACGGATATCTCGGC
TCTCGCATCGATGAAGAACGTAGCGAAATGCGATACCTGGTGTGAATTGCAGAATCCCGC
GAACCATCGAGTCTTTGAACGCAAGTTGCGCCCGAGGCCATTCGGCTGAGGGCACGCCTG
CCTGGGCGTCACGCAAAACACGCTCCCACCCCCTCATCGGGTAGCGGGACGCGGCATGTG
GCCCCCCGTAGCGCAAGCTGCGGTGGGCCGAAGATATGGCTGCCGGCGTGTCGTGCCGGA
CACAGCGCGTGGTGAGCGACCTCGCTTTACTTACCGCAGTGCATCCGGGGCGTAGCCGAC
GTGATGGCCTCCAAGGACCCTATGAACGGTGCGCACGACG
>ITS.12_6_2_C1
GGAAGTAAAAGTCGTAACAAGGTTTCCGTAGGTGAACCTGCGGAAGGATCATTGTCGTGA
CCCTGACCAAAATAGACCGTGCACGCGTCATCCAGCCTGCCGGCGGCGGCACCGTTCGTC
GCTCGGCCAAGTCCTCGACAACCTCCTCTCCTCGGAGTGGGGGCTCGGGGTAAAAGAACC
CACGACGCCTAAGGCGTCAAGGAACACTGTGCCTAGCCCGGGGACGCGGACGGCTTGCTG
GCCGCCCCCCGTGCTGCAATGCTATTTAATCCACACGACTCTCGGCAACGGATATCTCGG
CTCTCGCATCGATGAAGAACGTAGCGAAATGCGATACCTGGTGTGAATTGCAGAATCCCG
CGAACCATCGAGTCTTTGAACGCAAGTTGCGCCCGAGGCCATTCGGCTGAGGGCACGCCT
GCCTGGGCGTCACGCAAAACACGCTCCCACACCCTCATCGGGTAGCGGGACGCGGCATGT
GGTCCCCCGTAGCGCAAGCTGCGGTGGGCCGAAGATATGGCTGCCGGCGTATCGTGCCGG
ACACAGCGCGTGGTGGGCGACCTCGCTTTACTTACCGCAGTGCATCCGGGGCGTAGCCAA
CGTGATGGCCTCTAAGGACCCTATGAACGGTGCGCACGACG
>ITS.12_6_2_C2
GGAAGTAAAAGTCGTAACAAGGTTTCCGTAGGTGAACCTGCGGAAGGATCATTGTCGTGA
CCCTGACCAAAATAGACCGTGCACGCGTCATCCAGCCTGCCGGCGGCGGCACCGTTCGTC
GCTCGGCCAAGTCCTCGACAACCTCCTCTCCTCGGAGTGGGGGCTCGGGGTAAAAGAACC
CACGACGCCTAAGGCGTCAAGGAACACTGTGCCTAGCCCGGGGACGCGGACGGCTTGCTG
GCCGCCCCTCGTGCTGCAATGCTATTTAATCCACACGACTCTCGGCAACGGATATCTCGG
CTCTCGCATCGATGAAGAACGTAGCGAAATGCGATACCTGGTGTGAATTGCAGAATCCCG
CGAACCATCGAGTCTTTGAACGCAAGTTGCGCCCGAGGCCATTCGGCTGAGGGCACGCCT
GCCTGGGCGTCACGTAAAACACGCTCCCACACCCTCATCGGGTAGCGGGACGCGGCATGT
GGTCCCCCGTAGCGCAAGCTGCGGTGGGCCGAAGATATGGCTGCCGGCGTATCGTGCCGG
ACACAGCGCGTGGTGGGCGACCTCGCTTTACTTACCGCAGTGCATCCGGGGCGTAGCCGA
CGTGATGGCCTCTAAGGACCCTATGAACGGTGCGCACGACG
>ITS.12_6_2_C3
GGAAGTAAAAGTCGTAACAAGGTTTCCGTAGGTGAACCTGCGGAAGGATCATTGTCGTGA
CCCTGACCAAAATAGACCGTGCACGCGTCATCCAGCCTGCCGGCGGCGGCACCGTTCGTC
GCTCGGCCAAGTCCTCGACAACCTCCTCTCCTCGGAGTGGGGGCTCGGGGTAAAAGAACC
CACGACGCCTAAGGCGTCAAGGAACACTGTGCCTAGCCCGGGGACGCGAACGGCTTGCTG
GCCGCCCCCCGTGCTGCAATGCTATTTAATCCACACGACTCTCGGCAACGGATATCTCGG
CTCTCGCATCGATGAAGAACGTAGCGAAATGCGATACCTGGTGTGAATTGCAGAATCCCG
CGAACCATCGAGTCTTTGAACGCAAGTTGCGCCCGAGGCCATTCGGCTGAGGGCACGCCT
GCCTGGGCGTCACGCAAAACACGCTCCCACACCCTCATCGGGTAGCGGGACGCGGCATGT
GGTCCCCCGTAGCGCAAGCTGCGGTGGGCCGAAGATATGGCTGCCGGCGTATCGTGCCGG
ACACAGCGCGTGGTGGGCGACCTCGCCTTACTTACCGCAGTGCATCCGGGGCGTAGCCGA
CGTGATGGCCTCTAAGGACCCTTTGAACGGTGCGCACGACG

>ITS.12_6_2_C4
GGAAGTAAAAGTCGTAACAAGGTTTCCGTAGGTGAACCTGCGGAAGGATCATTGTCGTGA
CCCTGACCAAAATAGACCGTGCACGCGTCATCCAGCCTGCCGGCGGCGGCACCGTTCGTC
GCTCGGCCAAGTCCTCGACAACCTCCTCTCCTCGGAGTGGGGGCTCGGGGTAAAAGAACC
CACGACGCCTAAGGCGTCAAGGAACACTGTGCCTAGCCCGGGGACGCGGACGGCTTGCTG
GCCGCCCCCCGTGCTGCAATGCTATTTAATCCACACGACTCTCGGCAACGGATATCTCGG
CTCTCGCATCGATGAAGAACGTAGCGAAATGCGATACCTGGTGTGAATTGCAGAATCCCG
CGAACCATCGAGTCTTTGAACGCAAGTTGCGCCCGAGGCCACTCGGCTGAGGGCACGCCT
GCCTGGGCGTCACGCAAAACACGCTCCCACACCCTCATCGGGTAGCGGGACGCGGCATGT
GGTCCCCCGTAGCGCAAGCTGCGGTGGGCCGAAGATATGGCTGCCGGCGTATCGTGCCGG
ACACAGCGCGTGGTGGGCGACCTCGCTTTACTTACCGCAGTGCATCCGGGGCGTAGCCGA
CGTGATGGCCTCTAAGGACCCTATGAACGGTGCGCACGACG
>ITS.12_6_2_C5
GGAAGTAAAAGTCGTAACAAGGTTTCCGTAGGTGAACCTGCGGAAGGATCATTGTCGTGA
CCCTGACCAAAATAGACCGTGCACGCGTCATCCAGCCTGCCGGCGGCGGCACCGCTCGTC
GCTCGGCCAAGTCCTCGACAACCTCCTCTCCTCGGAGTGGGGGCTCGGGGTAAAAGAACC
CACGACGCCTAAGGCGTCAAGGAACACTGTGCCTAGCCCGGGGACGCGGACGGCTTGCTG
GCCGCCCCTCGTGCTGCAAGGCTATTTAATCCACACGACTCTCGGCAACGGATATCTCGG
CTCTCGCATCGATGAAGAACGTAGCGAAATGCGATACCTGGTGTGAATTGCAGAATCCCG
CGAACCATCGAGTCTTTGAACGCAAGGTGCGCCCGAGGCCATTCGGCTGAGGGCACGCCT
GCCTGGGCGTCACGTAAAACACGCTCCCACACCCTCATCGGGTAGCGGGACGCGGCATGT
GGTCCCCCGTAGCGCAAGCTGCGGTGGGCCGAAGATATGGCTGCCGGCGTATCGTGCCGG
ACACAGCGCGTGGTGGGCGACCTCGCTTTACTTACCGCAGTGCATCCGGGGCGTAGCCGA
CGTGATGGCCTCTAAGGACCCTTTGAACGGTGCGCACGACG
>ITS.12_6_2_C6
GGAAGTAAAAGTCGTAACAAGGTTTCCGTAGGTGAACCTGCGGAAGGATCATTGTCATGA
CCCTGACCAAAATAGACCGTGCACGCGTCATCCAGCCTGCCGGCGGCGGCACCGTTCGTC
GCTCGGCCAAGTCCTCGACAACCTCCTCTCCTCGGAGTGGGGGCTCGGGGTAAAAGAACC
CACGACGCCTAAGGCGTCAAGGAACACTGTGCCTAGCCCGGGGACGCGGACGGCTTGCTG
GCCGCCCCCCGTGCTGCAATGCTATTTAATCCACACGACTCTCGGCAACGGATATCTCGG
CTCTCGCATCGATGAAGAACGTAGCGAAATGCGATACCTGGTGTGAATTGCAGAATCCCG
CGAACCATCGAGTCTTTGAACGCAAGTTGCGCCCGAGGCCATTCGGCTGAGGGCACGCCT
GCCTGGGCGTCACGCAAAACACGCTCCCACACCCTCATCGGGTAGCGGGACGCGGCATGT
GGTCCCCCGTAGCGCAAGCTGCGGTGGGCCGAAGATATGGCTGCCGGCGTATCGTGCCGG
ACACAGCGCGTGGTGGGCGACCTCGCTTTACTTACCGCAGTGCATCCGGGGCGTAGCCGA
CGTGATGGCCTCTAAGGACCCTATGAACGGTGCGCACGACG
>ITS.12_6_2_C7
GGAAGTAAAAGTCGTAACAAGGTTTCCGTAGGTGAACCTGCGGAAGGATCATTGTCGTGA
CCCTGACCAAAATAGACCGTGCACGCGTCATCCAGCCTGCCGGCGGCGGCACCGTTCGTC
GCTCGGCCAAGTCCTCGACAACCTCCTCTCCTCGGAGTGGGGGCTCGGGGTAAAAGAACC
CACGACGCCTAAGGCGTCAAGGAACACTGTGCCTAGCCCGGGGACGCGGACGGCTTGCTG
GCCGCCCCCCGTGCTGCAATGCTATTTAATCCACACGACTCTCGGCAACGGATATCTCGG
CTCTCGCATCGATGAAGAACGTAGCGAAATGCGATACCTGGTGTGAATTGCAGAATCCCG
CGAACCATCGAGTCTTTGAACGCAAGTTGCGCCCGAGGCCATTCGGCTGAGGGCACGCCT
GCCTGGGCGTCACGCAAAACACGCTCCCACACCCTCATCGGGTAGCGGGACGCGGCATGT
GGTCCCCCGTAGCGCAAGCTGCGGTGGGCCGAAGATATGGCTGCCGGCTTATCGTGCCGG
ACACAGCGCGTGGTGGGCGACCTCGCTTTACTTACCGCAGTGCATCCGGGGCGTAGCCGA
CGTGATGGCCTCTAAGGACCCTTTGAACGGTGCGCACGACG
>ITS.12_6_2_C8
GGAAGTAAAAGTCGTAACAAGGTTTCCGTAGGTGAACCTGCGGAAGGATCATTGTCGTGA
CCCTGACCAAAATAGACCGTGCACGCGTCATCCAGCCTGCCGGCGGCGGCACCGTTCGTC
GCTCGGCCAAGTCCTCGACAACCTCCTCTCCTCGGAGTGGGGGCTCGGGGTAAAAGAACC
CACGACGCCTAAGGCGTCAAGGAACACTGTGCCTAGCCCGGGGACGCGGACGGCTTGCTG
GCCGCCCCCCGTGCTGCAATGCTATTTAATCCACACGACTCTCGGCAACGGATACCTCGG

CTCTCGCATCGATGAAGAACGTAGCGAAATGCGATACCTGGTGTGAATTGCAGAATCCCG
CGAACCATCGAGTCTTTGAACGCAAGTTGCGCCCGAGGCCATTCGGCTGAGGGCACGCCT
GCCTGGGCGTCACGCAAAACACGCTCCCACACCCTCATCGGGTAGAGGGACGCGGCATGT
GGTCCCCCGTAGCGCAAGCTGCGGTGGGCCGAAGATATGGCTGCCGGCGTATCGTGCCGG
ACACAGCGCGTGGTGGGCGACCTCGCTTTACTTACCGCAGTGCATCCGGGGCGTAGCCAA
CGTGATGGCCTCTAAGGACCCTATGAACGGTGCGCACGACG
>ITS.12_8_2_C1
GGAAGTAAAAGTCGTAACAAGGTTTCCGTAGGTGAACCTGCGGAAGGATCATTGTCGTGA
CCCTGACCAAAATAGACCGTGCACGCGTCATCCAGCCTGCCGGCGGCGGCACCGTTCGTC
GCTCGGCCAAGTCCTCGACAACCTCCTCTCCTCGGAGTGGGGGCTCGGGGTAAAAGAACC
CACGACGCCTAAGGCGTCAAGGAACACTGTGCCTAGCCCGGGGACGCGGACGGCTTGCTG
GCCGCCCCCCCGTGCTGCAATGCTATTTAATCCACACGACTCTCGGCAACGGATATCTCG
GCTCTCGCATCGATGAAGAACGTAGCGAAATGCGATACCTGGTGTGAATTGCAGAATCCC
GCGAACCATCGAGTCTTTGAACGCAAGTTGCGCCCGAGGCCATTCGGCTGAGGGCACGCC
TGCCTGGGCGTCACGCAAAACACGCTCCCACACCCTCATCGGGTAGCGGGACGCGGCATG
TGGTCCCCCGTAGCGCAAGCTGCGGTGGGCCGAAGATATGGCTGCCGGCGTATCGTGCCG
GACACAGCGCGTGGTGGGCGACCTCGCTTTACTTACCGCAGTGCATCCGGGGCGTAGCCG
ACGTGATGGCCTCTAAGGACCCTATGAACGGTGCGCACGACG
>ITS.12_8_2_C2
GGAAGTAAAAGTCGTAACAAGGTTTCCGTAGGTGAACCTGCGGAAGGATCATTGTCGTGA
CCCTGACCAAAATAGACCGTGCACGCGTCATCCAGCCTGCCGGCGGCGGCACCGTTCGTC
GCTCGGCCAAGTCCTCGACAACCTCCTCTCCTCGGAGTGGGGGCTCGGGGTAAAAGAACC
CACGACGCCTAAGGCGTCAAGGAACACTGTGCCTAGCCCGGGGACGCGGACGGCTTGCTG
GCCGCCCCCCCGTGCTGCAATGCTATTTAATCCACACGACTCTCGGCAACGGATATCTCGG
CTCTCGCATCGATGAAGAACGTAGCGAAATGCGATACCTGGTGTGAATTGCAGAATCCCG
CGAACCATCGAGTCTTTGAACGCAAGTTGCGCCCGAGGCCATTCGGCTGAGGGCACGCCT
GCCTGGGCGTCACGCAAAACACGCTCCCACACCCTCATCGGGTAGCGGGACGCGGCATGT
GGTCCCCCGTAGCGCAAGCTGCGGTGGGCCGAAGATATGGCTGCCGGCGTATCGTGCCGG
ACACAGCGCGTGGTGGGCGACCTCGCTTTACTTACCGCAGTGCATCCGGGGCGTAGCCGA
CGTGATGGCCTCTAAGGACCCTTTGAACGGTGCGCACGACG
>ITS.12_8_2_C3
GGAAGTAAAAGTCGTAACAAGGTTTCCGTAGGTGAACCTGCGGAAGGATCATTGTCGTGA
CCCTGACCAAAATAGACCGTGCACGCGTCATCCAGCCTGCCGGCGGCGGCACCGTTCGTC
GCTCGGCCAAGTCCTCGACAACCTCCTCTCCTCGGAGTGGGGGCCCGGGGTAAAAGAACC
CACGACGCCTAAGGCGTCAAGGAACACTGTGCCTAGCCCGGGGACGCGGACGGCTTGCTG
GCCGCCCCCCCGTGCTGCAATGCTATTTAATCCACACGACTCTCGGCAACGGATATCTCGG
CTCTCGCATCGATGAAGAACGTAGCGAAATGCGATACCTGGTGTGAATTGCAGAATCCCG
CGAACCATCGAGTCTTTGAACGCAAGTTGCGCCCGAGGCCATTCGGCTGAGGGCACGCCT
GCCTGGGCGTCACGCAAAACACGCTCCCACACCCTCATCGGGTAGCGGGACGCGGCATGT
GGTCCCCCGTAGCGCAAGCTGCGGTGGGCCGAAGATATGGCTGCCGGCGTATCGTGCCGG
ACACAGCGCGTGGTGGGCGACCTCGCTTTACTTACCGCAGTGCATCCGGGGCGTAGCCGA
CGTGATGGCCTCTAAGGACCCTATGAACGGTGCGCACGACG
>ITS.12_8_2_C4
GGAAGTAAAAGTCGTAACAAGGTTTCCGTAGGTGAACCTGCGGAAGGATCATTGTCGTGA
CCCTGACCAAAATAGACCGTGCACGCGTCATCCAGCCTGCCGGCGGCGGCACCGTTCGTC
GCTCGGCCAAGTCCTCGACAACCTCCTCTCCTCGGAGTGGGGGCTCGGGGTAAAAGAACC
CACGACGCCTAAGGCGTCAAGGAACACTGTGCCTAGCCCGGGGACGCGGACGGCTTGCTG
GCCGCCCCCCCGTGCTGCAATGCTATTTAATCCACACGACTCTCGGCAACGGATATCTCGG
CTCTCGCATCGATGAAGAACGTAGCGAAATGCGATACCTGGTGTGAACTGCAGAATCCCG
CGAACCATCGAGTCTTTGAACGCAAGTTGCGCCCGAGGCCATTCGGCTGAGGGCACGCCT
GCCTGGGCGTCACGCAAAACACGCTCCCACACCCTCATCGGGTAGCGGGACGCGGCTTGT
GGTTCCCCGTAGCGCAAGCTGCGGTGGGCCGAAGATATGGCTGCCGGCGTATCGTGCCGG
ACACAGCGCGTGGTGGGCGACCTCGCTTTACTTACCGCAGTGCATCCGGGGCGTAGCCGA
CATGATGGCCTCTAAGGACCCTTTGAACGGTGCGCACGACG

>ITS.12_8_2_C5
GGAAGTAAAAGTCGTAACAAGGTTTCCGTAGGTGAACCTGCGGAAGGATCATTGTCGTGA
CCCTGACCAAAATAGACCGTGCACGCGTCATCCAGCCTGCCGGCGGCGGCACCGTTCGTC
GCTCGGCCAAGTCCTCGACAACCTCCTCTCCTCGGAGTGGGGGCTCGGGGTAAAAGAACC
CACGACGCCTAAGGCGTCAAGGAACACTGTGCCTAGCCCGGGGACGCGGACGGCTTGCTG
GCCGCCCCTCGTGCTGCAATGCTATTTAATCCACACGACTCTCGGCAACGGATATCTCGG
CTCTCGCATCGATGAAGAACGTAGCGAAATGCGATACCTGGTGTGAATTGCAGAATCCCG
CGAACCATCGAGTCTTTGAACGCAAGTTGCGCCCGAGGCCATTCGGCTGAGGGCACGCCT
GCCTGGGCGTCACGCAAAACACGCTCCCACACCCTCATCGGGTAGCGGGACGCGGCATGT
GGTCCCCCGTAGCGCAAGCTGCGGTGGGCCGAAGATATGGCTGCCGGCGTATCGTGCCGG
ACACAGCGCGTGGTGGGCGACCTCGCTTTACTTACCGCAGTGCATCCGGGGCGTAGCCGA
CGTGATGGCCTCTAAGGACCCTATGAACGGTGCGCACGACG
>ITS.12_8_2_C8
GGAAGTAAAAGTCGTAACAAGGTTTCCGTAGGTGAACCTGCGGAAGGATCATTGTCGTGA
CCCTGACCAAAATAGACCGTGCACGCGTCATCCAGCCTGCCGGCGGCGGCACCGTTCGTC
GCTCGGCCAAGTCCTCGACAACCTCCTCTCCTCGGAGTGGGGGCTCGGGGTAAAAGAACC
CACGACGCCTAAGGCGTCAAGGAACACTGTGCCTAGCCCGGGGACGCGGACGGCTTGCTG
GCCGCCCCCCGTGCTGCAATGCTATTTAATCCACACGACTCTCGGCAACGGATATCTCGG
CTCTCGCATCGATGAAGAACGTAGCGAAATGCGATACCTGGTGTGAATTGCAGAATCCCG
CGAACCATCGAGTCTTTGAACGCAAGTTGCGCCCGAGGCCATTCGGCTGAGGGCACGCCT
GCCTGGGCGTCACGCAAAACACGCTCCCACACCCTCATCGGGTAGCGGGACGCGGCATGT
GGTCCCCCGTAGCGCAAGCTGCGGTGGGCCGAAGATATGGCTGCCGGCGTATCGTGCCGG
ACACAGCGCGTGGTGGGCGACCTCGCTTTACTTACCGCAGTGCATCCGGGGCGTAGCCGA
CGTGATGGCCTCTAAGGACCCTATGAACGGTGCGCACGACG
>ITS.12_8_2_C9
GGAAGTAAAAGTCGTAACAAGGTTTCCGTAGGTGAACCTGCGGAAGGATCATTGTCGTGA
CCCTGACCAAAATAGACCGTGCACGCGTCATCCAGCCTGCCGGCGACGGCACCGTTCGTC
GCTCGGCCAAGTCCTCGACAACCTCCTCTCCTCGGAGTGGGGGCTCGGGGTAAAAGAACC
CACGACGCCTAAGGCGTCAAGGAACACTGTGCCTAGCCCGGGGACGCGGACGGCTTGCTG
GCCGCCCCCCGTGCTGCAATGCTATTTAATCCACACGGCTCTCGGCAACGGATATCTCGG
CTCTCGCATCGATGAAGAACGTAGCGAAATGCGATACCTGGTGTGAATTGCAGAATCCCG
CGAACCATCGAGTCTTTGAACGCAAGTTGCGCCCGAGGCCATTCGGCTGAGGGCACGCCT
GCCTGGGCGTCACGCAAAACACGCTCCCACACCCTCATCGGGTAGCGGGACGCGGCATGT
GGTCCCCCGTAGCGCAAGCTGCGGTGGGCCGAAGATATGGCTGCCGGCGTATCGTGCCGG
ACACAGCGCGTGGTGGGCGACCTCGCTTTACTTACCGCAGTGCATCCGGGGCGTAGCCGA
CGTGATGGCCTCTAAGGACCCTTTGAACGGTGCGCACGACG
>ITS.12_8_2_C10
GGAAGTAAAAGTCGTAACAAGGTTTCCGTAGGTGAACCTGCGGAAGGATCATTGTCGTGA
CCCTGACCAAAATAGACCGTGCACGCGTCATCCAGCCTGCCGGCGGCGGCACCGTTCGTC
GCTCGGCCAAGTCCTCGACAACCTCCTCTCCTCGGAGTGGGGGCTCGGGGTAAAAGAACC
CACGACGCCTAAGGCGTCAAGGAACACTGTGCCTAGCTCGGGGACGCGGACGGCTTGCTG
GCCGCCCCTCGTGCTGCAATGCTATTTAATCCACACGACTCTCGGCAACGGATATCTCGG
CTCTCGCATCGATGAAGAACGTAGCGAAATGCGATACCTGGTGTGAATTGCAGAATCCCG
CGAACCATCGAGTCTTTGAACGCAAGTTGCGCCCGAGGCCATTCGGCTGAGGGCACGCCT
GCCTGGGCGTCACGTAAAACACGCTCCCACACCCTCATCGGGTAGCGGGACGCGGCATGT
GGTCCCCCGTAGCGCAAGCTGCGGTGGGCCGAAGATATGGCTGCCGGCGTATCGTGCCGG
ACACAGCGCGTGGTGGGCGACCTCGCTTTACTTACCGTAGTGCATCCGGGGCGTAGCCGA
CGTGATGGCCTCTAAGGACCCTTTGAACGGTGCGCACGACG
>ITS.13_3_C1Frc
GGAAGTAAAAGTCGTAACAAGGTTTCCGTAGGTGAACCTGCGGAAGGATCATTGTCGTGA
CCCTGACCAAAATAGACCGTGCACGCGTCATCCAGCCCGCCGGCGGCGGCACCGTTCGTC
GCTCGGCCAAGTCCTCGACAACCTCCTCTCCTCGGAGTGGGGGCTCGGGGTAAAAGAACC
CACGACGCCTAAGGCGTCAAGGAACACTGTGCCTAGCCCGGGGACGCGGACGGCTTGCTG
GCCGCCCCCCGTGCTGCAATGCTATTTAATCCACACGACTCTCGGCAACGGATATCTCGG

CTCTCGCATCGATGAAGAACGTAGCGAAATGCGATACCTGGTGTGAATTGCCGAATCCCG
CGAACCATCGAGTCTTTGAACGCAAGTTGCGCCCGAGGCCATTCGGCTGAGGGCACGCCT
GCCTGGGCGTCACGCAAAACACGCTCCCACACCCTCATCGGGTAGCGGGACGCGGCATGT
GGTCCCCCGTAGCGCAAGCTGCGGTGGGCCGAAGATATGGCTGCCGGCGTATCGTGCCGG
ACACAGCGCGTGGTGGGCGACCTCGCTTTACTTACCGCAGTGCATCCGGGGCGTAGCCGA
CGTGATGGCCTCTAAGGACCCTATGAACGGTGCGCACGACG
>ITS.13_3_C2
GGAAGTAAAAGTCGTAACAAGGTTTCCGTAGGTGAACCTGCGGAAGGATCATTGTCGTGA
CCCTGACCAAAATAGACCGTGCACGCGTCATCCACCCTGCCGGCGGCGGCACCGTTCGTC
GCTCGGCCAAGTCCTCGACAACCTCCTCTCCTCGGAGTGGGGGCTCGGGGTAAAAGAACC
CACGACGCCTAAGGCGTCAAGGAACACTGTGCCTAGCCCGGGGACGCGGACGGCTTGCTG
GCCGCCCCCCGTGCTGCAATGCTATTTAATCCACACGACTCTCGGCAACGGATATCTCGG
CTCTCGCATCGATGAAGAACGTAGCGAAATGCGATACCTGGTGTGAATTGCAGAATCCCG
CGAACCATCGAGTCTTTGAACGCAAGTTGCGCCCGAGGCCATTCGGCTGAGGGCACGCCT
GCCTGGGCGTCACGCAAAACACGCTCCCACACCCTCATCGGGTAGCGGGACGCGGCATGT
GGTCCCCCGTAGCGCAAGCTGCGGTGGGCCGAAGATATGGCTGCCGGCGTATCGTGCCGG
ACACAGCGCGTGGTGGGCGACCTCGCTTTACTTACCGCAGTGCATCCGGGGCGTAGCCGA
CGTGATGGCCTCTAAGGACCCTATGAACGGTGCGCACGACG
>ITS.13_3_C3F
GGAAGTAAAAGTCGTAACAAGGTTTCCGTAGGTGAACCTGCGGAAGGATCATTGTCGTGA
CCCTGACCAAAATAGACCGTGCACGCGTCATCCAGCCTGCCGGCGGCGGCACCGTTCGTC
GCTCGGCCAAGTCCTCGACAACCTCCTCTCCTCGGAGTGGGGGCTCGGGGTAAAAGAACC
CACGACGCCTAAGGCGTCAAGGAACACTGTGCCTAGCCCGGGGACGCGGACGGCTTGCTG
GCCGCCCCTCGTGCTGCAATGCTATTTAATCCACACGACTCTCGGCAACGGATATCTCGG
CTCTCGCATCGATGAAGAACGTAGCGAAGTGCGATACCTGGTGTGAATTGCAGAATCCCG
CGAACCATCGAGTCTTTGAACGCAAGTTGCGCCCGAGGCCATTCGGCTGAGGGCACGCCT
GCCTGGGCGTCACGCAAAACACGCTCCCACACCCTCATCGGGTAGCGGGACGCGGCATGT
GGTCCCCCGTAGCGCAAGCTGCGGTGGGCCGAAGATATGGCTGCCGGCGTATCGTGCCGG
ACACAGCGCGTGGTGGGCAACCTCGCTTTACTTACCGCAGTGCATCCGGGGCGTAGCCGA
CGTGATGGCCTCTAAGGACCCTATGAACGGTGCGCACGACG
>ITS.13_3_C6Frc
GGAAGTAAAAGTCGTAACAAGGTTTCCGTAGGTGAACCTGCGGAAGGATCATTGTCGTGA
CCCTGACCAAAATAGACCGTGCACGCGTCATCCAGCCTGCCGGCGGCGGCACCGTTCGTC
GCTCGGCCAAGTCCTCGACAACCTCCTCTCCTCGGAGTGGGGGCTCGGGGTAAAAGAACC
CACGACGCCTAAGGCGTCAAGGAACACTGTGCCTAGCCCGGGGACGCGGACGGCTTGCTG
GCCGCCCCCCGTGCTGCAATGCTATTTAATCCACACGACTCTCGGCAACGGATATCTCGG
CTCTCGCATCGATGAAGAACGTAGCGAAATGCGATACCTGGTGTGAATTGCAGAATCCCG
CGAACCATCGAGTCTTTGAACGCAAGTTGCGCCCGAGGCCATTCGGTTGAGGGCACGCCT
GCCTGGGCGTCACGCAAAACACGCTCCCACACCCTCATCGGGTAGCGGGACGCGGCATGT
GGTCCCCCGTAGCGCAAGCTGCGGTGGGCCGAAGATATGGCTGCCGGCGTATCGTGCCGG
ACACAGCGCGTGGTGGGCGACCTCGCTTTACTTACCGCAGTGCATCCGGGGCGTAGCCGA
CGTGATGGCCTCTAAGGACCCTATGAACGGTGCGCACGACG
>ITS.13_3_C7Frc
GGAAGTAAAAGTCGTAACAAGGTTTCCGTAGGTGAACCTGCGGAAGGATCATTGTCGTGA
CCCTGACCAAAATAGACCGTGCACGCGTCATCCAGCCTGCCGGCGGCGGCACCGTTCGTC
GCTCGGCCAAGTCCTCGACAACCTCCTCTCCTCGGAGTGGGGGCTCGGGGTAAAAGAACC
CACGACGCCTAAGGCGTCAAGGAACACTGTGCCTAGCCCGGGGACGCGGACGGCTTGCTG
GCCGCCCCTCGTGCTGCAATGCTATTTAATCCACACGACTCTCGGCAACGGATATCTCGG
CTCTCGCATCGATGAAGAACGTAGCGAAATGCGATACCTGGTGTGAATTGCAGAATCCCG
CGAACCATCGAGTCTTTGAACGCAAGTTGCGCCCGAGGCCATTCGGCTGAGGGCACGCCT
GCCTGGGCGTCACGCAAAACACGCTCCCACACCCTCATCGGGTAGCGGGACGCGGCATGT
GGTCCCCCGTAGCGCAAGCTGCGGTGGGCCGAAGATATGGCTGCCGGCGTATCGTGCCGG
ACACAGCGCGTGGTGGGCGACCTCGCTTTACTTACCGCAGTGCATCCGGGGCGTAGCCGA
CGTGATGGCCTCTAAGGACCCTTTGAACGGTGCGCACGACG

>ITS.13_3_C8Frc
GGAAGTAAAAGTCGTAACAAGGTTTCCGTAGGTGAACCTGCGGAAGGATCATTGTCGTGA
CCCTGACCAAAATAGACCGTGCACGCGTCATCCAGCTTGCCGGCGGCGGCACCGTTCGTC
GCTCGGCCAAGTCCTCGACAACCTCCTCTCCTCGGAGTGGGGGCTCGGGGTAAAAGAACC
CACGACGCCTAAGGCGTCAAGGAACACTGTGCCTAGCTCGGGGACGCGGACGGCTTGCTG
GCCGCCCCTCGTGCTGCAATGCTATTTAATCCACACGACTCTCGGCAACGGATATCTCGG
CTCTCGCATTGATGAAGAACGTAGCGAAATGCGATACCTGGTGTGAATTGCAGAATCCCG
CGAACCATCGAGTCTTTGAACGCAAGTTGCGCCCGAGGCCATTCGGCTGAGGGCACGCCT
GCCTGGGCGTCACGCAAAACACGCTCCCACACCCTCATCGGGTAGCGGGACGCGGCATGT
GGTCCCCCGTAGCGCAAGCTGCTGTGGGCCGAAGATATGGCTGCCGGCGTATCGTGCCGG
ACACAGCGCGTGGTGGGCGACCTCGCTTTACTTACCGCAGTGCATCCGGGGCGTAGCCGA
CGTGATGGCCTCTAAGGACCCTATGAACGGTGCGCACGACG
>ITS.13_3_C9
GGAAGTAAAAGTCGTAACAAGGTTTCCGTAGGTGAACCTGCGGAAGGATCATTGTCGTGA
CCCTGACCAAAATAGACCGAGCACGCGTCATCCAGCCTGCCGGCGGCGGCACCGTTCGTC
GCTCGGCCAAGTCCTCGACAACCTCCTCTCCTCGGAGTGGGGGCTCGGGGTAAAAGAACC
CACGACGCCTAAGGCGTCAAGGAACACTGTGCCTAGCCCGGGGACGCGGACGGCTTGCTG
GCCGCCCCCCGTGCTGCAATGCTATTTAATCCACACGACTCTCGGCAACGGATATCTCGG
CTCTCGCATCGATGAAGAACGTAGCGAAATGCGATACCTGGTGTGAATTGCAGAATCCCG
CGAACCATCGAGTCTTTGAACGCAAGTTGCGCCCGAGGCCATTCGGCTGAGGGCACGCCT
GCCTGGGCGTCACGCAAAACACGCTCCCACACCCTCATCGGGTAGCGGGACGCGGCATGT
GGTCCCCCGTAGCGCAAGCTGCGGTGGGCCGAAGATATGGCTGCCGGCGTATCGTGCCGG
ACACAGCGCGTGGTGGGCGACCTCGCTTTACTTACCGCAGTGCATCCGGGGCGTAGCCGA
CGTGATGGCCTCTAAGGACCCTATGAACGGTGCGCACGACG
>ITS.13_3_C10F
GGAAGTAAAAGTCGTAACAAGGTTTCCGTAGGTGAACCTGCGGAAGGATCATTGTCGTGA
CCCTGACCAAAATAGACCGTGCACGCGTCATCCACCCTGCCGGCGGCGGCACCGTTCGTC
GCTCGGCCAAGTCCTCGACAACCTCCTCTCCTCGGAGTGGGGGCTCGGGGTAAAAGAACC
CACGACGCCTAAGGCGTCAAGGAACACTGTGCCTAGCCCGGGGACGCGGACGGCTTGCTG
GCCGCCCCCCGTGCTGCAATGCTATTTAATCCACACGACTCTCGGCAACGGATATCTCGG
CTCTCGCATCGATGAAGAACGTAGCGAAATGCGATACCTGGTGTGAATTGCAGAATCCCG
CGAACCATCGAGTCTTTGAACGCAAGTTGCGCCCGAGGCCATTCGGCTGAGGGCACGCCT
GCCTGGGCGTCACGCAAAACACGCTCCCACACCCTCATCGGGTAGCGGGACGCGGCATGT
GGTCCCCCGTAGCGCAAGCTGCGGTGGGCCGAAGATATGGCTGCCGGCGTATCGTGCCGG
ACACAGCGCGTGGTGGGCGACCTCGCTTTACTTACCGCAGTGCATCCGGGGCGTAGCCGA
CGTGATGGCCTCTAAGGACCCTTTGAACGGTGCGCACGACG
>ITS.13_4_3_C1Frc
GGAAGTAAAAGTCGTAACAAGGTTTCCGTAGGTGAACCTGCGGAAGGATCATTGTCGTGA
CCCTGACCAAAATAGACCGTGCACGCGTCATCCAGCCTGCCGGCGGCGGCACCGTTCGTC
GCTCGGCCAAGTCCTCGACAACCTCCTCTCCTCGGAGTGGGGGCTCGGGGTAAAAGAACC
CACGACGCCTAAGGCGTCAAGGAACACTGTGCCTAGCCCGGGGACGCGGACGGCTTGCTG
GCCGCCCCCCGTGCTGCAATGCTATTTAATCCACACGACTCTCGGCAACGGATATCTCGG
CTCTCGCATCGATGAAGAACGTAGCGAAATGCGATACCTGGTGTGAATTGCAGAATCCCG
CGAACCATCGAGTCTTTGAACGCAAGTTGCGCCCGAGGCCATTCGGCTGAGGGCACGCCT
GCCTGGGCGTCACGCAAAACACGCTCCCACACCCTCATCGGGTAGCGGGACGCGGCATGT
GGTCCCCCGTAGCGCAAGCTGCGGTGGGCCGAAGATATGGCTGCCGGCGTATCGTGCCGG
ACACAGCGCGTGGTGGGCGACCTCGCTTTACTTACCGCAGTGCATCCGGGGCGTAGCCGA
CGTGATGGCCTCTAAGGACCCTTTGAACGGTGCGCACGACG
>ITS.13_4_3_C3F
GGAAGTAAAAGTCGTAACAAGGTTTCCGTAGGTGAACCTGCGGAAGGATCATTGTCGTGA
CCCTGACCAAAATAGACCGTGCACGCGTCATCCAGCCTGCCGGCGGCGGCACCGTTCGTC
GCTCGGCCAAGTCCTCGACAACCTCCTCTCCTCGGAGTGGGGGCTCGGGGTAAAAGAACC
CACGACGCCTAAGGCGTCAAGGAACACTGTGCCTAGCCCGGGGACGCGGACGGCTTGCTG
GCCGCCCCTCGTGCTGCAATGCTATTTAATCCACACGACTCTCGGCAACGGATATCTCGG

CTCTCGCATCGATGAAGAACGTAGCGAAATGCGATACCTGGTGTGAATTGCAGAATCCCG
CGAACCATCGAGTCTTTGAACGCAAGTTGCGCCCGAGGCCATTCGGCTGAGGGCACGCCT
GCCTGGGCGTCACGTAAAACACGCTCCCACACCCTCATCGGGTAGCGGGACGCGGCTTGT
GGTTCCCCGTAGCGCAAGCTGCGGTGGGCCGAAGATATGGCTGCCGGCGTATCGTGCCGG
ACACAGCGCGTGGTGGGCGACCTCGCTTTACTTACCGCAGTGCATCCGGGGCGTAGCCGA
CGTGATGGCCTCTAAGGACCCTTTGAACGGTGCGCACGACG
>ITS.13_4_3_C4Frc
GGAAGTAAAAGTCGTAACAAGGTTTCCGTAGGTGAACCTGCGGAAGGATCATTGTCGTGA
CCCTGACCAAAATAGACCGTGCACGCGTCATCCAGCCTGCCGGCGGCGGCATCGTTCGTC
GCTCGGCCAAGTCCTCGACAACCTCCTCTCCTCGGAGTGGGGGCTCGGGGTAAAAGAACC
CACGACGCCTAAGGCGTCAAGGAACACTGTGCCTAGCCCGGGGACGCGGACGGCTTGCTG
GCCGCCCCCCGTGCTGCAATGCTATTTAATCCACACGACTCTCGGCAACGGATATCTCGG
CTCTCGCATCGATGAAGAACGTAGCGAAATGCGATACCTGGTGTGAATTGCAGAATCCCG
CGAACCATCGAGTCTTTGAACGCAAGTTGCGCCCGAGGCCATTCGGCTGAGGGCACGCCT
GCCTGGGCGTCACGTAAAACACGCTCCCACACCCTCATCGGGTAGCGGGACGCGGCATGT
GGTCCCCCGTAGCGCAAGCTGCGGTGGGCCGAAGATATGGCTGCCGGCGTATCGTGCCGG
ACACAGCGCGTGGTGGGCGACCTCGCTTTACTTACCGCAGTGCATCCGGGGCGTAGCCGA
CGTGATGGCCTCTAAGGACCCTATGAACGGTGCGCACGACG
>ITS.13_4_3_C5F
GGAAGTAAAAGTCGTAACAAGGTTTCCGTAGGTGAGCCTGCGGAAGGATCATTGTCGTGA
CCCTGACCAAAATAGACCGTGCACGCGTCATCCAGCCTGCCGGCGGCGGCACCGTTCGTC
GCTCGGCCAAGTCCTCGACAACCTCCTCTCCTCGGAGTGGGGGCTCGGGGTAAAAGAACC
CACGACGCCTAAGGCGTCAAGGAACACTGTGCCTAGCCCGGGGACGCGGACGGCTTGCTG
GCCGCCTCTCGTGCTGCAATGCTATTTAATCCACACGACTCTCGGCAACGGATATCTCGG
CTCTCGCATCGATGAAGAACGTAGCGAAATGCGATACCTGGTGTGAATTGCAGAATCCCG
CGAACCATCGAGTCTTTGAACGCAAGTTGCGCCCGAGGCCATTCGGCTGAGGGCACGCCT
GCCTGGGCGTCACGTAAAACACGCTCCCACACCCTCATCGGGTAGCGGGACGCGGCATGT
GGTCCCCCGTAGCGCAAGCTGCGGTGGGCCGAAGATATGGCTGCCGGCGTATCGTGCCGG
ACACAGCGCGTGGTGGGCGACCTCGCTTTACTTACCGCAGTGCATCCGGGGCGTAGCCGA
CGTGATGGCCTCTAAGGACCCTATGAACGGTGCGCACGACG
>ITS.13_4_3_C6Frc
GGAAGTAAAAGTCGTAACAAGGTTTCCGTAGGTGAACCTGCGGAAGGATCATTGTCGTGA
CCCTGACCAAAATAGACCGTGCACGCGTCATCCAGCCTGCCGGCGGCGGCACCGTTCGTC
GCTCGGCCAAGTCCTCGACAACCTCCTCTCCTCGGAGTGGGGGCTCGGGGTAAAAGAACC
CACGACGCCTAAGGCGTCAAGGAACACTGTGCCTAGCCCGGGGACGCGGACGGCTTGCTG
GCCGCCCCTCGTGCTGCAATGCTATTTAATCCACACGACTCTCGGCAACGGATATCTCGG
CTCTCGCATCGATGAAGAACGTAGCGAAATGCGATACCTGGTGTGAATTGCAGAATCCCG
CGAACCATCGAGTCTTTGAACGCAAGTTGCGCCCGAGGCCATTCGGCTGAGGGCACGCCT
GCCTGGGCGTCACGTAAAACACGCTCCCACACCCTCATCGGGTAGCGGGACGCGGCATGT
GGTCCCCCGTAGCGCAAGCTGCGGTGGGCCGAAGATATGGCTGCCGGCGTATCGTGCCGG
ACACAGCGCGTGGTGGGCGACCTCGCTTTACTTACCGCAGTGCATCCGGGGCGTAGCCGA
CGTGATGGCCTCTAAGGACCCTATGAACGGTGCGCACGACG
>ITS.13_4_3_C7F
GGAAGTAAAAGTCGTAACAAGGTTTCCGTAGGTGAACCTGCGGAAGGATCATTGTCGTGA
CCCTGACCAAAATAGACCGTGCACGCGTCATCCAGCCTGCCGGCGGCGGCACCGTTCGTC
GCTCGGCCAAGTCCTCGACAACCTCCTCTCCTCGGAGTGGGGGCTCGGGGTAAAAGAACC
CACGACGCCTAAGGCGTCAAGGAACACTGTGCCTAGCCCGGGGACGCGGACGGCTTGCTG
GCCGCCCCCCGTGCTGCAATGCTATTTAATCCACACGACTCTCGGCAACGGATATCTCGG
CTCTCGCATCGATGAAGAACGTAGCGAAATGCGATACCTGGTGTGAATTGCAGAATCCCG
CGAACCATCGAGTCTTTGAACGCAAGTTGCGCCCGAGGCCATTCGGCTGAGGGCACGCCT
GCCTGGGCGTCACGCAAAACACGCTCCCACACCCTCATCGGGTAGCGGGACGCGGCTTGT
GGTTCCCCGTAGCGCAAGCTGCGGTGGGCCGAAGATATGGCTGCCGGCGTATCGTGCCGG
ACACAGCGCGTGGTGGGCGACCTCGCTTTACTTACCGCAGTGCATCCGGGGCGTAGCCGA
CGTGATGGCCTCTAAGGACCCTATGAACGGTGCGCACGACG

>ITS.13_4_3_C8Frc
GGAAGTAAAAGTCGTAACAAGGTTTCCGTAGGTGAACCTGCGGAAGGATCATTGTCGTGA
CCCTGACCAAAATAGACCGTGCACGCGTCATCCAGCCTGCCGGCGGCGGCACCGTTCGTC
GCTCGGCCAAGTCCTCGACAACCTCCTCTCCTCGGAGTGGGGGCTCGGGGTAAAAGAACC
CACGACGCCTAAGGCGTCAAGGAACACTGTGCCTAGCCCGGGGACGCGGACGGCTTGCTG
GCCGCCCCTCGTGCTGCAATGCTATTTAATCCACACGACTCTCGGCAACGGATATCTCGG
CTCTCGCATCGATGAAGAACGTAGCGAAATGCGATACCTGGTGTGAATTGCAGAATCCCG
CGAACCATCGAGTCTTTGAACGCAAGTTGCGCCCGAGGCCATTCGGCTGAGGGCACGCCT
GCCTGGGCGTCACGTAAAACACGCTCCCACACCCTCATCGGGTAGCGGGACGCGGCATGT
GGTCCCCCGTAGCGCAAGCTGCGGTGGGCCGAAGATATGGCTGCCGGCGTATCGTGCCGG
ACACAGCGCGTGGTGGGCGACCTCGCTTTACTTACCGCAGTGCATCCGGGGCGTAGCCGA
CGTGATGGCCTCTAAGGACCCTTTGAACGGTGCGCACGACG
>ITS.13_4_3_C9Frc
GGAAGTAAAAGTCGTAACAAGGTTTCCGTAGGTGAACCTGCGGAAGGATCATTGTCGTGA
CCCTGACCAAAATAGACCGTGGACGCGTCATCCAGCCTGCTGGCGGCGGCACCGTTCATC
GCTTAGCCAAGTCCTCGACAACCTCCTCTCCTCGGAGTGGGGGCTCGGGGTAAAAGAACC
CACGACGCCTAAGGCGTCAAGGAACACTGTGCCTAGCCCGGGGACGCGGACGGCTTGCTG
GCCGCCCCCCGTGCTGCAATGCTATTTAATCCACACGACTCTCGGCAACGGATATCTCGG
CTCTCGCATCGATGAAGAACGTAGCGAAATGCGATACCTGGTGTGAATTGCAGAATCCCG
CGAACCATCGAGTCTTTGAACGCAAGTTGCGCCCGAGGCCATTCGGCTGAGGGCACGCCT
GCCTGGGCGTCACGTAAAACACGCTCCCACACCCTCATCGGGTAGCGGGACGCGGCATGT
GGTCCCCCGTAGCGCAAGCTGCGGTGGGCCGAAGATATGGCTGCCGGCGTATCGTGCCGG
ACACAGCGCGTGGTGGGCGACCTCGCTTTACTTACCGCAGTGCATCCGGGGCGTAGCCGA
CGTGATGGCCTCTAAGGACCCTTTGAACGGTGCGCACGACG
>ITS.13_4_4_C1R
GGAAGTAAAAGTCGTAACAAGGTTTCCGTAGGTGAACCTGCGGAAGGATCATTGTCGTGA
CCCTGACCAAAATAGACCGTGCACGCGTCATCCAGCCTGCCGGCGGCGGCACCGTTCGTC
GCTCGGCCAAGTCCTCGACAACCTCCTCTCCTCGGAGTGGGGGCTCGGGGTAAAAGAACC
CACGACGCCTAAGGCGTCAAGGAACACTGTGCCTAGCCCGGGGACGCGGACGGCTTGCTG
GCCGCCCCCCGTGCTGCAATGCTATTTAATCCACACGACTCTCGGCAACGGATATCTCGG
CTCTCGCATCGATGAAGAACGTAGCGAAATGCGATACCTGGTGTGAATTGCAGAATCCCG
CGAACCATCGAGTCTTTGAACGCAAGTTGCGCCCGAGGCCATTCGGTTGAGGGCACGCCT
GCCTAGGCGTCACGCAAAACACGCTCCCACACCCTCATCGGGTAGCGGGACGCGGCATGT
GGTCCCCCGTAGCGCAAGCTGCGGTGGGCCGAAGATATGGCTGCCGGCGTATCGTGCCGG
ACACAGCGCGTGGTGGGCGACCTCGCTTTACTTACCGCAGTGCATCCGGGGCGTAGCCGA
CGTGATGGCCTCTAAGGACCCTTTGAACGGTGCGCACGACG
>ITS.13_4_4_C2R
GGAAGTAAAAGTCGTAACAAGGTTTCCGTAGGTGAACCTGCGGAAGGATCATTGTCGTGA
CCCTGACCAAAATAGACCGTGCACGCGTCATCCAGCCTGCCGGCGGCGGCACCGTTCGTC
GCTCGGCCAAGTCCTCGACAACCTCCTCTCCTCGGAGTGGGGGCTCGGGGTAAAAGAACC
CACGACGCCTAAGGCGTCAAGGAACACTGTGCCTAGCCCGGGGACGCGGACGGCTTGCTG
GCCGCCCCCCGTGCTGCAATGCTATTTAATCCACACGACTCTCGGCAGCGGATATCTCGG
CTCTCGCATCGATGAAGAACGTAGCGAAATGCGATACCTGGTGTGAATTGCAGAATCCCG
CGAACCATCGAGTCTTTGAACGCAAGTTGCGCCCGAGGCCATTCGGCTGAGGGCACGCCT
GCCTGGGCGTCACGCAAAACACGCTCCCACACCCTCATCGGGTAGCGGGACGCGGCATGT
GGTCCCCCGTAGCGCAAGCTGCGGTGGGCCGAAGATATGGCTGCCGGCGTATCGTGCCGG
ACACAGCGCGTGGTGGGCGACCTCGCTTTACTTACCGCAGTGCATCCGGGGCGTAGCCGA
CGTGATGGCCTCTAAGGACCCTTTGAACGGTGCGCACGACG
>ITS.13_4_4_C3F
GGAAGTAAAAGTCGTAACAAGGTTTCCGTAGGTGAACCTGCGGAAGGATCATTGTCGTGA
CCCTGACCAAAATAGACCGTGCACGCGTCATCCAGCCTGCCGGCGGCGGCACCGTTCGTC
GCTCGGCCAAGTCCTCGACAACCTCCTCTCCTCGGAGTGGGGGCTCGGGGTAAAAGAACC
CACGACGCCTAAGGCGTCAAGGAACACTGTGCCTAGCCCGGGGACGCGGACGGCTTGCTG
GCCGCCCCCCGTGCTGCAATGCTATTTAATCCACACGACTCTCGGCAACGGATATCTCGG

CTCTCGCATCGATGAAGAACGTAGCGAAATGCGATACCTGGTGTGAATTGCAGAATCCCG
CGAACCATCGAGTCTTTGAACGCAAGTTGCGCCCGAGGCCATTCGGCTGAGGGCACGCCT
GCCTGGGCGTCACGCAAAACACGCTCCCACACCCTCATCGGGTAGCGGGACGCGGCATGT
GGTCCCCCGTAGCGCAAGCTGCGGTGGGCCGAAGATATGGCTGCCGGCGTATCGTGCCGG
ACACAGCGCGTGGTGGGCGACCTCGCTTTACTTACCGCAGTGCATCCGGGGCGTAGCCGA
CGTGATGGCCTCTAAGGACCCTATGAACGGTGCGCACGACG
>ITS.13_4_4_C4Frc
GGAAGTAAAAGTCGTAACAAGGTTTCCGTAGGTGAACCTGCGGAAGGATCATTGTCGTGA
CCCTGACCAAAATAGACCGTGCACGCGTCATCCAGCCTGCCGGCGGCGGCACCGTTCGTC
GCTCGGCCAAGTCCTCGACAACCTCCTCTCCTCGGAGTGGGGGCTCGGGGTAAAAGAACC
CACGACGCCTAAGGCGTCAAGGAACACTGTGCCTAGCCCGGGGACGCGGACGGCTTGCTG
GCCGCCCCCCGTGCTGCAATGCTATTTAATCCACACGACTCTCGGCAACGGATATCTCGG
CTCTCGCATCGATGAAGAACGTAGCGAAATGCGATACCTGGTGTGAATTGCAGAATCCCG
CGAACCATCGAGTCTTTGAACGCAAGTTGCGCCCGAGGCCATTCGGCTGAGGGCACGCCT
GCCTGGGCGTCACGCAAAACACGCTCCCACACCCTCATCGGGTAGCGGGACGCGGCATGT
GGTCCCCCGTAGCGCAAGCTGCGGTGGGCCGAAGATATGGCTGCCGGCGTATCGTGCCGG
ACACAGCGCGTGGTGGGCGACCTCGCTTTACTTACCGCAGTGCATCCGGGGCGTAGCCGA
CGTGATGGCCTCTAAGGACCCTATGAACGGTGCGCACGACG
>ITS.13_4_4_C5F
GGAAGTAAAAGTCGTAACAAGGTTTCCGTAGGTGAACCTGCGGAAGGATCATTGTCGTGA
CCCTGACCAAAATAGACCGTGCACGCGTCATCCAGCCTGCCGGCGGCGGCACCGTTCGTC
GCTCGGCCAAGTCCTCGACAACCTCCTCTCCTCGGAGTGGGGGCTCGGGGTAAAAGAACC
CACGACGCCTAAGGCGTCAAGGAACACTGTGCCTAGCCCGGGGACGCGGACGGCTTGCTG
GCCGCCCCCCGTGCTGCAATGCTATTTAATCCACACGACTCTCGGCAACGGATATCTCGG
CTCTCGCATCGATGAAGAACGTAGCGAAATGCGATACCTGGTGTGAATTGCAGAATCCCG
CGAACCATCGAGTCTTTGAACGCAAGTTGCGCCCGAGGCCATTCGGCTGAGGGCACGCCT
GCCTGGGCGTCACGCAAAACACGCTCCCACACCCTCATCGGGTAGCGGGACGCGGCATGT
GGTCCCCCGTAGCGCAAGCTGCGGTGGGCCGAAGATATGGCTGCCGGCGTATCGTGCCGG
ACACAGCGCGTGGTGGGCGACCTCGCTTTACTTACCGCAGTGCATCCGGGGCGTAGCCGA
CGTGATGGCCTCTAAGGACCCTATGAACGGTGCGCACGACG
>ITS.13_4_4_C6Frc
GGAAGTAAAAGTCGTAACAAGGTTTCCGTAGGTGAACCTGCGGAAGGATCATTGTCGTGA
CCCTGACCAAAATAGACCGTGCACGCGTCATCCAGCCTGCCGGCGGCGGCACCGTTCGTC
GCTCGGCCAAGTCCTCGACAACCTCCTCTCCTCGGAGTGGGGGCTCGGGGTAAAAGAACC
CACGACGCCTAAGGCGTCAAGGAACACTGTGCCTAGCCCGGGGACGCGGACGGCTTGTTG
GCCGCCCCCCGTGCTGCAATGCTATTTAATCCACACGACTCTCGGCAACGGATATCTCGG
CTCTCGCATCGATGAAGAACGTAGCGAAATGCGATACCTGGTGTGAATTGCAGAATCCCG
CGAACCATCGAGTCTTTGAACGCAAGTTGCGCCCGAGGCCATTCGGCTGAGGGCACGCCT
GCCTGGGCGTCACGCAAAACACGCTCCCACACCCTCATCGGGTAGCGGGACGCGGCATGT
GGTCCCCCGTAGCGCAATCTGCGGTGGGCCGAAGATATGGCTGCCGGCGTATCGTGCCGG
ACACAGCGCGTGGTGGGCGACCTCGCTTTACTTACCGCAGTGCATCCGGGGCGTAGCCGA
CGTGATGGCCTCTAAGGACCCTTTGAACGGTGCGCACGACG
>ITS.13_4_4_C7F
GGAAGTAAAAGTCGTAACAAGGTTTCCGTAGGTGAACCTGCGGAAGGATCATTGTCGTGA
CCCTGACCAAAATAGACCGTGCACGCGTCATCCAGCCTGCCGGCGGCGGCACCGTTCGTC
GCTCGGCCAAGTCCTCGACAACCTCCTCTCCTCGGAGTGGGGGCTCGGGGTAAAAGAACC
CACGACGCCTAAGGCGTCAAGGAACACTGTGCCTAGCCCGGGGACGCGGACGGCTTGCTG
GCCGCCCCCCGTGCTGCAATGCTATTTAATCCACACGACTCTCGGCAACGGATATCTCGG
CTCTCGCATCGATGAAGAACGTAGCGAAATGCGATACCTGGTGTGAATTGCAGAATCCCG
CGAACCATCGAGTCTTTGAACGCAAGTTGCGCCCGAGGCCATTCGGCTGAGGGCACGCCT
GCCTGGGCGTCACGCAAAACACGCTCCCACACCCTCATCGGGTAGCGGGACGCGGCATGT
GGTCCCCCGTAGCGCAAGCTGCGGTGGGCCGAAGATATGGCTGCCGGCGTATCGTGCCTG
ACACAGCGCGTGGTGGGCGACCTCGCTTTACTTACCGCAGTGCATCCGGGGCGTAGCCGA
CGTGATGGCCTCTAAGGACCCTATGAACGGTGCGCACGACG

>ITS.13_4_4_C10Frc
GGAAGTAAAAGTCGTAACAAGGTTTCCGTAGGTGAACCTGCGGAAGGATCATTGTCGTGA
CCCTGACCAAAATAGACCGTGCACGCGTCATCCAGCCTGCCGGCGGCGGCACCGTTCGTC
GCTCGGCCAAGTCCTCGACAACCTCCTCTCCTCGGAGTGGGGGCTCGGGGTAAAAGAACC
CACGACGCCTAAGGCGTCAAGGAACACTGTGCCTAGCCCGGGGACGCGGACGGCTTGCTG
GCCGCCCCCCGTGTTGCAATGCTATTTAATCCACACGACTCTCGGCAACGGATATCTCGG
CTCTCGCATCGATGAAGAACGTAGCGAAATGCGATACCTGGCGTGAATTGCAGAATCCCG
CGAACCATCGAGTCTTTGAACGCAAGTTGCGCCCGAGGCCATTCGGCTGAGGGCACGCCT
GCCTGGGCGTCACGCAAAACACGCTCCCACACCCTCATCGGGTAGCGGGACGCGGCATGT
GGTCCCCCGTAGCGCAAGCTGCGGTGGGCCGAAGATATGGCTGCCGGCGTATCGTGCCGG
ACACAGCGCGTGGTGGGCGACCTCGCTTTACTTACCGCAGTGCATCCGGGGCGTAGCCGA
CGTGATGGCCTCTAAGGACCCTATGAACGGTGCGCACGACG
>ITS.13_6_C1R
GGAAGTAAAAGTCGTAACAAGGTTTCCGTAGGTGAACCTGCGGAAGGATCATTGTCGTGA
CCCTGACCAAAATAGACCGTGCACGCGTCATCCAGCCTGCCGGCGGCGGCACCGTTCGTC
GCTCGGCCAAGTCCTCGACAACCTCCTCTCCTCGGAGTGGGGGCTCGGGGTAAAAGAACC
CACGACGCCTAAGGCGTCAAGGAACACTGTGCCTAGCCCGGGGACGCGGACGGCTTGCTG
GCCGCCCCCCGTGCTGCAATGCTATTTAATCCACACGACTCTCGGCAACGGATATCTCGG
CTCTCGCATCGATGAAGAACGTAGCGAAATGCGATACCTGGTGTGAATTGCAGAATCCCG
CGAACCATCGAGTCTTTGAACGCAAGTTGCGCCCGAGGCCATTCGGCTGAGGGCACGCCT
GCCTGGGCGTCACGCAAAACACGCTCCCACACCCTCATCGGGTAGCGGGACGCGGCATGT
GGTCCCCCGTAGCGCAAGCTGCGGTGGGCCGAAGATATGGCTGCCGGCGTATCGTGCCGG
ACACAGCGCGTGGTGGGCGACCTCGCTTTACTTACCGCAGTGCATCCGGGGCGTAGCCGA
CGTGATGGCCTCTAAGGACCCTATGAACGGTGCGCACGACG
>ITS.13_6_C2R
GGAAGTAAAAGTCGTAACAAGGTTTCCGTAGGTGAACCTGCGGAAGGATCATTGTCGTGA
CCCTGACCAAAATTGACCGTGCACGCGTCACCCAGCCTGCCGGCGGCGGCACCGTTCGTC
GCTCGGCCAAGTCCTCGACAACCTCCTCTCCTCGGAGTGGGGGCTCGGGGTAAAAGAACC
CACGACGCCTAAGGCGTCAAGGAACACTGTGCCTAGCCCGGGGACGCGGACGGCTTGCTG
GCCGCCCCCCGTGCTGCAATGCTATTTAATCCACACGACTCTCGGCAACGGATATCTCGG
CTCTCGCATCGATGAAGAACGTAGCGAAATGCGATACCTGGTGTGAATTGCAGAATCCCG
CGAACCATCGAGTCTTTGAACGCAAGTTGCGCCCGAGGCCATTCGGCTGAGGGCACGCCT
GCCTGGGCGTCACGCAAAACACGCTCCCACACCCTCATCGGGTAGCGGGACGCGGCATGT
GGTCCCCCGTAGCGCAAGCTGCGGTGGGCCGAAGATATGGCTGCCGGCGTATCGTGCCGG
ACACAGCGCGTGGTGGGCGACCTCGCTTTACTTACCGCAGCGCATCCGGGGCGTAGCCGA
CGTGATGGCCTCTAAGGACCCTTTGAACGGTGCGCACGACG
>ITS.13_6_C3F
GGAAGTAAAAGTCGTAACAAGGTTTCCGTAGGTGAACCTGCGGAAGGATCATTGTCGTGA
CCCTGACCAAAATAGACCGTGCACGCGTCATCCAGCCTGCCGGCGGCGGCACCGTTCGTC
GCTCGGCCAAGTCCTCGACAGCCTCCTCTCCTCGGAGTGGGGGCTCGGGGTAAAAGAACC
CACGACGCCTAAGGCGTCAAGGAACACTGTGCCTAGCCCGGGGACGCGGACGGCTTGCTG
GCCGCCCCCCGTGCTGCAATGCTATTTAATCCACACGACTCTCGGCAACGGATATCTCGG
CTCTCGCATCGATGAAGAACGTAGCGAAATGCGATACCTGGTGTGAATTGCAGAATCCCG
CGAACCATCGAGTCTTTGAACGCAAGTTGCGCCCGAGGCCATTCGGTTGAGGGCACGCCT
GCCTGGGCGTCACGCAAAACACGCTCCCACACCCTCATCGGGTAGCGGGACGCGGCATGT
GGTCCCCCGTAGCGCAAGCTGCGGTGGGCCGAAGATATGGCTGCCGGCGTATCGTGCCGG
ACACAGCGCGTGGTGGGCGACCTCGCTTTACTTACCGCAGTGCATCCGGGGCGTAGCCGA
CGTGATGGCCTCTAAGGACCCTATGAACGGTGCGCACGACG
>ITS.13_6_C4Frc
GGAAGTAAAAGTCGTAACAAGGTTTCCGTAGGTGAACCTGCGGAAGGATCATTGTCGTGA
CCCTGACCAAAATAGACCGTGCACGCGTCATCCAGCCTGCCGGCGGCGGCACCGTTCGTC
GCTCGGCCAAGTCCTCGACAACCTCCTCTCCTCGGAGTGGGGGCTCGGGGTAAAAGAACC
CACGACGCCTAAGGCGTCAAGGAACACTGTGCCTAGCCCGGGGACGCGGACGGCTTGCTG
GCCGCCCCCCGTGCTGCAATGCTATTTAATCCACACGACTCTCGGCAACGGATATCTCGG

CTCTCGCATCGATGAAGAACGTAGCGAAATGCGATACCTGGTGTGAATTGCAGAATCCCG
CGAACCATCAAGTCTTTGAACGCAAGTTGCGCCCGAGGCCATTCGGCTGAGGGCACGCCT
GCCTGGGCGTCACGTAAAACACGCTCCCACACCCTCATCGGGTAGCGGGACGCGGCATGT
GGTCCCCCGTAGCGCAAGCTGCGGTGGGCCGAAGATATGGCTGCCGGCGTATCGTGCCGG
ACACAGCGCGTGGTGGGCGACCTCGCTTTACTTACCGCAGTGCATCCGGGGCGTAGCCGA
CGTGATGGCCTCTAAGGACCCTATGAACGGTGCGCACGACG
>ITS.13_6_C5Frc
GGAAGTAAAAGTCGTAACAAGGTTTCCGTAGGTGAACCTGCGGAAGGATCATTGTCGTGA
CCCTGACCAAAATAGACCGTGCACGCGTCATCCAGCCTGCCGGCGGCGGCACCGTTCGTC
GCTCGGCCAAGTCCTCGACAGCCTCCTCTCCTCGGAGTGGGGGCTCGGGGTAAAAGAACC
CACGACGCCTAAGGCGTCAAGGAACACTGTGCCTAGCCCGGGGACGCGGACGGCTTGCTG
GCCGCCCCCCGTGCTGCAATGCTATTTAATCCACACGACTCTCGGCAACGGATATCTCGG
CTCTCGCATCGATGAAGAACGTAGCGAAATGCGATACCTGGTGTGAATTGCAGAATCCCG
CGAACCATCGAGTCTTTGAACGCAAGTTGCGCCCGAGGCCATTCGGCTGAGGGCACGCCT
GCCTGGGCGTCACGCAAAACACGCTCCCACACCCTCATCGGGTAGCGGGACGCGGCATGT
GGTCCCCCGTAGCGCAAGCTGCGGTGGGCCGAGGATATGGCTGCCGGCGTATCGTGCCGG
ACACAGCGCGTGGTGGGCGACCTCGCTTTACTTACCGCAGTGCATCCGGGGCGTAGCCGA
CGTGATGGCCTCTAAGGACCCTTTGAACGGTGCGCACGACG
>ITS.13_6_C7Frc
GGAAGTAAAAGTCGTAACAAGGTTTCCGTAGGTGAACCTGCGGAAGGATCATTGTCGTGA
CCCTGACCAAAATAGACCGTGCACGCGTCATCCAGCCTGCCGGCGGCGGCACCGTTCGTC
GCTCGGCCAAGTCCTCGACAACCTCCTCTCCTCGGAGTGGGGGCTCGGGGTAAAAGAACC
CACGACGCCTAAGGCGTCAAGGAACACTGTGCCTAGCCCGGGGACGCGGACGGCTTGCTG
GCCGCCCCCCGTGCTGCAATGCTATTTAATCCACACGACTCTCGGCAACGGATATCTCGG
CTCTCGCATCGATGAAGAACGTAGCGAAATGCGATACCTGGTGTGAATTGCAGAATCCCG
CGAACCATCGAGTCTTTGAACGCAAGTTGCGCCCGAGGCCATTCGGTTGAGGGCACGCCT
GCCTGGGCGTCACGCAAAACACGCTCCCACACCCTCATCGGGTAGCGGGACGCGGCATGT
GGTCCCCCGTAGCGCAAGCTGCGGTGGGCCGAAGATATGGCTGCCGGCGTATCGTGCCGG
ACACAGCGCGTGGTGGGCGACCTCGCTTTACTTACCGCAGTGCATCCGGGGCGTAGCCGA
CGTGATGGCCTCTAAGGACCCTTTGAACGGTGCGCACGACG
>ITS.13_6_C8F
GGAAGTAAAAGTCGTAACAAGGTTTCCGTAGGTGAACCTGCGGAAGGATCATTGTCGTGA
CCCTGACCAAGATAGACCGTGCACGCGTCATCCAGCCTGCCGGCGGCGGCACCGTTCGTC
GCTCGGCCAAGTCCTCGACAACCTCCTCTCCTCGGAGTGGGGGCTCGGGGTAAAAGAACC
CACGACGCCTAAGGCGTCAAGGAACACTGTGCCTAGCTCGGGGACGCGGACGGCTTGCTG
GCCGCCCCTCGTGCTGCAATGCTATTTAATCCACACGACTCTCGGCAACGGATATCTCGG
CTCTCGCATCGATGAAGAACGTAGCGAAATGCGATACCTGGTGTGAATTGCAGAATCCCG
CGAACCATCGAGTCTTTGAACGCAAGTTGCGCCCGAGGCCATTCGGCTGAGGGCACGCCT
GCCTGGGCGTCACGCAAAACACGCTCCCACACCCTCATCGGGTAGCGGGACGCGGCATGT
GGTCCCCCGTAGCGCAAGCTGCGGTGGGCCGAAGATATGGCTGCCGGCTTATCGTGCCGG
ACACAGCGCGTGGTGGGCGACCTCGCTTTACTTACCGCAGTGCATCCGGGGCGTAGCCGA
CGTGATGGCCTCTAAGGACCCTATGAACGGTGCGCACGACG
>ITS.13_6_C9Frc
GGAAGTAAAAGTNGTAACAAGGTTTCCGTAGGTGAACCTGCGGAAGGATCATTGTCGTGA
CCCTGACCAAAATAGACCGTGCACGCGTCATCCAGCCTGCCGGCGGCGGCACCGTTCGTC
GCTCGGCCAAGTCCTCGACAACCTCCTCTCCTCGGAGTGGGGGCTCGGGGTAAAAGAACC
CACGACGCCTAAGGCGTCAAGGAACACTGTGCCTAGCCCGGGGACGCGGACGGCTTGCTG
GCCGCCCCCCGTGCTGCAATGCTATTTAATCCACACGACTCTCGGCAATGGATATTTCGG
CTCTCGCATCGATGAAGAACGTAGTGAAATGCGATACCTGGTGTGAATTGCAGAATCCCG
CGAACCATCGAGTCTTTGAACGCAAGTTGCGCCCGAGGCCATTCGGCTGAGGGCACGCCT
GCCTGGGCGTCACGCAAAACACGCTCCCACACCCTCATCGGGTAGCGGGACGCGGCATGT
GGTCCCCCGTAGCGCAAGCTGCGGTGGGCCGAAGATATGGCTGCCGGCGTATCGTGCCGG
ACACAGCGCGTGGTGGGCGACCTCGCTTTACTTACCGCAGTGCATCCGGGGCGTAGCCGA
CGTGATGGCCTCTAAGGACCCTATGAACGGTGCGCACGACG

>ITS.090_C1R
GGAAGTAAAAGTCGTAACAAGGTTTCCGTAGGTGAACCTGCGGAAGGATCATTGTCGTGA
CCCTGACCAAAATAGACCGTGCACGCGTCATCCAGCCTGCCGGCGGCGGCACCGTTCGTC
GCTCGGCCAAGTCCTCGACAACCTCCTCTCCTCGGAGTGGGGGCTCGGGGTAAAAGAACC
CACGACGCCTAAGGCGTCAAGGAACACTGTGCCTAGCCCGGGGACGCGGACGGCTTGCTG
GCCGCCCCCCGTGCTGCAATGCTATTTAATCCACACGACTCTCGGCAACGGATATCTCGG
CTCTCGCATCGATGAAGAACGTAGCGAAATGCGATACCTGGTGTGAATTGCAGAATCCCG
CGAACCATCGAGTCTTTGAACGCAAGTTGCGCCCGAGGCCATTCGGTTGAGGGCACGCCT
GCCTGGGCGTCACGCAAAACACGCTCCCACACCCTCATCGGGTAGCGGGACGCGGCATGT
GGTCCCCCGTAGCGCAAGCTGCGGTGGGCCGAAGATATGGCTGCCGGCGTATCGTGCCGG
ACACAGCGCGTGGTGGGCGACCTCGCTTTACTTACCGCAGTGCATCCGGGGCGTAGCCGA
CGTGATGGCCTCTAAGGACCCTTTGAACGGTGCGCACGACG
>ITS.090_C2R
GGAAGTAAAAGTCGTAACAAGGTTTCCGTAGGTGAACCTGCGGAAGGATCATTGTCGTGA
CCCTGACCAAAATAGACCGTGCACGCGTCATCCAGCCTGCCGGCGGCGGCACCGTTCGTC
GCTCGGCCAAGTCCTCGACAACCTCCTCTCCTCGGAGTGGGGGCTCGGGGTAAAAGAACC
CACGACGCCTAAGGCGTCAAGGAACACTGTGCCTAGCCCGGGGACGCGGACGGCTTGCTG
GCCGCCCCCCGTGCTGCAATGCTATTTAATCCACACGACTCTCGGCAACGGATATCTCGG
CTCTCGCATCGATGAAGAACGTAGCGAAATGCGATACCTGGTGTGAATTGCAGAATCCCG
CGAACCATCGAGTCTTTGAACGCAAGTTGCGCCCGAGGCCATTCGGTTGAGGGCACGCCT
GCCTGGGCGTCACGCAAAACACGCTCCCACACCCTCATCGGGTAGCGGGACGCGGCATGT
GGTCCCCCGTAGCGCAAGCTGCGGTGGGCCGAAGATATGGCTGCCGGCGTATCGTGCCGG
ACACAGCGCGTGGTGGGCGACCTCGCTTTACTTACCGCAGTGCATCCGGGGCGTAGCCGA
CGTGATGGCCTCTAAGGACCCTTTGAACGGTGCGCACGACG
>ITS.090_C3R
GGAAGTAAAAGTCGTAACAAGGTTTCCGTAGGTGAACCTGCGGAAGGATCATTGTCGTGA
CCCTGACCAAAATAGACCGTGCACGCGTCATCCAGCCTGCCGGCGGCGGCACCGTTCGTC
GCTCGGCCAAGTCCTCGACAACCTCCTCTCCTCGGAGTGGGGGCTCGGGGTAAAAGAACC
CACGACGCCTAAGGCGTCAAGGAACACTGTGCCTAGCTCGGGGACGCGGACGGCTTGCTG
GCCGCCCCTCGTGCTGCAATGCTATTTAATCCACACGACTCTCGGCAACGGATATCTCGG
CTCTCGCATCGATGAAGAACGTAGCGAAATGCGATACCTGGTGTGAATTGCAGAATCCCG
CGAACCATCGAGTCTTTGAACGCAAGTTGCGCCCGAGGCCATTCGGCTGAGGGCACGCCT
GCCTGGGCGTCACGCAAAACACGCTCCCACACCCTCATCGGGTAGCGGGACGCGGCATGT
GGTCCCCCGTAGCGCAAGCTGCGGTGGGCCGAAGATATGGCTGCCGGCGTATCGTGCCGG
ACACAGCGCGTGGTGGGCGACCTCGCTTTACTTACCGCAGTGCATCCGGGGCGTAGCCGA
CGCGATGGCCTCTAAGGACCCTTTGAACGGTGCGCACGACG
>ITS.090_C4Frc_rev
GGAAGTAAAAGTCGTAACAAGGTTTCCGTAGGTGAACCTGCGGAAGGATCATTGTCGTGA
CCCTGACCAAAATAGACCGTGCACGCGTCATCCAGCCTGCCGGCGGCGGCACCGTTCGTC
GCTCGGCCAAGTCCTCGACAACCTCCTCTCCTCGGAGTGGGGGCTCGGGGTAAAAGAACC
CACGACGCCTAAGGCGTCAAGGAACACTGTGCCTAGCCCGGGGACGCGGACGGCTTGCTG
GCCGCCCCCCGTGCTGCAATGCTATTTAATCCACACGACTCTCGGCAACGGATATCTCGG
CTCTCGCATCGATGAAGAACGTAGCGAAATGCGATACCTGGTGTGAATTGCAGAATCCCG
CGAACCATCGAGTCTTTGAACGCAAGTTGCGCCCGAGGCCATTCGGTTGAGGGCACGCCT
GCCTGGGCGTCACGCAAAACACGCTCCCACACCCTCATCGGGTAGCGGGACGCGGCCTGT
GGTCCCCCGTAGCGCAAGCTGCGGTGGGCCGAAGATATGGCTGCCGGCGTATCGTGCCGG
ACACAGCGCGTGGTGGGCGACCTCGCTTTACTTACCGCAGTGCATCCGGGGCGTAGCCGA
CGTGATGGCCTCTAAGGACCCTATGAACGGTGCGCACGACG
>ITS.090_C5Frc_rev
GGAAGTAAAAGTCGTAACAAGGTTTCCGTAGGTGAACATGCGGAAGGATCATTGTCGTGA
CCCTGACCAAAACAGACCGCGCACGCATCATCCAGCCTGCCGGCGGCGACACCGTTCGTC
GCTCTTTCAAGTCCTCGATTCCTCCTCTCCTCGGAGTGGGGGCTCGGGGTAAAAGAACCC
ACGACGCCTTAAGGCGTCAAGGAACACTGTGCCTAGCCCGGGGACGCGGACGGCTTGCTG
GCCGCCCCCCGTGCTGCAATGCTATTTAATCCACACGACTCTCGGCAACGGATATCTCGG

CTCTCGCATCGATGAAGAACGTAGCGAAATGCGATACCTGGTGTGAATTGCAGAATCCCG
CGAACCATCGAGTCTTTGAACGCAAGTTGCGCCCGAGGCCATTCGGCTGAGGGCACGCCT
GCCTGGGCGTCACGCAAAACACGCTCCCACACCCTCATCGGGTAGCGGGACGCGGCATGT
GGTCCCCCGTAGCGCAAGCTGCGGTGGGCCGAAGATATGGCTGCCGGCGTATCGTGCCGG
ACACAGCGCGTGGTGGGCGACCTCGCTTTACTTACCGCAGTGCATCCGGGGCGTAGCCGA
CGTGATGGCCTCTAAGGACCCTATGAACGGTGCGCACGACG
>ITS.090_C6F
GGAAGTAAAAGTCGTAACAAGGTTTCCGTAGGTGAACCTGCGGAAGGATCATTGTCGTGA
CCCTGACCAAAATAGACCGTGCACGCGTCATCCAGCCTGCCGGCGGCGGCACCGTTCGTC
GCTCGGCCAAGTCCTCGACAACCTCCTCTCCTCGGAGTGGGGGCTCGGGGTAAAAGAACC
CACGACGCCTAAGGCGTCAAGGAACACTGTGCCTAGCCCGGGGACGCGGACGGCTTGCTG
GCCGCCCCCCGTGCTGCAATGCTATTTAATCCACACGACTCTCGGCAACGGATATCTCGG
CTCTCGCATCGATGAAGAACGTAGCGAAATGCGATACCTGGTGTGAATTGCAGAATCCCG
CGAACCATCGAGTCTTTGAACGCAAGTTGCGCCCGAGGCCATTCGGTTGAGGGCACGCCT
GCCTGGGCGTCACGCAAAACACGCTCCCACACCCTCATCGGGTAGCGGGACGCGGCCTGT
GGTCCCCCGTAGCGCAAGCTGCGGTGGGCCGAAGATATGGCTGCCGGCGTATCGTGCCGG
ACACAGCGCGTGGTGGGCGACCTCGCTTTACTTACCGCAGTGCATCCGGGGCGTAGCCGA
CGTGATGGCCTCTAAGGACCCTATGAACGGTGCGCACGACG
>ITS.090_C7F
GGAAGTAAAAGTCGTAACAAGGTTTCCGTAGGTGAACCTGCGGAAGGATCATTGTCGTGA
CCCTGACCAAAATAGACCGTGCACGCGTCATCCAGCCTGCCGGCGGCGGCACCGTTCGTC
GCTCGGCCAAGTCCTCGACAACCTCCTCTCCTCGGAGTGGGGGCTCGGGGTAAAAGAACC
CACGACGCCTAAGGCGTCAAGGAACACTGTGCCTAGCCCGGGGACGCGGACGGCTTGCTG
GCCGCCCCCGTGCTGCAATGCTATTTAATCCACACGACTCTCGGCAATGGATATCTCGGC
TCTCGCATCGATGAAGGACGTAGCGAAATGCGATACCTGGTGTGAATTGCAGAATCCCGC
GAACCATCGAGTCTTTGAACGCAAGTTGCGCCCGAGGCCATTCGGCTGAGGGCACGCCTG
CCTGGGCGTCACGCAAAACACGCTCCCACACCCTCATCGGGTAGCGGGACGCGGCATGTG
GTCCCCCGTAGCGCAAGCTGCGGTGGGCCGAAGATATGGCTGCCGGCGTATCGTGCCGGA
CACAGCGCGTGGTGGGCGACCTCGCTTTACTTACCGCAGTGCATCCGGGGCGTAGCCGAC
GTGATGGCCTCTAAGGACCCTATGAACGGTGCGCACGACG
>ITS.090_C8F
GGAAGTAAAAGTCGTAACAAGGTTTCCGTAGGTGAACCTGCGGAAGGATCATTGTCGTGA
CCCTGACCAAAATAGACCGTGCACGCGTCATCCAGCCTGCCGGCGGCGGCACCGTTCGTC
GCTCGGCCAAGTCCTCGACAACCTCCTCTCCTCGGAGTGGGGGCTCGGGGTAAAAGAACC
CACGACGCCTAAGGCGTCAAGGAACACTGTGCCTAGCTCGGGGACGCGGACGGCTTGCTG
GCCGCCCCTCGTGCTGCAATGCTATTTAATCCACACGACTCTCGGCAACGGATATCTCGG
CTCTCGCATCGATGAAGAACGTAGCGAAATGCGATACCTGGTGTGAATTGCAGAATCCCG
CGAACCATCGAGTCTTTGAACGCAAGTTGCGCCCGAGGCCATTCGGTTGAGGGCACGCCT
GCCTGGGCGTCACGCAAAACACGCTCCCACACCCTCATCGGGTAGCGGGACGCGGCCTGT
GGTCCCCCGTAGCGCAAGCTGCGGTGGGCCGAAGATATGGCTGCCGGCGTATCGTGCCGG
ACACAGCGCGTGGTGGGCGACCTCGCTTTACTTACCGCAGTGCATCCGGGGCGTAGCCGA
CGTGATGGCCTCTAAGGACCCTATGAACGGTGCGCACGACG
>ITS.405_3_C1R
GGAAGTAAAAGTCGTAACAAGGTTTCCGTAGGTGAACCTGCGGAAGGATCATTGTCGTGA
CCCTGACCAAAATAGACCGTGCACGCGTCATCCAGCCCGCCGGCGGCGGCACCGTTCGTC
GCTCGGCCAAGTCCTCGACAACCTCCTCTCCTCGGAGTGGGGGCTCGGGGTAAAAGAACC
CACGACGCCTAAGGCGTCAAGGAACACTGTGCCTAGCCCGGGGACGCGGACGGCTTGCTG
GCCGCCCCCCGTGCTGCAATGCTATTTAATCCACACGACTCTCGGCAACGGATATCTCGG
CTCTCGCATCGATGAAGAACGTAGCGAAATGCGATACCTGGTGTGAATTGCAGAATCCCG
CGAACCATCGAGTCTTTGAACGCAAGTTGCGCCCGAGGCCATTCGGCTGAGGGCACGCCT
GCCTGGGCGTCACGCAAAACACGCTCCCACACCCTCATCGGGTAGCGGGACGCGGCATGT
GGTCCCCCGTAGCGCAAGCTGCGGTGGGCCGAAGATATGGCTGCCGGCGTATCGTGCCGG
ACACAGCGCGTGGTGGGCGACCTCGCTTTACTTACCGCAGTGCATCCGGGGCGTAGCCGA
CGTGATGGCCTCTAAGGACCCTTTGAACGGTGCGCACGACG

>ITS.405_3_C2F
GGAAGTAAAAGTCGTAACAAGGTTTCCGTAGGTGAACCTGCGGAAGGATCATTGTCGTGA
CCCTGACCAAAATAGACCGTGCACGCGTCATCCAGCCTGCCGGCGGCGGCACCGTTCGTC
GCTCGGCCAAGTCCTCGACAACCTCCTCTCCTCGGAGTGGGGGCTCGGGGTAAAAGAACC
CACGACGCCTAAGGCGTCAAGGAACACTGTGCCTAGCCCGGGGACGCGGACGGCTTGCTG
GCCGCCCCCCGTGCTGCAATGCTATTTAATCCACACGACTCTCGGCAACGGATATCTCGG
CTCTCGCATCGATGAAGAACGTAGCGAAATGCGATACCTGGTGTGAATTGCAGAATCCCG
CGAACCATCGAGTCTTTGAACGCAAGTTGCGCCCGAGGCCATTCGGCTGAGGGCACGCCT
GCCTGGGCGTCACGCAAAACACGCTCCCACACCCTCATCGGGTAGCGGGACGCGGCATGT
GGTCCCCCGTAGCGCAAGCTGCGGTGGGCCGAAGATATGGCTGCCGGCGTATCGTGCCGG
ACACAGCGCGTGGTGGGCGACCTCGCTTTACTTACCGCAGTGCATCCGGGGCATAGCCGA
CGTGATGGCCTCTAAGGACCCTATGAACGGTGCGCACGACG
>ITS.405_3_C3R
GGAAGTAAAAGTCGTAACAAGGTTTCCGTAGGTGAACCTGCGGAAGGATCATTGTCGTGA
CCCTGACCAAAATAGACCGTGCACGCGTCATCCACCCTGCCGGCGGCGGCACCGTTCGTC
GCTCGGCCAAGTCCTCGACAACCTCCTCTCCTCGGAGTGGGGGCTCGGGGTAAAAGAACC
CACGACGCCTAAGGCGTCAAGGAACACTGTGCCTAGCCCGGGGACGCGGACGGCTTGCTG
GCCGCCCCCCGTGCTGCAATGCTATTTAATCCACACGACTCTCGGCAACGGATATCTCGG
CTCTCGCATCGATGAAGAACGTAGCGAAATGCGATACCTGGTGTGAATTGCAGAATCCCG
CGAACCATCGAGTCTTTGAACGCAAGTTGCGCCCGAGGCCATTCGGCTGAGGGCACGCCT
GCCTGGGCGTCACGCAAAACACGCTCCCACACCCTCATCGGGTAGCGGGACGCGGCATGT
GGTCCCCCGTAGCGCAAGCTGCGGTGGGCCGAAGATATGGCTGCCGGCGTATCGTGCCGG
ACACAGCGCGTGGTGGGCGACCTCGCTTTACTTACCGCAGTGCATCCGGGGCGTAGCCGA
CGTGATGGCCTCTAAGGACCCTATGAACGGTGCGCACGACG
>ITS.405_3_C4Frc
GGAAGTAAAAGTCGTAACAAGGTTTCCGTAGGTGAACCTGCGGAAGGATCATTGTCGTGA
CCCTGACCAAAATAGACCGTGCACGCGTCATCCAGCCTGCCGGCGGCGGCACCGTTCGTC
GCTCGGCCAAGTCCTCGACAACCTCCTCTCCTCGGAGTGGGGGCTCGGGGTAAAAGAACC
CACGACGCCTAAGGCGTCAAGGAACACTGTGCCTAGCCCGGGGACGCGGACGGCTTGCTG
GCCGCCCCCCGTGCTGCAATGCTATTTAATCCACACGACTCTCGGCAACGGATATCTCGG
CTCTCGCATCGATGAAGAACGTAGCGAAATGCGATACCTGGTGTGAATTGCAGAATCCCG
CGAACCATCGAGTCTTTGAACGCAAGTTGCGCCCGAGGCCATTCGGTTGAGGGCACGCCT
GCCTGGGCGTCACGCAAAACACGCTCCCACACCCTCATCGGGTAGCGGGACGCGGCATGT
GGTCCCCCGTAGCGCAAGCTGCGGTGGGCCGAAGATATGGCTGCCGGCGTATCGTGCCGG
ACACAGCGCGTGGTGGGCGACCTCGCTTTATTTACCGCAGTGCATCCGGGGCGTAGCCGA
CGTGATGGCCTCTAAGGACCCTATGAACGGTGCGCACGACG
>ITS.405_3_C5Frc
GGAAGTAAAAGTCGTAACAAGGTTTCCGTAGGTGAACCTGCGGAAGGATCATTGTCGTGA
CCCTGACCAAAATAGACCGTGCACGCGTCATCCAGCCTGCCGGCGGCGGCACCGTTCGTC
GCTCGGCCAAGTCCTCGACAACCTCCTCTCCACGGAGTGGGGGCTCGGGGTAAAAGAACC
CACGACGCCTAAGGCGTCAAGGAACACTGTGCCTAGCCCGGGGACGCGGACGGCTTGCTG
GCCGCCCCCCGTGCTGCAATGCTATTTAATCCACACGACTCTCGGCAACGGATATCTCGG
CTCTCGCATCGATGAAGAACGTAGCGAAATGCGATACCTGGTGTGAATTGCAGAATCCCG
CGAACCATCGAGTCTTTGAACGCAAGTTGCGCCCGAGGCCATTCGGCTGAGGGCACGCCT
GCCTGGGCGTCACGCAAAACACGCTCCCACACCCTCATCGGGTAGCGGGACGCGGCATGT
GGTCCCCCGTAGCGCAAGCTGCGGTGGGCCGAAGATATGGCTGCCGGCGTATCGTGCCGG
ACACAGCGCGTGGTGGGCGACCTCGCTTTACTTACCGCAGTGCATCCGGGGCGTAGCCGA
CGTGATGGCCTCTAAGGACCCTATGAACGGTGCGCACGACG
>ITS.405_3_C6Frc
GGAAGTAAAAGTCGTAACAAGGTTTCCGTAGGTGAACCTGCGGAAGGATCATTGTCGTGA
CCCTGACCAAAATAGACCGTGCACGCGTCATCCAGCCTGCCGGCGGCGGCACCGTTCGTC
GCTCGGCCAAGTCCTCGACAACCTCCTCTCCACGGAGTGGGGGCTCGGGGTAAAAGAACC
CACGACGCCTAAGGCGTCAAGGAACACTGTGCCTAGCCCGGGGACGCGGACGGCTTGCTG
GCCGCCCCCCGTGCTGCAATGCTATTTAATCCACACGACTCTCGGCAACGGATATCTCGG

CTCTCGCATCGATGAAGAACGTAGCGAAATGCGATACCTGGTGTGAATTGCAGAATCCCG
CGAACCATCGAGTCTTTGAACGCAAGTTGCGCCCGAGGCCATTCGGCTGAGGGCACGCCT
GCCTGGGCGTCACGCAAAACACGCTCCCACACCCTCATCGGGTAGCGGGACGCGGCATGT
GGTCCCCCGTAGCGCAAGCTGCGGTGGGCCGAAGATATGGCTACCGGCGTATCGTGCCGG
ACACAGCGCGTGGTGGGCGACCTCGCTTTACTTACCGCAGTGCATCCGGGGCGTAGCCGA
CGTGATGGCCTCTAAGGACCCTATGAACGGTGCGCACGACG
>ITS.405_3_C7Frc
GGAAGTAAAAGTCGTAACAAGGTTTCCGTAGGTGAACCTGCGGAAGGATCATTGTCGTGA
CCCTGACCAAAATAGACCGTGCACGCGTCATCCAGCCTGCCGGCGGCGGCACCGTTCGTC
GCTCGGCCAAGTCCTCGACAACCTCCTCTCCTCGGAGTGGGGGCTCGGGGTAAAAGAACC
CACGACGCCTAAGGCGTCAAGGAACACTGTGCCTAGCCCGGGGACGCGGACGGCTTGCTG
GCCGCCCCCCGTGCTGCAATGCTATTTAATCCACACGACTCTCGGCAACGGATATCTCGG
CTCTCGCATCGATGAAGAACGTAGCGAAATGCGATACCTGGTGTGAATTGCAGAATCCCG
CGAACCATCGAGTCTTTGAACGCAAGTTGCGCCCGAGGCCATTCGGTTGAGGGCACGCCT
GCCTGGGCGTCACGCAAAACACGCTCCCGCACCCTCATCGGGTAGCGGGACGCGGCATGT
GGTCCCCCGTAGCGCAAGCTGCGGTGGGCCGAAGATATGGCTGCCGGCGTATCGTGCCGG
ACACAGCGCGTGGTGGGCGACCTCGCTTTACTTACCGCAGTGCATCCGGGGCGTAGCCGA
CGTGATGGCCTCTAAGGACCCTTTGAACGGTGCGCACGACG
>ITS.405_3_C8Frc
GGAAGTAAAAGTCGTAACAAGGTTTCCGTAGGTGAACCTGCGGAAGGATCATTGTCGTGA
CCCTGACCAAAATAGACCGTGCACGCGTCATCCAGCCTGCCGGCGGCGGCACCGTTCGTC
GCTCGGCCAAGTCCTCGACAACCTCCTCTCCTCGGAGTGGGGGCTCGGGGTAAAAGAACC
CACGACGCCTAAGGCGTCAAGGAACACTGTGCCTAGCCCGGGGACGCGGACGGCTTGCTG
GCCGCCCCCCGTGCTGCAATGCTATTTAATCCACACGACTCTCGGCAACGGATATCTCGG
CTCTCGCATCGATGAAGAACGTAGCGAAATGCGATACCTGGTGTGAATTGCAGAATCCCG
CGAACCATCGAGTCTTTGAACGCAAGTTGCGCCCGAGGCCATTCGGCTGAGGGCACGCCT
GCCTGGGCGTCACGCAAAACACGCTCCCACACCCTCATCGGGTAGCGGGACGCGGCATGT
GGTCCCCCGTAGCGCAATCTGCGGTGGGCCGAAGATATGGCTGCCGGCGTATCGTGCCGG
ACACAGCGCGTGGTGGGCGACCTCGCTTTACTTACCGCAGTGCATCCGGGGCGTAGCCGA
CGTGATGGCCTCTAAGGACCCTTTGAACGGTGCGCACGACG
>ITS.405_4_C1R
GGAAGTAAAAGTCGTAACAAGGTTTCCGTAGGTGAACCTGCGGAAGGATCATTGTCGTGA
CCCTGACCAAAATAGACCGTGCACGCGTCATCCAGCCTGCCGGCGGCGGCACCGTTCGTC
GCTCGGCCAAGTCCTCGACAACCTCCTCTCCTCGGAGTGGGGGCTCGGGGTAAAAGAACC
CACGACGCCTAAGGCGTCAAGGAACACTGTGCCTAGCCCGGGGACGCGGACGGCTTGCTG
GCCGCCCCCCGTGCTGCAATGCTATTTAATCCACACGACTCTCGGCAACGGATATCTCGG
CTCTCGCATCGATGAAGAACGTAGCGAAATGCGATACCTGGTGTGAATTGCAGAATCCCG
CGAACCATCGAGTCTTTGAACGCAAGTTGCGCCCGAGGCCATTCGGTTGAGGGCACGCCT
GCCTGGGCGTCACGCAAAACACGCTCCCACACCCTCATCGGGTAGCGGGACGCGGCCTGT
GGTCCCCCGTAGCGCAAGCTGCGGTGGGCCGAAGATATGGCTGCCGGCGTATCGTGCCGG
ACACAGCGCGTGGTGGGCGACCTCGCTTTACTTACCGCAGTGCATCCGGGGCGTAGGCGA
CGTGATGGCCTCTAAGGACCCTTTGAACGGTGCGCACGACG
>ITS.405_4_C3R
GGAAGTAAAAGTCGTAACAAGGTTTCCGTAGGTGAACCTGCGGAAGGATCATTGTCGTGA
CCCTGACCAAAATAGACCGTGCACGCGTCATCCAGCCTGCCGGCGGCGGCACCGTTCGTC
GCTCGGCCAAGTCCTCGACAACCTCCTCTCCTCGGAGTGGGGGCTCGGGGTAAAAGAACC
CACGACGCCTAAGGCGTCAAGGAACACTGTGCCTAGCCCGGGGACGCGGACGGCTTGCTG
GCCGCCCCCCGTGCTGCGATGCTATTTAATCCACACGACTCTCGGCAACGGATATCTCGG
CTCTCGCATCGATGAAGAACGTAGCGAAATGCGATACCTGGTGTGAATTGCAGAATCCCG
CGAACCATCGAGTCTTTGAACGCAAGTTGCGCCCGAGGCCATTCGGCTGAGGGCACGCCT
GCCTGGGCGTCACGCAAAACACGCTCCCACACCCTCATCGGGTAGCGGGACGCGGCATGT
GGTCCCCCGTAGCGCAAGCTGCGGTGGGCCGAAGATATGGCTGCCGGCGTATCGTGCCGG
ACACAGCGCGTGGTGGGCGACCTCGCTTTACTTACCGCAGTGCATCCGGGGCGTAGCCGA
CGTGATGGCCTCTAAGGACCCTATGAACGGTGCGCACGACG

>ITS.405_4_C4R
GGAAGTAAAAGTCGTAACAAGGTTTCCGTAGGTGAACCTGCGGAAGGATCATTGTCGTGA
CCCTGACCAAAATAGACCGTGCACGCGTCATCCAGCCTGCCGGCGGCGGCACCGTTCGTC
GCTCGGCCAAGTCCTCGACAACCTCCTCTCCTCGGAGTGGGGGCTCGGGGTAAAAGAACC
CACGACGCCTAAGGCGTCAAGGAACACTGTGCCTAGCCCGGGGACGCGGACGGCTTGCTG
GCCGCCCCCCGTGCTGCAATGCTATTTAATCCACACGACTCTCGGCAACGGATATCTCGG
CTCTCGCATCGATGAAGAACGTAGCGAAATGCGATACCTGGTGTGAATTGCAGAATCCCG
CGAACCATCGAGTCTTTGAACGCAAGTTGCGCCCGAGGCCATTCGGTTGAGGGCACGCCT
GCCTGGGCGTCACGCAAAACACGCTCCCACACCCTCATCGGGTAGCGGGACGCGGCCTGT
GGTCCCCCGTAGCGCAAGCTGCGGTGGGCCGAAGATATGGCTGCCGGCGTATCGTGCCGG
ACACAGCGCGTGGTGGGCGACCTCGCTTTACTTACCGCAGTGCATCCGGGGCGTAGCCGA
CGTGATGGCCTCTAAGGACCCTTTGAACGGTGCGCACGACG
>ITS.405_4_C5Frc
GGAAGTAAAAGTCGTAACAAGGTTTCCGTAGGTGAACCTGCGGAAGGATCATTGTCGTGA
CCCTGACCAAAATAGACCGTGCACGCGTCATCCAGCCTGCCGGCGGCGGCACCGTTCGTC
GCTCGGCCAAGTCCTCGACAACCTCCTCTCCTCGGAGTGGGGGCTCGGGGTAAAAGAACC
CACGACGCCTAAGGCGTCAAGGAACACTGTGCCTAGCTCGGGGACGCGGACGGCTTGCTG
GCCGCCCCTCGTGCTGCAATGCTATTTAATCCACACGACTCTCGGCAACGGATATCTCGG
CTCTCGCATCGATGAAGAACGTAGCGAAATGCGATACCTGGTGTGAATTGCAGAATCCCG
CGAACCATCGAGTCTTTGAACGCAAGTTGCGCCCGAGGCCATTCGGTTGAGGGCACGCCT
GCCTGGGCGTCACGCAAAACACGCTCCCACACCCTCATCGGGTAGCGGGACGCGGCATGT
GGTCCCCCGTAGCGCAAGCTGCGGTGGGCCGAAGATATGGCTGCCGGCGTATCGTGCCGG
ACACAGCGCGTGGTGGGCGACCTCGCTTTACTTACCGCAGTGCATCCGGGGCGTAGCCGA
CGTGATGGCCTCTAAGGACCCTTTGAACGGTGCGCACGACG
>ITS.405_4_C6F
GGAAGTAAAAGTCGTAACAAGGTTTCCGTAGGTGAACCTGCGGAAGGATCATTGTCGTGA
CCCTGACCAAAATAGACCGTGCACGCGTCATCCAGCCTGCCGGCGGCGGCACCGTTCGTC
GCTCGGCCAAGTCCTCGACAACCTCCTCTCCTCGGAGTGGGGGCTCGGGGTAAAAGAACC
CACGACGCCTAAGGCGTCAAGGAACACTGTGCCTAGCTCGGGGACGCGGACGGCTTGCTG
GCCGCCCCTCGTGCTGCAATGCTATTTAATCCACACGACTCTCGGCAACGGATATCTCGG
CTCTCGCATCGATGAAGAACGTAGCGAAATGCGATACCTGGTGTGAATTGCAGAATCCCG
CGAACCATCGAGTCTTTGAACGCAAGTTGCGCCCGAGGCCATTCGGCTGAGGGCACGCCT
GCCTGGGCGTCACGCAAGACACGCTCCCACACCCTCATCGGGTAGCGGGACGCGGCATGT
GGTCCCCCGTAGCGCAAGCTGCGGTGGGCCGAAGATATGGCTGCCGGCGTATCGTGCCGG
ACACAGCGCGTGGTGGGCGACCTCGCTTTACTTACCGCAGTGCATCCGGGGCGTAGCCGA
CGTGATGGCCTCTAAGGACCCTATGAACGGTGCGCACGACG
>ITS.405_4_C7Frc
GGAAGTAAAAGTCGTAACAAGGTTTCCGTAGGTGAACCTGCGGAAGGATCATTGTCGTGA
CCCTGACCAAAATAGACCGTGCACGCGTCATCCAGCCTGCCGGCGGCGGCACCGTTCGTC
GCTCGGCCAAGTCCTCGACAACCTCCTCTCCTCGGAGTGGGGGCTCGGGGTAAAAGAACC
CACGACGCCTAAGGCGTCAAGGAACACTGTGCCTAGCCCGGGGACGCGGACGGCTTGCTG
GCCGCCCCCCGTGCTGCAATGCTATTTAATCCACACGACTCTCGGCAACGGATATCTCGG
CTCTCGCATCGATGAAGAACGTAGCGAAATGCGATACCTGGTGTGAATTGCAGAATCCCG
CGAACCATCGAGTCTTTGAACGCAAGTTGCGCCCGAGGCCATTCGGCTGAGGGCACGCCT
GCCTGGGCGTCACGCAAAACACGCTCCCACACCCTCATCGGGTAGCGGGACGCGGCATGT
GGTCCCCCGTAGCGCAAGCTGCGGTGGGCCGAAGATATGGCTGCCGGCGTATCGTGCCGG
ACACAGCGCGTGGTGGGCGACCTCGCTTTACTTACCGCAGTGCATCCGGGGCGTAGCCGA
CGTGATGGCCTCTAAGGACCCTTTGAACGGTGCGCACGACG
>ITS.405_4_C8Frc
GGAAGTAAAAGTCGTAACAAGGTTTCCGTAGGTGAACCTGCGGAAGGATCATTGTCGTGA
CCCTGACCAAAATAGACCGTGCACGCGTCATCCAGCCTGCCGGCGGCGGCACCGTTCGTC
GCTCGGCCAAGTCCTCGACAACCTCCTCTCCTCGGAGTGGGGGCTCGGGGTAAAAGAACC
CACGACGCCTAAGGCGTCAAGGAACACTGTGCCTAGCCCGGGGACGCGGACGGCTTGCTG
GCCGCCCCCCGTGCTGCAATGCTATTTAATCCACACGACTCTCGGCAACGGATATCTCGG

CTCTCGCATCGATGAAGAACGTAGCGAAATGCGATACCTGGTGTGAATTGCAGAATCCCG
CGAACTATCGAGTCTTTGAACGCAAGTTGCGCCCGAGGCCATTCGGCTGAGGGCACGCCT
GCCTGGGCGTCACGCAAAACACGCTCCCACACCCTCATCGGGTAGCGGGACGCGGCATGT
GGTCCCCCGTAGCGCAAGCTGCGGTGGGCCGAAGATATGGCTGCCGGCGTATCGTGCCGG
ACACAGCGCGTGGTGGGCGACCTCGCTTTACTTACCGCAGTGCATCCGGGGCGTAGCCGA
CGTGATGGCCTCTAAGGACCCTATGAACGGTGCGCACGACG
>ITS.405_4_C9Frc
GGAAGTAAAAGTCGTAACAAGGTTTCCGTAGGTGAACCTGCGGAAGGATCATTGTCGTGA
CCCTGACCAAAATAGACCGTGCACGCGTCATCCAGCCTGCCGGCGGCGGCACCGTTCGTC
GCTCGGCCAAGTCCTCGACAACCTCCTCTCCTCGGAGTGGGGGCTCGGGGTAAAAGAACC
CACGACGCCTAAGGCGTCAAGGAACACTGTGCCTAGCCCGGGGACGCGGACGGCTTGCTG
GCCGCCCCCCGTGCTGCAATGCTATTTAATCCACACGACTCTCGGCAACGGATATCTCGG
CTCTCGCATCGATGAAGAACGTAGCGAAATGCGATACCTGGTGTGAATTGCAGAATCCCG
CGAACCATCGAGTCTTTGAACGCAAGTTGCGCCCGAGGCCATTCGGTTGAGGGCACGCCT
GCCTGGGCGTCACGCAAAACACGCTCCCACACCCTCATCGGGTAGCGGGACGCGGCATGT
GGTCCCCCGTAGCGCAAGCTGCGGTGGGCCGAAGATATGGCTGCCGGCGTATCGTGCCGG
ACACAGCGCGTGGTGGGCGACCTCGCTTTACTTACCGCAGTGCATCCGGGGCGTAGCCGA
CGTGATGGCCTCTAAGGACCCTTTGAACGGTGCGCACGACG
>ITS.M543_C1R
GGAAGTAAAAGTCGTAACAAGGTTTCCGTAGGTGAACCTGCGGAAGGATCATTGTCGTGA
CCCTGACCAAAATAGACCGTGCACGCGTCATCCAGCCTGCCGGCGGCGGCACCGTTCGTC
GCTCGGCCAAGTCCTCGACAACCTCCTCTCCTCGGAGTGGGGGCTCGGGGTAAAAGAACC
CACGACGCCTAAGGCGTCAAGGAACACTGTGCCTAGCTCGGGGACGCGGACGGCTTGCTG
GCCGCCCCTCGTGCTGCAATGCTATTTAATCCACACGACTCTCGGCAACGGATATCTCGG
CTCTCGCATCGATGAAGAACGTAGCGAAATGCGATACCTGGTGTGAATTGCAGAATCCCG
CGAACCATCGAGTCTTTGAACGCAAGTTGCGCCCGAGGCCATTCGGCTGAGGGCACGCCT
GCCTGGGCGTCACGCAAAACACGCTCCCACACCCTCATCGGGTAGCGGGACGCGGCATGT
GGTCCCCCGTAGCGCAAGCTGCGGTGGGCCGAAGATATGGCTGCCGGCGTATCGTGCCGG
ACACAGCGCGTGGTGGGCGACCTCGCTTTACTTACCGCAGTGCATCCGGGGCGTAGCCGA
CGTGATGGCCTCTAAGGACCCTATGAACGGTGCGCACGACG
>ITS.M543_C2R
GGAAGTAAAAGTCGTAACAAGGTTTCCGTAGGTGAACCTGCGGAAGGATCATTGTCGTGG
CCCTGACCAAAATAGACCGTGCACGCGTCATCCAGCCTGCCGGCGGCGGCACCGTTCGTC
GCTCGGCCAAGTCCTCGACAACCTCCTCTCCTCGGAGTGGGGGCTCGGGGTAAAAGAACC
CACGACGCCTAAGGCGTCAAGGAACACTGTGCCTAGCCCGGGGACGCGGACGGCTTGCTG
GCCGCCCCCCGTGCTGCAATGCTATTTAATCCACACGACTCTCGGCAACGGATATCTCGG
CTCTCGCATCGATGAAGAACGTAGCGAAATGCGATACCTGGTGTGAATTGCAGAATCCCG
CGAACCATCGAGTCTTTGAACGCAAGTTGCGCCCGAGGCCATTCGGCTGAGGGCACGCCT
GCCTGGGCGTCACGCGAAACACGCTCCCACACCCTCATCGGGTAGCGGGACGCGGCATGT
GGTCCCCCGTAGCGCAAGCTGCGGTGGGCCGAAGATATGGCTGCCGGCGTATCGTGCCGG
ACACAGCGCGTGGTGGGCGACCTCGCTTTACTTACCGCAGTGCATCCGGGGCGTAGCCGA
CGTGATGGCCTCTAAGGACCCTTTGAACGGTGCGCACGACG
>ITS.M543_C3R
GGAAGTAAAAGTCGTAACAAGGTTTCCGTAGGTGAACCTGCGGAAGGATCATTGTCGTGA
CCCTGACCAAAATAGACCGTGCACGCGTCATCCAGCCTGCCGGCGGCGGCACCGTTCGTC
GCTCGGCCAAGTCCTCGACAACCTCCTCTCCACGGAGTGGGGGCTCGGGGTAAAAGAACC
CACGACGCCTAAGGCGTCAAGGAACACTGTGCCTAGCCCGGGGACGCGGACGGCTTGCTG
GCCGCCCCCCGTGCTGCAATGCTATTTAATCCACACGACTCTCGGCAACGGATATCTCGG
CTCTCGCATCGATGAAGAACGTAGCGAAATGCGATACCTGGTGTGAATTGCAGAATCCCG
CGAACCATCGAGTCTTTGAACGCAAGTTGCGCCCGAGGCCATTCGGCTGAGGGCACGCCT
GCCTGGGCGTCACGCAAAACACGCTCCCACACCCTCATCGGGTAGCGGGACGCGGCATGT
GGTCCCCCGTAGCGCAAGCTGCGGTGGGCCGAAGATATGGCTGCCGGCGTATCGTGCCGG
ACACAGCGCGTGGTGGGCGACCTCGCTTTACTTACCGCAGTGCATCCGGGGCGTAGCCGA
CGTGATGGCCTCTAAGGACCCTTTGAACGGTGCGCACGACG

>ITS.M543_C4Frc_rev
GGAAGTAAAAGTCGTAACAAGGTTTCCGTAGGTGAACCTGCGGAAGGATCATTGTCGTGA
CCCTGACCAAAATAGACCGTGCACGCGTCATCCAGCCTGCCGGCGGCGGCACCGTTCGTC
GCTCGGCCAAGTCCTCGACAACCTCCTCTCCTCGGAGTGGGGGCTCGGGGTAAAAGAACC
CACGACGCCTAAGGCGTCAAGGAACACTGTGCCTAGCCCGGGGACGCGGACGGCTTGCTG
GCCGCCCCCCGTGCTGCAATGCTATTTAATCCACACGACTCTCGGCAACGGATATCTCGG
CTCTCGCATCGATGAAGAACGTAGCGAAATGCGATACCTGGTGTGAATTGCAGAATCCCG
CGAACCATCGAGTCTTTGAACGCAAGTTGCGCCCGAGGCCATTCGGCTGAGGGCACGCCT
GCCTGGGCGTCACGCAAAACACGCTCCCACACCCTCATCGGGTAGCGGGACGCGGCATGT
GGTCCCCCGTAGCGCAAGCTGCGGTGGGCCGAAGATATGGCTGCCGGCGTATCGTGCCGG
ACACAGCGCGTGGTGGGCGACCTCGCTTTACTTACCGCAGTGCATCCGGGGCGTAGCCGA
CGTGATGGCCTCTAAGGACCCTATGAACGGTGCGCACGACG
>ITS.M543_C5F
GGAAGTAAAAGTCGTAACAAGGTTTCCGTAGGTGAACCTGCGGAAGGATCATTGTCGTGA
CCCTGACCAAAATAGACCGTGCACGCGTCATCCAGCCTGCCGGCGGCGGCACCGTTCGTC
GCTCGGCCAAGTCCTCGACAACCTCCTCTCCTCGGAGTGGGGGCTCGGGGTAAAAGAACC
CACGACGCCTAAGGCGTCAAGGAACACTGTGCCTAGCCCGGGGACGCGGACGGCTTGCTG
GCCGCCCCCCGTGCTGCAATGCTATTTAATCCACACGACTCTCGGCAACGGATATCTCGG
CTCTCGCATCGATGAAGAACGTAGCGAAATGCGATACCTGGTGTGAATTGCGGAATCCCG
CGAACCATCGAGTCTTTGAACGCAAGTTGCGCCCGAGGCCATTCGGCTGAGGGCACGCCT
GCCTGGGCGTCACGCAAAACACGCTCCCACACCCTCATCGGGTAGCGGGACGCGGCTTGT
GTTTCCCCGTAGCGCAAGCTGCGGTGGGCCGAAGATATGGCTGCCGGCGTATCGTGCCGG
ACACAGCGCGTGGTGGGCGACCTCGCTTTACTTACCGCAGTGCATCCGGGGCGTAGCCGA
CGTGATGGCCTCTAAGGACCCTTTGAACGGTGCGCACGACG
>ITS.M543_C6F
GGAAGTAAAAGTCGTAACAAGGTTTCCGTAGGTGAACCTGCGGAAGGATCATTGTCGTGA
CCCTGACCAAAATAGACCGTGCACGCGTCATCCAGCCTGCCGGCGGCGGCACCGTTCGTC
GCTCGGCCAAGTCCTCGACAACCTCCTCTCCTCGGAGTGGGGGCTCGGGGTAAAAGAACC
CACGACGCCTAAGGCGTCAAGGAACACTGTGCCTAGCCCGGGGACGCGGACGGCTTGCTG
GCCGCCCCCCGTGCTGCAATGCTATTTAATCCACACGACTCTCGGCAACGGATATCTCGG
CTCTCGCATCGATGAAGAACGTAGCGAAATGCGATACCTGGTGTGAATTGCAGAATCCCG
CGAACCATCGAGTCTTTGAACGCAAGTTGCGCCCGAGGCCATTCGGCTGAGGGCACGCCT
GCCTGGGCGTCACGCAAAACACGCTCCCACACCCTCATCGGGTAGCGGGACGCGGCATGT
GGTCCCCCGTAGCGCAAGCTGCGGTGGGCCGAAGATATGGCTGCCGGCGTATCGTGCCGG
ACACAGCGCGTGGTGGGCGACCTCGCTTTACTTACCGCAGTGCATCCGGGGCGTAGCCGA
CGTGATGGCCTCTAAGGACCCTATGAACGGTGCGCACGACG
>ITS.M543_C7Frc_rev
GGAAGTAAAAGTCGTAACAAGGTTTCCGTAGGTGAACCTGCGGAAGGATCATTGTCGTGA
CCCTGACCAAAATAGACCGTGCACGCGTCATCCAGCCTGCCGGCGGCGGCACCGTTCGTC
GCTCGGCCAAGTCCTCGACAACCTCCTCTCCTCGGAGTGGGGGCTCGGGGTAAAAGAACC
CACGACGCCTAAGGCGTCAAGGAACACTGTGCCTAGCCCGGGGACGCGGACGGCTTGCTG
GCCGCCCCCCGTGCTGCAATGCTATTTAATCCACACGACTCTCGGCAACGGATATCTCGG
CTCTCGCATCGATGAAGAACGTAGCGAAATGCGATACCTGGTGTGAATTGCAGAATCCCG
CGAACCATCGAGTCTTTGAACGCAAGTTGCACCCGAGGCCATTCGGCTGAGGGCACGCCT
GCCTGGGCGTCACGCAAAACACGCTCCCACACCCTCATCGGGTAGCGGGACGCGGCTTGT
GGTTCCCCGTAGCGCAAGCTGCGGTGGGCCGAAGATATGGCTGCCGGCGTATCGTGCCGG
ACACAGCGCGTGGTGGGCGACCTCGCTTTACTTACCGCAGTGCATCCGGGGCGTAGCCGA
CGTGATGGCCTCTAAGGACCCTATGAACGGTGCGCACGACG
>ITS.M543_C8F
GGAAGTAAAAGTCGTAACAAGGTTTCCGTAGGTGAACCTGCGGAAGGATCATTGTCGTGA
CCCTGACCAAAATAGACCGTGCACGCGTCATCCAGCCTGCCGGCGGCGGCACCGTTCGTC
GCTCGGCCAAGTCCTCGACAACCTCCTCTCCACGGAGTGGGGGCTCGGGGTAAAAGAACC
CACGACGCCTAAGGCGTCAAGGAACACTGTGCCTAGCCCGGGGACGCGGACGGCTTGCTG
GCCGCCCCCCGTGCTGCAATGCTATTTAATCCACACGACTCTCGGCAACGGATATCTCGG

CTCTCGCATCGATGAAGAACGTAGCGAAATGCGATACCTGGTGTGAATTGCAGAATCCCG
CGAACCATCGAGTCTTTGAACGCAAGTTGCGCCCGAGGCCATTCGGCTGAGGGCACGCCT
GCCTGGGCGTCACGCAAAACACGCTCCCACACCCTCATCGGGTAGCGGGACGCGGCATGT
GGTCCCCCGTAGCGCAAGCTGCGGTGGGCCGAAGATATGGCTGCCGGCGTATCGTGCCGG
ACACAGCGCGTGGTGGGCGACCTCGCTTTACTTACCGCAGTGCATCCGGGGCGTAGCCGA
CGTGATGGCCTCTAAGGACCCTATGAACGGTGCGCACGACG
>ITS.M627_C1F
GGAAGTAAAAGTCGTAACAAGGTTTCCGTAGGTGAACCTGCGGAAGGATCATTGTCGTGA
CCCTGACCAAAATAGACCGTGCACGCGTCATCCAGCCTGCCGGCGGCGGCACCGTTCGTC
GCTCGGCCAAGTCCTCGACAACCTCCTCTCCTCGGAGTGGGGGCTCGGGGTAAAAGAACC
CACGACGCCTAAGGCGTCAAGGAACACTGTGCCTAGCCCGGGGACGCGGACGGCTTGCTG
GCCGCCCCCCGTGCTGCAATGCTATTTAATCCACACGACTCTCGGCAACGGATATCTCGG
CTCTCGCATCGATGAAGAACGTAGCGAAATGCGATACCTGGTGTGAATTGCAGAATCCCG
CGAACCATCGAGTCTTTGAACGCAAGTTGCGCCCGAGGCCATTCGGCTGAGGGCACGCCT
GCCTGGGCGTCACGCAAAACACGCTCCCACACCCTCATCGGGTAGCGGGACGCGGCTTGT
GGTTCCCCGTAGCGCAAGCTGCGGTGGGCCGAAGATATGGCTGCCGGCGTATCGTGCCGG
ACACAGCGCGTGGTGGGCGACCTCGCTTTACTTACCGCAGTGCATCCGGGGCGTAGCCGA
CGTGATGGCCTCTAAGGACCCTATGAACGGTGCGCACGACG
>ITS.M627_C2F
GGAAGTAAAAGTCGTAACAAGGTTTCCGTAGGTGAACCCGCGGAAGGATCATTGTCGTGA
CCCTGACCAAAATAGACCGTGCACGCGTCATCCAGCCTGCCGGCGGCGGCACCGTTCGTC
GCTCGGCCAAGTCCTCGACAACCTCCTCTCCTCGGAGTGGGGGCTCGGGGTAAAAGAACC
CACGACGCCTAAGGCGTCAAGGAACACTGTGCCTAGCCCGGGGACGCGGACGGCTTGCTG
GCCGCCCCCCGTGCTGCAATGCTATTTAATCCACACGACTCTCGGCAACGGATATCTCGG
CTCTCGCATCGATGAAGAACGTAGCGAAATGCGATACCTGGTGTGAATTGCAGAATCCCG
CGAACCATCGAGTCTTTGAACGCAAGTTGCGCCCGAGGCCATTCGGCTGAGGGCACGCCT
GCCTGGGCGTCACGCAAAACACGCTCCCACACCCTCATCGGGTAGCGGGACGCGGCATGT
GGTCCCCCGTAGCGCAAGCTGCGGTGGGCCGAAGATATGGCTGCCGGCGTATCGTGCCGG
ACACAGCGCGTGGTGGGCGACCTCGCTTTACTTACCGTAGTGCATCCGGGGCGTAGCCGA
CGTGATGGCCTCTAAGGACCCTATGAACGGTGCGCACGACG
>ITS.M627_C3R
GGAAGTAAAAGTCGTAACAAGGTTTCCGTAGGTGAACCTGCGGAAGGATCATTGTCGTGA
CCCTGACCAAAATAGACCGTGCACGCGTCATCCAGCCTGCCGGCGGCGGCACCGTTCGTC
GCTCGGCCAAGTCCTCGACAACCTCCTCTCCTCGGAGTGGGGGCTCGGGGTAAAAGAACC
CACGACGCCTAAGGCGTCAAGGAACACTGTGCCTAGCCCGGGGACGCGGACGGCTTGCTG
GCCGCCCCCCGTGCTGCAATGCTATTTAATCCACACGACTCTCGGCAACGGATATCTCGG
CTCTCGCATCGATGAAGAACGTAGCGAAATGCGATACCTGGTGTGAATTGCAGAATCCCG
CGAACCATCGAGTCTTTGAACGCAAGTTGCGCCCGAGGCCATTCGGCTGAGGGCACGCCT
GCCTGGGCGTCACGCAAAACACGCTCCCACACCCTCATCGGGTAGCGGGACGCGGCTTGT
GGTTCCCCGTAGCGCAAGCTGCGGTGGGCCGAAGATATGGCTGCCGGCGTATCGTGCCGG
ACACAGCGCGTGGTGGGCGACCTCGCTTTACTTACCGCAGTGCATCCGGGGCGTAGCCGA
CGTGATGGCCTCTAAGGACCCTTTGAACGGTGCGCACGACG
>ITS.M627_C4F
GGAAGTAAAAGTCGTAACAAGGTTTCCGTAGGTGAACCTGCGGAAGGATCATTGTCGTGA
CCCTGACCAAAATAGACCGTGCACGCGTCATCCAGCCTGCCGGCGGCGGCACCGTTCGTC
GCTCGGCCAAGTCCTCGACAACCTCCTCTCCTCGGAGTGGGGGCTCGGGGTAAAAGAACC
CACGATGCCTAAGGCGTCAAGGAACACTGTGCCTAGCCCGGGGACGCGGACGGCTTGCTG
GCCGCCCCCCGTGCTGCAATGCTATTTAATCCACACGACTCTCGGCAACGGATATCTCGG
CTCTCGCATCGATGAAGAACGTAGCGAAATGCGATACCTGGTGTGAATTGCAGAATCCCG
CGAACCATCGAGTCTTTGAACGCAAGTTGCGCCCGAGGCCATTCGGCTGAGGGCACGCCT
GCCTGGGCGTCACGCAAAACACGCTCCCACACCCTCATCGGGTAGCGGGACGCGGCATGT
GGTCCCCCGTAGCGCAAGCTGCGGTGGGCCGAAGATATGGCTGCCGGCGTACCGTGCCGG
ACACAGCGCGTGGTGGGCGACCTCGCTTTACTTACCGCAGTGCATCCGGGGCGTAGCCGA
CGTGATGGCCTCTAAGGACCCTATGAACGGTGCGCACGACG

>ITS.M627_C5Frc_rev
GGAAGTAAAAGTCGTAACAAGGTTTCCGTAGGTGAACCTGCGGAAGGATCATTGTCGTGA
CCCTGACCAAAATAGACCGTGCACGCGTCATCCAGCCTGCCGGCGGCGGCACCGTTCGTC
GCTCGGCCAAGTCCTCGACAACCTCCTCTCCACGGAGTGGGGGCTCGGGGTAAAAGAACC
CACGACGCCTAAGGCGTCAAGGAACACTGTGCCTAGCCCGGGGACGCGGACGGCTTGCTG
GCCGCCCCCCGTGCTGCAATGCTATTTAATCCACACGACTCTCGGCAACGGATATCTCGG
CTCTCGCACCGATGAAGAACGTAGCGAAATGCGATACCTGGTGTGAATTGCAGAATCCCG
CGAACCATCGAGTCTTTGAACGCAAGTTGCGCCCGAGGCCATTCGGCTGAGGGCACGCCT
GCCTGGGCGTCACGCAAAACACGCTCCCACACCCTCATCGGGTAGCGGGACGCGGCATGT
GGTCCCCCGTAGCGCAAGCTGCGGTGGGCCGAAGATATGGCTGCCGGCGTATCGTGCCGG
ACACAGCGCGTGGTGGGCGACCTCGCTTTACTTACCGCAGTGCATCCGGGGCGTAGCCGA
CGTGATGGCCTCTAAGGACCCTATGAACGGTGCGCACGACG
>ITS.M627_C6Frc_rev
GGAAGTAAAAGTCGTAACAAGGTTTCCGTAGGTGAACCTGCGGAAGGATCATTGTCGTGA
CCCTGACCAAAATAGACCGTGCACGCGTCATCCAGCCTGCCGGCGGCGGCACCGTTCGTC
GCTCGGCCAAGTCCTCGACAACCTCCTCTCCTCGGAGTGGGGGCTCGGGGTAAAAGAACC
CACGACGCCTAAGGCGTCAAGGAACACTGTGCCTAGCCCGGGGACGCGGACGGCTTGCTG
GCCGCCCCCCGTGCTGCAATGCTATTTAATCCACACGACTCTCGGCAACGGATATCTCGG
CTCTCGCATCGATGAAGAACGTAGCGAAATGCGATACCTGGTGTGAATTGCAGAATCCCG
CGAACCATCGAGTCTTTGAACGCAAGTTGCGCCCGAGGCCATTCGGCTGAGGGCACGCCT
GCCTGGGCGTCACGCAAAACACGCTCCCACACCCTCATCGGGTAGCGGGACGCGGCATGT
GGTCCCCCGTAGCGCAAGCTGCGGTGGGCCGAAGATATGGCTGCCGGCGTATCGTGCCGG
ACACAGCGCGTGGTGGGCGACCTCGCTTTACTTACCGCAGTGCATCCGGGGCGTAGCCGA
CGTGATGGCCTCTAAGGACCCTATGAACGGTGCGCACGACG
>ITS.M627_C7Frc_rev
GGAAGTAAAAGTCGTAACAAGGTTTCCGTAGGTGAACCTGCGGAAGGATCATTGTCGTGA
CCCTGACCAAAATAGACCGTGCACGCGTCATCCAGCCTGCCGGCGGCGGCACCGTTCGTC
GCTCGGCCAAGTCCTCGACAACCTCCTCTCCTCGGAGTGGGGGCTCGGGGTAAAAGAACC
CACGACGCCTAAGGCGTCAAGGAACACTGTGCCTAGCCCGGGGACGCGGACGGCTTGCTG
GCCGCCCCCCGTGCTGCAATGCTATTTAATCCACACGACTCTCGGCAACGGATATCTCGG
CTCTCGCATCGATGAAGAACGTAGCGAAATGCGATACCTGGTGTGAATTGCAGAATCCCG
CGAACCATCGAGTCTTTGAACGCAAGTTGCGCCCGAGGCCATTCGGCTGAGGGCACGCCT
GCCTGGGCGTCACGCAAAACACGCTCCCACACCCTCATCGGGTAGCGGGACGCGGCATGT
GGTCCCCCGTAGCGCAAGCTGCGGTGGGCCGAAGATATGGCTGCCGGCGTATCGTGCCGG
ACACAGCGCGTGGTGGGCGACCTCGCTTTACTTACCGCAGTGCATCCGGGGCGTAGCCGA
CGTGATGGCCTCTAAGGACCCTTTGAACGGTGCGCACGACG
>ITS.M627_C8Frc_rev
GGAAGTAAAAGTCGTAACAAGGTTTCCGTAGGTGAACCTGCGGAAGGATCATTGTCGTGA
CCCTGACCAAAATAGACCGTGCACGCGTCATCCAGCCTGCCGGCGGCGGCACCGTTCGTC
GCTCGGCCAAGTCCTCGACAACCTCCTCTCCACGGAGTGGGGGCTCGGGGTAAAAGAACC
CACGACGCCTAAGGCGTCAAGGAACACTGTGCCTAGCCCGGGGACGCGGACGGCTTGCTG
GCCGCCCCCCGTGCTGCAATGCTATTTAATCCACACGACTCTCGGCAACGGATATCTCGG
CTCTCGCATCGATGAAGAACGTAGCGAAATGCGATACCTGGTGTGAATTGCAGAATCCCG
CGAACCATCGAGTCTTTGAACGCAGGTTGCGCCCGAGGCCATTCGGCTGAGGGCACGCCT
GCCTGGGCGTCACGCAAAACACGCTCCCACACCCTCATCGGGTAGCGGGACGCGGCTTGT
GGTTCCCCGTAGCGCAAGCTGCGGTGGGCCGAAGATATGGCTGCCGGCGTATCGTGCCGG
ACACAGCGCGTGGTGGGCGACCTCGCTTTACTTACCGCAGTGCATCCGGGGCGTAGCCGA
CGTGATGGCCTCTAAGGACCCTTTGAACGGTGCGCACGACG
>ITS.M647_C1F
GGAAGTAAAAGTCGTAACAAGGTTTCCGTAGGTGAACCTGCGGAAGGATCATTGTCGTGA
CCCTGACCAAAATAGACCGTGCACGCGTCATCCAGCCTGCCGGCGGCGGCACCGTTCGTC
GCTCGGCCAAGTCCTCGACAACCTCCTCTCCTCGGAGTGGGGGCTCGGGGTAAAAGAACC
CACGACGCCTAAGGCGTCAAGGAACACTGTGCCTAGCCCGGGGACGCGGACGGCTTGCTG
GCCGCCCCCCGTGCTGCAATGCTATTTAATCCACACGACTCTCGGCAACGGATATCTCGG

CTCTCGCATCGATGAAGAACGTAGCGAAATGCGATACCTGGTGTGAATTGCAGAATCCCG
CGAACCATCGAGTCTTTGAACGCAAGTTGCGCCCGAGGCCATTCGGTTGAGGGCACGCCT
GCCTGGGCGTCACGCAAAACACGCTCCCACACCCTCATCGGGTAGCGGGACGCGGCATGT
GGTCCCCCGTAGCGCAAGCTGCGGTGGGCCGAAGATATGGCTGCCGGCGTATCGTGCCGG
ACACAGCGCGTGGTGGGCGACCTCGCTTTACTTACCGCAGTGCATCCGGGGCGTAGCCGA
CGTGATGGCCTCTAAGGACCCTATGAACGGTGCGCACGACG
>ITS.M647_C2R
GGAAGTAAAAGTCGTAACAAGGTTTCCGTAGGTGAACCTGCGGAAGGATCATTGTCGTGA
CCCTGACCAAAATAGACCGTGCACGCGTCATCCACCCTGCCGGCGGCGGCACCGTTCGTC
GCTCGGCCAAGTCCTCGACAACCTCCTCTCCTCGGAGTGGGGGCTCGGGGTAAAAGAACC
CACGACGCCTAAGGCGTCAAGGAACACTGTGCCTAGCCCGGGGACGCGGACGGCTTGCTG
GCCGCCCCCCGTGCTGCAATGCTATTTAATCCACACGACTCTCGGCAACGGATATCTCGG
CTCCCGCATCGATGAAGAACGTAGCGAAATGCGATACCTGGTGTGAATTGCAGAATCCCG
CGAACCATCGAGTCTTTGAACGCAAGTTGCGCCCGAGGCCATTCGGCTGAGGGCACGCCT
GCCTGGGCGTCACGCAAAACACGCTCCCACACCCTCATCGGGTAGCGGGACGCGGCATGT
GGTCCCCCGTAGCGCAAGCTGCGGTGGGCCGAAGATATGGCTGCCGGCGTATCGTGCCGG
ACACAGCGCGTGGTGGGCGACCTCGCTTTACTTACCGCAGTGCATCCGGGGCGTAGCCGA
CGTGATGGCCTCTAAGGACCCTTTGAACGGTGCGCACGACG
>ITS.M647_C3R
GGAAGTAAAAGTCGTAACAAGGTTTCCGTAGGTGAACCTGCGGAAGGATCATTGTCGTGA
CCCTGACCAAAATAGACCGTGCACGCGTCATCCAGCCTGCCGGCGGCGGCACCGTTCGTC
GCTCGGCCAAGTCCTCGACAACCTCCTCTCCTCGGAGTGGGGGCTCGGGGTAAAAGAACC
CACGACGCCTAAGGCGTCAAGGAACACTGTGCCTAGCCCGGGGACGCGGACGGCTTGCTG
GCCGCCCCCCGTGCTGCAATGCTATTTAATCCACACGACTCTCGGCAACGGATATCTCGG
CTCTCGCATCGATGAAGAACGTAGCGAAATGCGATACCTGGTGTGAATTGCAGAATCCCG
CGAACCATCGAGTCTTTGAACGCAAGTTGCGCCCGAGGCCATTCGGCTGAGGGCACGCCT
GCCTGGGCGTCACGCAAAACACGCTCCCACACCCTCATCGGGTAGCGGGACGCGGCATGT
GGTCCCCCGTAGCGCAAGCTGCGGTGGGCCGAAGATATGGCTGCCGGCGTATCGTGCCGG
ACACAGCGCGTGGTGGGCGACCTCGCTTTACTTACCGCAGTGCATCCGGGGCGTAGCCGA
CGTGATGGCCTCTAAGGACCCTATGAACGGTGCGCACGACG
>ITS.M647_C4Frc_rev
GGAAGTAAAAGTCGTAACAAGGTCTCCGTAGGTGAACCTGCGGAAGGATCATTGTCGTGA
CCCTGACCAAAATAGACCGTGCACGCGTCATCCAGCCTGCCGGCGGCGGCACCGTTCGTC
GCTCGGCCAAGTCCTCGACAACCTCCTCTCCTCGGAGTGGGGGCTCGGGGTAAAAGAACC
CACGACGCCTAAGGCGTCAAGGAACACTGTGCCTAGCCCGGGGACGCGGACGGCTTGCTG
GCCGCCCCCCGTGCTGCAATGCTATTTAATCCACACGACTCTCGGCAACGGATATCTCGG
CTCTCGCATCGATGAAGAACGTAGCGAAATGCGATACCTGGTGTGAATTGCAGAATCCCG
CGAACCATCGAGTCTTTGAACGCAAGTTGCGCCCGAGGCCATTCGGTTGAGGGCACGCCT
GCCTGGGCGTCACGCAAAACACGCTCCCACACCCTCATCGGGTAGCGGGACGCGGCATGT
GGTCCCCCGTAGCGCAAGCTGCGGTGGGCCGAAGATATGGCTGCCGGCGTATCGTGCCGG
ACACAGCGCGTGGTGGGCGACCTCGCTTTACTTACCGCAGTGCATCCGGGGCGTAGCCGA
CGTGATGGCCTCTAAGGACCCTTTGAACGGTGCGCACGACG
>ITS.M647_C5F
GGAAGTAAAAGTCGTAACAAGGTTTCCGTAGGTGAACCTGCGGAAGGATCATTGTCGTGA
CCCTGACCAAAATAGACCGTGCACGCGTCATCCAGCCTGCCGGCGGCGGCACCGTTCGTC
GCTCGGCCAAGTCCTCGACAACCTCCTCTCCTCGGAGTGGGGGCTCGGGGTAAAAGAACC
CACGACGCCTAAGGCGTCAAGGAACACTGTGCCTAGCCCGGGGACGCGGACGGCTTGCTG
GCCGCCCCCCGTGCTGCAATGCTATTTAATCCACACGACTCTCGGCAACGGATATCTCGG
CTCTCGCATCGATGAAGAACGTAGCGAAATGCGATACCTGGTGTGAATTGCAGAATCCCG
CGAACCATCGAGTCTTTGAACGCAAGTTGCGCCCGAGGCCATTCGGCTGAGGGCACGCCT
GCCTGGGCGTCACGCAAAACACGCTCCCACACCCTCATCGGGTAGCGGGACGCGGCATGT
GGTCCCCTGTAGCGCAAGCTGCGGTGGGCCGAAGATATGGCTGCCGGCGTATCGTGCCGG
ACGCAGCGCGTGGTGGGCAACCTCGCTTTACTTACCGCGGTGCATCTGGGGCGTAGCCGA
CGTGATGGCCTCTAAGGACCCTATGAACGGTGCGCACGACG

>ITS.M647_C6F
GGAAGTAAAAGTCGTAACAAGGTTTCCGTAGGTGAACCTGCGGAAGGATCATTGTCGTGA
CCCTGACCAAAATAGACCGTGCACGCGTCATCCAGCCTGCCGGCGGCGGCACCGTTCGTC
GCTCGGCCAAGTCCTCGACAACCTCCTCTCCTCGGAGTGGGGGCTCGGGGTAAAAGAACC
CACGACGCCTAAGGCGTCAAGGAACACTGTGCCTAGCCCGGGGACGCGGACGGCTTGCTG
GCCGCCCCCCGTGCTGCAATGCTATTTAATCCACACGACTCTCGGCAACGGATATCCCGG
CTCTCGCATCGATGAAGAACGTAGCGAAATGCGATACCTGGTGTGAATTGCAGAATCCCG
CGAACCATCGAGTCTTTGAACGCAAGTTGCGCCCGAGGCCATTCGGCTGAGGGCACGCCT
GCCTGGGCGTCACGCAAAACACGCTCCCACACCCTCATCGGGTAGCGGGACGCGGCATGT
GGTCCCCCGTAGCGCAAGCTGCGGTGGGCCGAAGATATGGCTGCCGGCGTATCGTGCCGG
ACACAGCGCGTGGTGGGCGACCTCGCTTTACTTACCGCAGTGCATCCGGGGCGTAGCCGA
CGTGATGGCCTCTAAGGACCCTTTGAACGGTGCGCACGACG
>ITS.M647_C7F
GGAAGTAAAAGTCGTAACAAGGTTTCCGTAGGTGAACCTGCGGAAGGATCATTGTCGTGA
CCCTGACCAAAATAGACCGTGCACGCGTCATCCAGCCTGCCGGCGGCGGCACCGTTCGTC
GCTCGGCCAAGTCCTCGACAACCTCCTCTCCTCGGAGTGGGGGCTCGGGGTAAAAGAACC
CACGACGCCTAAGGCGTCAAGGAACACTGTGCCTAGCCCGGGGACGCGGACGGCTTGCTG
GCCGCCCCCCGTGCTGCAATGCTATTTAATCCACACGACTCTCGGCAACGGATATCTCGG
CTCTCGCATCGATGAAGAACGTAGCGAAATGCGATACCTGGTGTGAATTGCAGAATCCCG
CGAACCATCGAGTCTTTGAACGCAAGTTGCGCCCGAGGCCATTCGGCTGAGGGCACGCCT
GCCTGGGCGTCACGCAAAACACGCTCCCACACCCTCATCGGGTAGCGGGACGCGGCATGT
GGTCCCCCGTAGCGCAAGCTGCGGTGGGCCGAAGATATGGCTGCCGGCGTATCGTGCCGG
ACACAGCGCGTGGTGGGCGACCTCGCTTTACTTACCGCAGTGCATCCGGGGCGTAGCCGA
CGTGATGGCCTCTAAGGACCCTTTGAACGGTGCGCACGACG
>ITS.M647_C8F
GGAAGTAAAAGTCGTAACAAGGTTTCCGTAGGTGAACCTGCGGAAGGATCATTGTCGTGA
CCCTGACCAAAATAGACCGTGCACGCGTCATCCAGCCTGCCGGCGGCGGCACCGTTCGTC
GCTCGGCCAAGTCCTCGACAACCTCCTCTCCTCGGAGTGGGGGCTCGGGGTAAAAGAACC
CACGACGCCTAAGGCGTCAAGGAACACTGTGCCTAGCCCGGGGACGCGGACGGCTTGCTG
GCCGCCCCCCGTGCTGCAATGCTATTTAATCCACACGACTCTCGGCAACGGATATCTCGG
CTCTCGCATCGATGAAGAACGTAGCGAAATGCGATACCTGGTGTGAATTGCAGAATCCCG
CGAACCATCGAGTCTTTGAACGCAAGTTGCGCCCGAGGCCATTCGGCTGAGGGCACGCCT
GCCTGGGCGTCACGCAAAACACGCTCCCACACCCTCATCGGGTAGCGGGACGCGGCATGT
GGTCCCCCGTAGCGCAAGCTGCGGTGGGCCGAAGATATGGCTGCCGGCGTATCGTGCCGG
ACACAGCGCGTGGTGGGCGACCTCGCTTTACTTACCGCAGTGCATCCGGGGCGTAGCCGA
CGTGATGGCCTCTAAGGACCCTTTGAACGGTGCGCACGACG
>ITS.M820_C1R
GGAAGTAAAAGTCGTAACAAGGTTTCCGTAGGTGAACCTGCGGAAGGATCATTGTCGTGA
CCCTGACCAAAATAGACCGTGCACGCGTCATCCAGCCTGCCGGCGGCGGCACCGTTCGTC
GCTCGGCCAAGTCCTCGACAACCTCCTCTCCTCGGAGTGGGGGCTCGGGGTAAAAGAACC
CACGACGCCTAAGGCGTCAAGGAACACTGTGCCTAGCCCGGGGACGCGGACGGCTTGCTG
GCCGCCCCCCGTGCTGCAATGCTATTTAATCCACACGACTCTCGGCAACGGATATCTCGG
CTCTCGCATCGATGAAGAACGTAGCGAAATGCGATACCTGGTGTGAATTGCAGAATCCCG
CGAACCATCGAGTCTTTGAACGCAAGTTGCGCCCGAGGCCATTCGGCTGAGGGCACGCCT
GCCTGGGCGTCACGCAAAACACGCTCCCACACCCTCATCGGGTAGCGGGACGCGGCATGT
GGTCCCCCGTAGCGCAAGCTGCGGTGGGCCGAAGATATGGCTGCCGGCGTATCGTGCCGG
ACACAGCGCGTGGTGGGCGACCTCGCTTTACTTACCGCAGTGCATCCGGGGCGTAGCCGA
CGTGATGGCCTCTAAGGACCCTTTGAACGGTGCGCACGACG
>ITS.M820_C2R
GGAAGTAAAAGTCGTAACAAGGTTTCCGTAGGTGAACCTGCGGAAGGATCATTGTCGTGA
CCCTGACCAAAATAGACCGTGCACGCGTCATCCAGCCTGCCGGTGGCGGCACCGTTCGTC
GCTCGGCCAAGTCCTCGACAACCTCCACTCCTCGGAGTGGGGGCTCGGGGTAAAAGAACC
CACGACGCCTAAGGCGTCAAGGAACACTGTGCCTAGCCCGGGGACGCGGACGGCTTGCTG
CCCGCCCCCCGTGCTGCAATGCTATTTAATCCACACGACTCTCGGCAACGGATATCTCGG

CTCTCGCATCGATGAAGAACGTAGCGAAATGCGATACCTGGTGTGAATTGCAGAATCCCG
CGAACCATCGAGTCTTTGAACGCAAGTTGCGCCCGAGGCCATTCGGCTGAGGGCACGCCT
GCCTGGGCGTCACGCAAAACACGCTCCCACACCCTCATCGGGTAGCGGGACGCGGCATGT
GGTCCCCCGTAGCGCAAGCTGCGGTGGGCCGAAGATATGGCTGCCGGCGTATCGTGCCGG
ACACAGCGCGTGGTGGGCGACCTCGCTTTACTTACCGCAGTGCATCCGGGGCGTAGCCGA
CGTGATGGCCTCTAAGGACCCTTTGAACGGTGCGCACGACG
>ITS.M820_C3F
GGAAGTAAAAGTCGTAACAAGGTTTCCGTAGGTGAACCTGCGGAAGGATCATTGTCGTGA
CCCTGACCAAAATAGACCGTGCACGCGTCATCCAGCCTGCCGGCGGCGGCACCGTTCGTC
GCTCGGCCAAGTCCTCGACAACCTCCTCTCCTCGGAGTGGGGGCTCGGGGTAAAAGAACC
CACGACGCCTAAGGCGTCAAGGAACACTGTGCCTAGCCCGGGGACGCGGACGGCTTGCTG
CCCGCCCCCCGTGCTGCAATGCTATTTAATCCACACGACTCTCGGCAACGGATATCTCGG
CTCTCGCATCGATGAAGAACGTAGCGAAATGCGATACCTGGTGTGAATTGCAGAATCCCG
CGAACCATCGAGTCTTTGAACGCAAGTTGCGCCCGAGGCCATTCGGCTGAGGGCACGCCT
GCCTGGGCGTCACGCAAAACACGCTCCTACACCCTCATCGGGTAGCGGGACGCGGCATGT
GGTCCCCCGTAGCGCAAGCTGCGGTGGGCCGAAGATATGGCTGCCGGCGTATCGTGCCGG
ACACAGCGCGTGGTGGGCGACCTCGCTTTACTTACCGCAGTGCATCCGGGGCGTAGCCGA
CGTGATGGCCTCTAAGGACCCTATGAACGGTGCGCACGACG
>ITS.M820_C4F
GGAAGTAAAAGTCGTAACAAGGTTTCCGTAGGTGAACCTGCGGAAGGATCATTGTCGTGA
CCCTGACCAAAATAGACCGTGCACGCGTCATCCACCCTGCCGGCGGCGGCACCGTTCGTC
GCTCGGCCAAGTCCTCGACAACCTCCTCTCCTCGGAGTGGGGGCTCGGGGTAAAAGAACC
CACGACGCCTAAGGCGTCAAGGAACACTGTGCCTAGCCCGGGGACGCGGACGGCTTGCTG
GCCGCCCCCCGTGCTGCAATGCTATTTAATCCACACGACTCTCGGCAACGGATATCTCGG
CTCTCGCATCGATGAAGAACGTAGCGAAATGCGATACCTGGTGTGAATTGCAGAATCCCG
CGAACCATCGAGTCTTTGAACGCAAGTTGCGCCCGAGGCCATTCGGCTGAGGGCACGCCT
GCCTGGGCGTCACGCAAAACACGCTCCCACACCCTCATCGGGTAGCGGGACGCGGCATGT
GGTCCCCCGTAGCGCAAGCTGCGGTGGGCCGAAGATATGGCTGCCGGCGTATCGTGCCGG
ACACAGCGCGTGGTGGGCGACCTCGCTTTACTTACCGCAGTGCATCCGGGGCGTAGCCGA
CGTGATGGCCTCTAAGGACCCTATGAACGGTGCGCACGANN
>ITS.M820_C5Frc_rev
GGAAGTAAAAGTCGTAACAAGGTTTCCGTAGGTGAACCTGCGGAAGGATCATTGTCGTGA
CCCTGACCAAAATAGACCGTGCACGCGTCATCCAGCCTGCCGGCGGCGGCACCGTTCGTC
GCTCGGCCAAGTCCTCGACAACCTCCTCTCCTCGGAGTGGGGGCTCGGGGTAAAAGAACC
CACGACGCCTAAGGCGTCAAGGAACACTGTGCCTAGCCCGGGGACGCGGACGGCTTGCTG
CCCGCCCCCCGTGCTGCAATGCTATTTAATCCACACGACTCTCGGCAACGGATATCTCGG
CTCTCGCATCGATGAAGAACGTAGCGAAATGCGATACCTGGTGTGAATTGCAGAATCCCG
CGAACCATCGAGTCTTTGAACGCAAGTTGCGCCCGAGGCCATTCGGCTGAGGGCACGCCT
GCCTGGGCGTCACGCAAAACACGCTCCCACACCCTCATCGGGTAGCGGGACGCGGCATGT
GGTCCCCCGTAGCGCAAGCTGCGGTGGGCCGAAGATATGGCTGCCGGCGTATCGTGCCGG
ACACAGCGCGTGGTGGGCGACCTCGCTTTACTTACCGCAGTGCATCCGGGGCGTAGCCGA
CGTGATGGCCTCTAAGGACCCTTTGAACGGTGCGCACGACG
>ITS.M820_C6Frc_rev
GGAAGTAAAAGTCGTAACAAGGTTTCCGTAGGTGAACCTGCGGAAGGATCATTGTCGTGA
CCCTGACCAAAATAGACCGTGCACGCGTCATCCAGCCTGCCGGCGGCGGCACCGTTCGTC
GCTCGGCCAAGTCCTCGACAACCTCCTCTCCACGGAGTGGGGGCTCGGGGTAAAAGAACC
CACGACGCCTAAGGCGTCAAGGAACACTGTGCCTAGCCCGGGGACGCGGACGGCTTGCTG
GCCGCCCCCCGTGCTGCAATGCTATTTAATCCACACGACTCTCGGCAACGGATATCTCGG
CTCTCGCATCGATGAAGAACGTAGCGAAATGCGATACCTGGTGTGAATTGCAGAATCCCG
CGAACCATCGAGTCTTTGAACGCAAGTTGCGCCCGAGGCCATTCGGCTGAGGGCACGCCT
GCCTGGGCGTCACGCAAAACACGCTCCCACACCCTCATCGGGTAGCGGGACGCGGCATGT
GGTCCCCCGTAGCGCAAGCTGCGGTGGGCCGAAGATATGGCTGCCGGCGTATCGTGCCGG
ACACAGCGCGTGGTGGGCGACCTCGCTTTACTTACCGCAGTGCATCCGGGGCGTAGCCGA
CGTGATGGCCTCTAAGGACCCTATGAACGGTGCGCACGACG

```
>ITS.M820_C7Frc_rev
GGAAGTAAAAGTCGTAACAAGGTTTCCGTAGGTGAACCTGCGGAAGGATCATTGTCGTGA
CCCTGACCAAAATAGACCGTGCACGCGTCATCCACCCTGCCGGCGGCGGCACCGTTCGTC
GCTCGGCCAAGTCCTCGACAACCTCCTCTCCTCGGAGTGGGGGCTCGGGGTAAAAGAACC
CACGACGCCTAAGGCGTCAAGGAACACTGTGCCTAGCCCGGGGACGCGGACGGCTTGCTG
GCCGCCCCCCGTGCTGCAATGCTATTTAATCCACACGACTCTCGGCAACGGATATCTCGG
CTCTCGCATCGATGAAGAACGTAGCGAAATGCGATACCTGGTGTGAATTGCAGAATCCCG
CGAACCATCGAGTCTTTGAACGCAAGTTGCGCCCGAGGCCATTCGGCTGAGGGCACGCCT
GCCTGGGCGTCACGCAAAACACGCTCCCACACCCTCATCGGGTAGCGGGACGCGGCATGT
GGTCCCCCGTAGCGCAAGCTGCGGTGGGCCGAAGATATGGCTGCCGGCGTATCGTGCCGG
ACACAGCGCGTGGTGGGCGACCTCGCTTTACTTACCGCAGTGCATCCGGGGCGTAGCCGA
CGTGATGGCCTCTAAGGACCCTTTGAACGGTGCGCACGACG
>ITS.M820_C9F
GGAAGTAAAAGTCGTAACAAGGTTTCCGTAGGTGAACCTGCGGAAGGATCATTGTCGTGA
CCCTGACCAAAATAGACCGTGCACGCGTCATCCAGCCTGCCGGCGGCGGCACCGTTCGTC
GCTCGGCCAAGTCCTCGACAACCTCCTCTCCTCGGAGTGGGGGCTCGGGGTAAAAGAACC
CACGACGCCTAAGGCGTCAAGGAACACTGTGCCTAGCCCGGGGACGCGGACGGCTTGCTG
GCCGCCCCCCGTGCTGCAATGCTATTTAATCCACACGACTCTCGGCAACGGATATCTCGG
CTCTCGCATCGATGAAGAACGTAGCGAAATGCGATACCTGGTGTGAATTGCAGAATCCCG
CGAACCATCGAGTCTTTGAACGCAAGTTGCGCCCGAGGCCATTCGGCTGAGGGCACGCCT
GCCTGGGCGTCACGCAAAACACGCTCCCACACCCTCATCGGGTAGCGGGACGCGGCATGT
GGTCCCCCGTAGCGCAAGCTGCGGTGGGCCGAAGATATGGCTGCCGGCGTATCGTGCCGG
ACACAGCGCGTGGTGGGCGACCTCGCTTTACTTACCGCAGTGCATCCGGGGCGTAGCCGA
CGTGATGGCCTCTAAGGACCCTATGAACGGTGCGCACGACG
>ITS.M06084_C1R
GGAAGTAAAAGTCGTAACAAGGTTTCCGTAGGTGAACCTGCGGAAGGATCATTGTCGTGA
CCCTGACCAAAATAGACCGTGCACGCGTCATCCAGCCCGCCGGCGGCGGCACCGTTCGTC
GCTCGGCCAAGTCCTCGACAACCTCCTCTCCTCGGAGTGGGGGCTCGGGGTAAAAGAACC
CACGACGCCTAAGGCGTCAAGGAACACTGTGCCTAGCCCGGGGACGCGTACGGCTTGCTG
GCCGCCCCCCGTGCTGCAATGCTATTTAATCCACACGACTCTCGGCAACGGATATCTCGG
CTCTCGCATCGATGAAGAACGTAGCGAAATGCGATACCTGGTGTGAATTGCAGAATCCCG
CGAACCATCGAGTCTTTGAACGCAAGTTGCGCCCGAGGCCATTCGGCTGAGGGCACGCCT
GCCTGGGCGTCACGCAAAACACGCTCCCACCCCCTCATCGGGTAGCGGGACGCGGCATGT
GGCCCCCCGTAGCGCAAGCTGCGGTGGGCCGAAGATATGGCTGCCGGCGTATCGTGCCGG
ACACAGCGCGTGGTGAGCGACCTCGCTTTACTTACCGCAGTGCATCCGGGGCGTAGCCGA
CGTGATGGCCTCCAAGGACCCTATGAACGGTGCGCACGACG
>ITS.M06084_C2R
GGAAGTAAAAGTCGTAACAAGGTTTCCGTAGGTGAACCTGCGGAAGGATCATTGTCGTGA
CCCTGACCAAAATAGACCGCGCACGCGTAATCCAGCCTGCCGGCGATGGCACCGTCCGTC
GCTCGGCCAAGTCCTCGACAACCTCATCTCCTCGGAGTGGGGCTCGGGGTAAAAGAACCC
ACGACGCCTAAGGCGTCAAGGAACACTGTGCCTAGCCCGGGGACGCGGACGGCTTGCTGG
CCGCCCCCCGTGCTGCAATGCTATTTAATCCACACGACTCTCGGCAACGGATATCTCGGC
TCTCGCATCGATGAAGAACGTAGCGAAATGCGATACCTGGTGTGAATTGCAGAATCCCGC
GAACCATCGAGTCTTTGAACGCAAGTTGCGCCCGAGGCCATTCGGCTGAGGGCACGCCTG
CCTGGGCGTCACGCAAAACACGCTCCCACCCCCTCATCGGGTAGCGGGACGCGGCATGTG
GCCCCCCGTAGCGCAAGCTGCGGTGGGCCGAAGATATGGCTGCCGGCGTGTCGTGCCGGA
CACAGCGCGTGGTGAGCGACCTCGCTTTACTTACCGCAGTGCATCCGGGGCGTAGCCGAC
GTGATGGCCTCCAAGGACCCTATGAACGGTGCGCACGACG
>ITS.M06084_C3R
GGAAGTAAAAGTCGTAACAAGGTTTCCGTAGGTGAACCTGCGGAAGGATCATTGTCGTGA
CCCTGACCAAAATAGACCGTGCACGCGTCATCCAGCCTGCCGGCGGCGCCACCGTTCGTC
GCTCGGCCAAGTCCTCGACAACCTCCTCTCCTCGGAGTGGGGGCTCGGGCTAAAAGAACC
CACGACGCCTAAGGCGTCAAGGAACACTGTGCCTAGCCCGGGGACGCGGACGGCTTGCTG
GCCGCCCCCCATGCTGCAATGCTATTTAATCCACACGACTCTCGGCAACGGATATCTCGG
```

CTCTCGCATCGATGAAGAACGTAGCGAAATGCGATACCTGGTGTGAATTGCAGAATCCCG
CGAACCATCGAGTCTTTGAACGCAAGTTGCGCCCGAAGCCATTCGGCTGAGGGCACGCCT
GCCTGGGCGTCACGCAAAACACGCTCGCACACCCTCATCGGGTAGCGGGACGCGGCATGT
GGTCCCCCGTAGCGCAAGCTGCGGTGGGCCGAAGATATGGCTGCCGGCGTATCGTGCCGG
ACACAGCGCATGGTGGGCGACCTCACTTTACTTACCGCAGTGCATCCGGGGCAAAGCCGA
CGTGATGGCCTCTAAGGACCCTATGAACGGTGCGCACGACG
>ITS.M06084_C4Frc_rev
GGAAGTAAAAGTCGTAACAAGGTTTCCGTAGGTGAACCTGCGGAAGGATCATTGTCGTGA
CCCTGACCAAAATAGACCGCGCACGCGTAATCCAGCCTGCCGGCGATGGCACCGTCCGTC
GCTCGGCCAAGTCCTCGACAACCTCATCTCCTCGGAGTGGGGCTCGGGGTAAAAGAACCC
ACGACGCCTAAGGCGTCAAGGAACACTGTGCCTAGCCCGGGGACGCGTACGGCTTGCTGG
CCGCCCCCCGTGCTGCAATGCTATTTAATCCACACGACTCTCGGCAACGGATATCTCGGC
TCTCGCATCGATGAAGAACGTAGCGAAATGCGATACCTGGTGTGAATTGCAGAATCCCGC
GAACCATCGAGTCTTTGAACGCAAGTTGCGCCCGAGGCCATTCGGCTGAGGGCACGCCTG
CCTGGGCGTCACGCAAAACACGCTCCCACCCCCTCATCGGGTAGCGGGACGCGGCATGTG
GCCCCCCGTAGCGCAAGCTGCGGTGGGCCGAAGATATGGTTGCCGGCGTATCGTGCCGGA
CACAGCGCGTGGTGGGCGACCTCGCTTTACTTACCGCAGTGCATCCGGGGCGTAGCCGAC
GTGATGGCCTCTAAGGACCCTATGAACGGTGCGCACGACG
>ITS.M06084_C5Frc_rev
GGAAGTAAAAGTCGTAACAAGGTTTCCGTAGGTGAACCTGCGGAAGGATCATTGTCGTGA
CCCTGACCAAAATAGACCGTGCACACGTCATCCAGCCTGCCGGCGGCGGCACCGTTCGTC
GCTCGGCCAAGTCCTCGACAACCTCCTCTCCTCGGAGTGGGGGCTCGGGGTAAAAGAACC
CACGACGCCTAAGGCGTCAAGGAACACTGTGCCTAGCCCGGGGACGCGGACGGCTTGCTG
GCCGCCCCCCGTGCTGCAATGCTATTTAATCCACACGACTCTCGGCAACGGATATCTCGG
CTCTCGCATCGATGAAGAACGTAGCGAAATGCGATACCTGGTGTGAATTGCAGAATCCCG
CGAACCATCGAGTCTTTGAACGCAAGTTGCGCCCGAGGCCATTCGGCTGAGGGCACGCCT
GCCTGGGCGTCACGCAAAACACGCTCCCACACCCTCATCGGGTAGCGGGACGCGGCATGT
GGTCCCCCGTAGCGCAAGCTGCGGTGGGCCGAAGATATGGCTGCCGGCGTATCGTGCCGG
ACACAGCGCGTGGTGGGCGACCTCGCTTTACTTACCGCAGTGCATCCGGGGCGTAGCCGA
CGTGATGGCCTCTAAGGACCCTATGAACGGTGCGCACGACG
>ITS.M06084_C7F
GGAAGTAAAAGTCGTAACAAGGTTTCCGTAGGTGAACCTGCGGAAGGATCATTGTCGTGA
CCCTGACCAAAATAGACCGTGCACGCGTCATCCAGCCTGCCGGCGGCGGCACCGTTCGTC
GCTCGGCCAAGTCCTCGACAACCTCCTCTCCTCGGAGTGGGGGCTCGGGGTAAAAGAACC
CACGACGCCTAAGGCGTCAAGGAACACTGTGCCTAGCCCGGGGACGCGGACGGCTTGCTG
GCCGCCCCCCGTGCTGCAATGCTATTTAATCCACACGACTCTCGGCAACGGATATCTCGG
CTCTCGCATCGATGAAGAACGTAGCGAAATGCGATACCTGGTGTGAATTGCAGAATCCCG
CGAACCATCGAGTCTTTGAACGCAAGTTGCGCCCGAGGCCATTCGGCTGAGGGCACGCCT
GCCTGGGCGTCACGCAAAACACGCTCCCACACCCTCATCGGGTAGCGGGACGCGGCATGT
GGTCCCCCGTAGCGCAAGCTGCGGTGGGCCGAAGATATGGCTGCCGGCGTATCGTGCCGG
ACACAGCGCGTGGTGGGCGACCTCGCTTTACTTACCGCAGTGCATCCGGGGCGTAGCCGA
CGTGATGGCCTCTAAGGACCCTATGAACGGTGCGCACGACG
>ITS.M06084_C8F
GGAAGTAAAAGTCGTAACAAGGTTTCCGTAGGTGAACCTGCGGAAGGATCATTGTCGTGA
CCCTGACCAAAATAGACCGTGCACGCGTCATCCAGCCCGCCGGCGGCGGCACCGTTCGCC
GCTCGGCCAAGTCCTCGACAACCTCCTCTCCTCGGAGTGGGGGCCCGGGGTAAAAGAACC
CACGACGCCTAAGGCGTCAAGGAACACTGTGCCTAGCTCGGGGACGCGGACGGCTTGCTG
GCCGCCCCTCGTGCTGCAATGCTATTTAATCCACACGACTCTCGGCAACGGATATCTCGG
CTCTCGCATCGATGAAGAACGTAGCGAAATGCGATACCTGGTGTGAATTGCAGAATCCCG
CGAACCATCGAGTCTTTGAACGCAAGTTGCGCCCGAGGCCATTCGGCTGAGGGCACGCCT
GCCTGGGCGTCACGCAAAACACGCTCCCACCCCCTCATCTGGTAGCGGGACGCGGCATGT
GGCCCCCCGTAGCGCAAGCTGCGGTGGGCCGAAGATATGGCTGCCGGCGTATCGTGCCGG
ACACAGCGCGTGGTGAGCGACCTCGCTTTACTTACCGCAGTGCATCCGGGGCGTAGCCGA
CGTGATGGCCTCCAAGGACCCTATGAACGGTGCGCACGACG

>ITS.M06084_C9Frc_rev
GGAAGTAAAAGTCGTAACAAGGTTTCCGTAGGTGAACCTGCGGAAGGATCATTGTCGTGA
CCCTGACCAAAATAGACCGTGCACGCGTCATCCAGCCTGCCGGCGGCGGCACCGTTCGTC
GCTCGGCCAAGTCCTCGACAACCTCCTCTCCTCGGAGTGGGGGCTCGGGGTAAAAGAACC
CACGACGCCTAAGGCGTCAAGGAACACTGTGCCTAGCCCGGGGACGCGGACGGCTTGCTG
GCCGCCCCCCGTGCTGCAATGCTATTTAATCCACACGACTCTCGGCAACGGATATCTCGG
CTCTCGCATCGATGAAGAACGTAGCGAAATGCGATACCTGGTGTGAATTGCAGAATCCCG
CGAACCATCGAGTCTTTGAACGCAAGTTGCGCCCGAGGCCATTCGGCTGAGGGCACGCCT
GCCTGGGCGTCACGCAAAACACGCTCCCACACCCTCATCGGGTAGCGGGACGCGGCATGT
GGTCCCCCGTAGCGCAAGCTGCGGTGGGCCGAAGATATGGCTGCCGGCGTATCGTGCCGG
ACACAGCGCGTGGTGGGCGACCTCGCTTTACTTACCGCAGTGCATCCGGGGCGTAGCCGA
CGTGATGGCCTCTAAGGACCCTTTGAACGGTGCGCACGACG
>ITS.M06102_C1R
GGAAGTAAAAGTCGTAACAAGGTTTCCGTAGGTGAACCTGCGGAAGGATCATTGTCGTGA
CCCTGACCAAAATAGACCGCGCACGCGTAATCCAGCCTGCCGGCGATGGCACCGTCCGTC
GCTCGGCCAAGTCCTCGACAACCTCATCTCCTCGGAGTGGGGCTCGGGGTAAAAGAACCC
ACGACGCCTAAGGCGTCAAGGAACACTGTGCCTAGCCCGGGGACGCGTACGGCTTGCTGG
CCGCCCCCCGTGCTGCAATGCTATTTAATCCACACGACTCTCGGCAACGGATATCTCGGC
TCTCGCATCGATGAAGAACGTAGCGAAATGCGATACCTGGTGTGAATTGCAGAATCCCGC
GAACCATCGAGTCTTTGAACGCAAGTTGCGCCCGAGGCCATTCGGCTGAGGGCACGCCTG
CCTGGGCGTCACGCAAAACACGCTCCCACCCCCTCATCGGGTAGCGGGACGCGGCATGTG
GCCCCCCGTAGCGCAAGCTGCGGTGGGCCGAAGATATGGCTGCCGGCGTATCGTGCCGGA
CACAGCGCGTGGTGAGCGACCTCGCTTTACTTACCGTAGTGCATCCGGGGCGTAGCCGAC
GTGATGGCCTCCAAGGACCCTTTGAACGGTGCGCACGACG
>ITS.M06102_C2R
GGAAGTAAAAGTCGTAACAAGGTTTCCGTAGGTGAACCTGCGGAAGGATCATTGTCGTGA
CCCTGACCAAAATAGACCGCGCACGCGTAATCCAGCCTGCCGGCGATGGCACCGTCCGTC
GCTCGGCCAAGTCCTCGACAACCTCATCTCCTCGGAGTGGGGCTCGGGGTAAAAGAACCC
ACGACGCCTAAGGCGTCAAGGAACACTGTGCCTAGCCCGGGGACGCGTACGGCTTGCTGG
CCGCCCCCCGTGCTGCAATGCTATTTAATCCACACGACTCTCGGCAACGGATATCTCGGC
TCTCGCATCGATGAAGAACGTAGCGAAATGCGATACCTGGTGTGAATTGCAGAATCCCGC
GAACCATCGAGTCTTTGAACGCAAGTTGCGCCCGAGGCCATTCGGCTGAGGGCACGCCTG
CCTGGGCGTCACGCAAAACACGCTCCCACCCCCTCATCGGGTAGCGGGACGCGGCATGTG
GCCCCCCGTAGCGCAAGCTGCGGTGGGCCGAAGATATGGCTGCCGGCGTATCGTGCCGGA
CACAGCGCGTGGTGAGCGACCTCGCTTTACTTACCGCAGTGCATCCGGGGCGTAGCCGAC
GTGATGGCCTCCAAGGACCCTTTGAACGGTGCGCACGACG
>ITS.M06102_C3F
GGAAGTAAAAGTCGTAACAAGGTTTCCGTAGGTGAACCTGCGGAAGGATCATTGTCGTGA
CCCTGACCAAAATAGACCGCGCACGCGTAATCCAGCCTGCCGGCGATGGCACCGTCCGTC
GCTCGGCCAAGTCCTCGACAACCTCATCTCCTCGGAGTGGGGCTCGGGGTAAAAGAACCC
ACGACGCCTAAGGCGTCAAGGAACACTGTGCCTAGCCCGGGGACGCGGACGGCTTGCTGG
CCGCCCCCCGTGCTGCAATGCTATTTAATCCACACGACTCTCGGCAACGGATATCTCGGC
TCTCGCATCGATGAAGAACGTAGCGAAATGCGATACCTGGTGTGAATTGCAGAATCCCGC
GAACCATCGAGTCTTTGAACGCAAGTTGCGCCCGAGGCCATTCGGCTGAGGGCACGCCTG
CCTGGGCGTCACGCAAAACACGCTCCCACCCCCTCATCGGGTAGCGGGACGCGGCATGTG
GCCCCCCGTAGCGCAAGCTGCGGTGGGCCGAAGATATGGCTGCCGGCGTATCGTGCCGGA
CACAGCGCGTGGTGAGCGACCTCGCTTTACTTACCGCAGTGCATCCGGGGCGTAGCCGAC
GTGATGGCCTCTAAGGACCCTATGAACGGTGCGCACGACG
>ITS.M06102_C4F
GGAAGTAAAAGTCGTAACAAGGTTTCCGTAGGTGAACCTGCGGAAGGATCATTGTCGTGA
CCCTGACCAAAATAGACCGCGCACGCGTAATCCAGCCTGCCGGCGATGGTACCGTCCGTC
GCTCGGCCAAGTCCTCGACAACCTCATCTCCTCGGAGTGGGGCTCGGGGTAAAAGAACCC
ACGACGCCTAAGGCGTCAAGGAACACTGTGCCTAGCCCGGGGACGCGGACGGCTTGCTGG
CCGCCCCCCGTGCTGCAATGCTATTTAATCCACACGACTCTCGGCAACGGATATCTCGGC

TCTCGCATCGATGAAGAACGTAGCGAAATGCGATACCTGGTGTGAATTGCAGAATCCCGC
GAACCATCGAGTCTTTGAACGCAAGTTGCGCCCGAGGCCATTCGGCTGAGGGCACGCCTG
CCTGGGCGTCACGCAAAACACGCTCCCACCCCCTCATCGGGTAGCGGGACGCGGCATGTG
GCCCCCCGTAGCGCAAGCTGCGGTGGGCCGAAGATATGGCTGCCGGCGTATCGTGCCGGA
CACAGAGCGTGGTGAGCGACCTCGCTTTACTTACCGTAGTGCATCCGGGGCGTAGCCGAC
GTGATGGCCTCTAAGGACCCTATGAACGGTGCGCACGACG
>ITS.M06102_C5Frc_rev
GGAAGTAAAAGTCGTAACAAGGTTTCCGTAGGTGAACCTGCGGAAGGATCATTGTCGTGA
CCCTGACCAAAATAGACCGCGCACGCGTAATCCAGCCTGCCGGCGACGGCACCGTCCGTC
GCTCGGCCAAGTCCTCGACAACCTCATCTCCTCGGAGTGGGGCTCGGGGTAAAAGAACCC
ACGACGCCTAAGGCGTCAAGGAACACTGTGCCTAGCCCGGGGACGCGGACGGCTTGCTGG
CCGCCCCCCGTGCTGCAATGCTATTTAATCCACACGACTCTCGGCAACGGATATCTCGGC
TCTCGCATCGATGAAGAACGTAGCGAAATGCGATACCTGGTGTGAATTGCAGAATCCCGC
GAACCATCGAGTCTTTGAACGCAAGTTGCGCCCGAGGCCATTCGGCTGAGGGCACGCCTG
CCTGGGCGTCACGCAAAACACGCTCCCACCCCCTCATCGGGTAGCGGGACGCGGCATGTG
GCCCCCCGTAGCGCAAGCTGCGGTGGGCCGAAGATATGGCTGCCGGCGTATCGTGCCGGA
CACAGCGCGTGGTGAGCGACCTCGCTTTACTTACCGCAGTGCATCCGGGGCGTAGCCGAC
GTGATGGCCTCTAAGGACCCTATGAACGGTGCGCACGACG
>ITS.M06102_C6F
GGAAGTAAAAGTCGTAACAAGGTTTCCGTAGGTGAACCTGCGGAAGGATCATTGTCGTGA
CCCTGACCAAAATAGACCGCGCACGCGTAATCCAGCCTGCCGGCGATGGCACCGTTCGTC
GCTCGGCCAAGTCCTCGACAACCTCATCTCCTCGGAGTGGGGCTCGGGGTAAAAGAACCC
ACGACGCCTAAGGCGTCAAGGAACACTGTGCCTAGCCCGGGGACGCGGACGGCTTGCTGG
CCGCCCCCGTGCTGCAATGCTATTTAATCCACACGACTCTCGGCAACGGATATCTCGGCT
CTCGCATCGATGAAGAACGTAGCGAAATGCGATACCTGGTGTGAATTGCAGAATCCCGCG
AACCATCGAGTCTTTGAACGCAAGTTGCGCCCGAGGCCATTCGGCTGAGGGCACGCCTGC
CTGGGCGTCACGCAAAACACGCTCCCACCCCCTCATCGGGTAGCGGGACGCGGCATGTGG
CCCCCCGTAGCGCAAGCTGCGGTGGGCCGAAGATATGGCTGCCGGCGTATCGTGCCGGAC
ACAGCGCGTGGTGAGCGACCTCGCTTTACTTACCGCAGTGCATCCGGGGCGTAGCCGACG
TGATGGCCTCTAAGGACCCTATGAACGGTGCGCACGACG
>ITS.M06102_C7F
GGAAGTAAAAGTCGTAACAAGGTTTCCGTAGGTGAACCTGCGGAAGGATCATTGTCGTGA
CCCTGACCAAAATAGACCGCGCACGCGTAATCCAGCCTGCCGGCGATGGCACCGTCCGTC
GCTCGGCCAAGTCCTCGACAACCTCATCTCCTCGGAGTGGGGCTCGGGGTAAAAGAACCC
ACGACGCCTAAGGCGTCAAGGAACACTGTGCCTAGCCCGGGGACGCGGACGGCTTGCTGG
CCGCTCCCCGTGCTGCAATGCTATTTAATCCACACGACTCTCGGCAACGGATATCTCGGC
TCTCGCATCGATGAAGAACGTAGCGAAATGCGATACCTGGTGTGAATTGCAGAATCCCGC
GAACCATCGAGTCTTTGAACGCAAGTTGCGCCCGAGGCCATTCGGCTGAGGGCACGCCTG
CCTGGGCGTCACGCAAAACACGCTCCCACCCCCTCATCGGGTAGCGGGACGCGGCATGTG
GCCCCCCGTAGCGCAAGCTGCGGTGGGCCGAAGATATGGCTGCCGGCGTATCGTGCCGGA
CACAGCGCGTGGTGAGCGACCTCGCTTTACTTACCGTAGTGCATCCGGGGCGTAGCCGAC
GTGATGGCCTCCAAGGACCCTATGAACGGTGCGCACGACG
>ITS.M06102_C8Frc_rev
GGAAGTAAAAGTCGTAACAAGGTTTCCGTAGGTGAACCTGCGGAAGGATCATTGTCGTGA
CCCTGACCAAAATAGACCGCGCACGCGTAATCCAGCCTGCCGGCGATGGCACCGTCCGTC
GCTCGGCCAAGTCCTCGACAACCTCATCTCCTCGGAGTGGGGCTCGGGGTAAAAGAACCC
ACGACGCCTAAGGCGTCAAGGAACACTGTGCCTAGCCCGGGGACGCGGACGGCTTGCTGG
CCGCCCCCGTGCTGCAATGCTATTTAATCCACACGACTCTCGGCAACGGATATCTCGGCT
CTCGCATCGATGAAGAACGTAGCGAAATGCGATACCTGGTGTGAATTGCAGAATCCCGCG
AACCATTGAGTCTTTGAACGCAAGTTGCGCCCGAGGCCATTCGGCTGAGGGCACGCCTGC
CTGGGCGTCACGCAAAACACGCTCCCACCCCCTCATCGGGTAGCGGGACGCGGCATGTGG
CCCCCCGTAGCGCAAGCTGCGGTGGGCCGAAGATATGGCTGCCGGCGTATCGTGCCGGAC
ACAGCGCGTGGTGAGCGACCTCGCTTTACTTACCGCAGTGCATCCGGGGCGTAGCCGACG
TGATGGCCTCCAAGGACCCTATGAACGGTGCGCACGACG
