# Supplementary material for: Pollen-mediated gene flow from transgenic perennial creeping bentgrass and hybridization at the landscape level
Source: PLoS One. 2017 Mar 3;12(3):e0173308. doi: 10.1371/journal.pone.0173308 (PMC5336273; doi:10.1371/journal.pone.0173308)
Supplement: S1 Table — Plant and seedling ID (Sample Name), presence of the cp4 epsps transgene (GR), allele for the matK indel marker (matKdel), alleles for each of the 12 Agrostis chloroplast SSR markers (Acp_), and chloroplast haplotype (cpSSR). (PDF) [file pone.0173308.s002.pdf]

Sample Name	GR	matKdel	Acp32	Acp26b	Acp30b	Acp28b	Acp31	Acp27b	Acp23	Acp18	Acp24	Acp22	Acp19	Acp12	cpSSR	Observation
090	Pos	124	86	148	218	292	173	350	144	213	234	125	195	259	CB	established plant
332	Neg	124	87	148	219	294	173	350	144	213	234	125	195	258	CB	established plant
405	Neg	124	87	148	219	292	174	350	144	213	234	126	195	258	CB	established plant
M008A	Neg	124	87	149	219	293	173	350	144	213	234	125	195	258	CB	established plant
M06084	Neg	124	87	148	219	293	174	351	144	213	234	125	?	257	CB	established plant
M193	Neg	n/a	86	148	218	292	173	350	144	?	234	125	195	259	CB	established plant
M397	Neg	124	87	149	219	293	173	350	144	213	234	125	195	258	CB	established plant
M543	Pos	124	86	148	218	292	173	350	144	213	234	125	195	259	CB	established plant
M627	Pos	124	86	148	218	292	173	350	144	213	234	125	?	259	CB	established plant
M647	Pos	124	86	148	218	292	173	350	144	213	234	125	?	259	CB	established plant
M820	Pos	124	86	148	218	292	173	350	144	213	234	125	195	259	CB	established plant
M06102	Neg	n/a	n/a	n/a	n/a	n/a	n/a	n/a	n/a	n/a	n/a	n/a	n/a	n/a	n/a	established plant
404	Neg	106	87	148	219	292	172	350	143	?	235	125	195	258	RT	established plant
483	Neg	106	87	149	218	293	174	350	145	213	234	125	195	258	RT	established plant
396_3RF	Neg	106	89	145	218	291	168	348	143	213	238	125	?	257	RF	established plant
397_1RF	Neg	106	89	145	218	291	168	348	143	213	238	125	?	257	RF	established plant
447_1RF	Neg	106	89	145	218	291	168	348	143	213	238	125	?	257	RF	established plant
447_2RF	Neg	106	89	145	218	291	168	348	143	213	238	125	?	257	RF	established plant
464_1RF	Neg	106	89	145	218	291	168	348	143	213	238	125	?	257	RF	established plant
464_2RF	Neg	106	89	145	218	291	168	348	143	213	238	125	?	257	RF	established plant
464_3RF	Neg	106	89	145	218	291	168	348	143	213	238	125	?	257	RF	established plant
M06107_2RF	Neg	106	89	145	218	291	168	348	143	213	238	125	?	257	RF	established plant
M898_1RF	Neg	106	89	145	218	291	168	348	143	213	238	125	?	257	RF	established plant
M898_2RF	Neg	106	89	145	218	291	168	348	143	213	238	125	?	257	RF	established plant
RFin268_1	Neg	106	89	145	218	291	168	348	143	213	238	125	?	257	RF	established plant
RFin447_2	Neg	106	89	145	218	291	168	348	143	213	238	125	195	257	RF	established plant
Bksp_1	Neg	124	86	148	218	292	173	350	144	213	234	125	195	259	CB	reference
Bksp_11	Neg	124	87	149	220	293	172	349	144	213	235	125	195	258	CB	reference
Bksp_12	Neg	124	87	149	220	293	172	349	144	213	235	125	195	258	CB	reference
Bksp_13	Neg	124	87	149	220	293	172	349	144	213	235	125	195	258	CB	reference
Bksp_14	Neg	124	87	149	219	293	173	350	144	213	234	125	195	258	CB	reference
PalS_4	Neg	124	87	149	219	293	173	350	144	213	234	125	195	258	CB	reference
PalS_7	Neg	124	87	148	219	292	174	350	144	213	234	126	195	258	CB	reference
PenA4_1	Neg	124	87	148	219	294	173	350	143	213	234	125	195	258	CB	reference
PenA4_2	Neg	124	87	148	219	294	173	350	144	213	234	125	195	258	CB	reference
PenA4_3	Neg	124	87	148	219	294	173	350	144	213	234	125	195	258	CB	reference
PenA4_4	Neg	124	87	148	219	294	173	350	144	213	234	125	195	258	CB	reference
PenG2_1	Neg	124	86	148	218	292	173	350	144	213	234	125	195	259	CB	reference
PenG2_2	Neg	124	86	148	218	292	173	350	144	213	234	125	195	259	CB	reference

PenG2_3	Neg	124	87	148	219	294	173	350	144	213	234	125	195	258	CB	reference
PenG2_4	Neg	124	87	148	219	294	173	350	144	213	234	125	195	258	CB	reference
PenX_11	Neg	124	86	148	218	292	173	350	144	213	234	125	195	259	CB	reference
PenX_12	Neg	124	87	148	219	294	173	350	144	213	234	125	195	258	CB	reference
PenX_13	Neg	124	86	148	218	292	173	350	144	213	234	125	195	259	CB	reference
PenX_14	Neg	124	86	148	218	292	173	350	144	213	234	125	195	259	CB	reference
PenX_17	Neg	124	86	148	218	292	173	350	144	213	234	125	195	259	CB	reference
PenX_18	Neg	124	86	148	218	292	173	350	144	213	234	125	195	259	CB	reference
PenX_2	Neg	124	86	148	218	292	173	350	144	213	234	125	195	259	CB	reference
PenX_3	Neg	124	87	148	219	294	173	350	144	213	234	125	195	258	CB	reference
Cemtry_6RT	Neg	106	87	149	218	293	174	350	145	213	234	125	195	258	RT	reference
Cemtry_7RT	Neg	106	87	149	218	293	174	350	145	213	234	125	195	258	RT	reference
Cemtry_8RT	Neg	106	88	145	218	292	193	348	142	213	233	126	195	258	RT	reference
gigS_1	Neg	106	88	145	218	292	193	348	142	213	233	126	193	258	RT	reference
gigS_2	Neg	106	87	148	219	292	172	350	143	214	235	125	195	258	RT	reference
gigS_3	Neg	106	88	145	218	292	193	348	142	213	233	126	193	258	RT	reference
gigS_4	Neg	106	87	148	219	292	172	350	143	214	235	125	195	258	RT	reference
gigS_5	Neg	n/a	88	145	218	292	193	348	142	213	?	126	?	258	RT	reference
gigS_6	Neg	106	87	149	218	293	174	350	145	213	234	125	195	258	RT	reference
gigS_7	Neg	106	87	149	218	293	174	350	145	213	234	125	195	258	RT	reference
gigS_8	Neg	106	87	149	219	292	173	350	143	213	235	125	195	258	RT	reference
gigS_8_2	Neg	106	87	149	219	292	173	350	143	213	235	125	195	258	RT	reference
Reton_1	Neg	106	87	148	219	292	172	350	143	214	235	125	195	258	RT	reference
Reton_2	Neg	106	87	148	219	292	172	350	143	214	235	125	195	258	RT	reference
Straker_1	Neg	106	88	145	218	298	193	348	142	213	233	126	193	258	RT	reference
Svj5_1	Neg	106	88	145	218	293	193	348	142	213	234	125	194	258	RT	reference
12_6_1	Pos	124	87	149	219	293	173	350	144	213	234	125	195	258	CB	seedling
12_6_2	Pos	124	86	148	218	292	173	350	144	213	234	125	195	259	CB	seedling
12_6_3	Pos	124	86	148	218	292	173	350	144	213	234	125	195	259	CB	seedling
12_6_4	Pos	124	86	148	218	292	173	350	144	213	234	125	195	258	CB	seedling
12_6_5	Pos	124	86	148	218	292	173	350	144	213	234	125	195	259	CB	seedling
12_8_2	Pos	124	87	149	219	293	173	350	144	213	235	125	195	258	CB	seedling
12_8_3	Pos	124	86	148	218	292	173	350	144	213	234	125	195	258	CB	seedling
13_3_1	Pos	124	87	149	219	293	173	350	144	213	234	125	195	258	CB	seedling
13_3_2	Pos	124	87	149	219	293	173	350	144	213	234	125	195	258	CB	seedling
13_4_1	Pos	124	87	149	219	293	173	350	144	213	234	125	195	258	CB	seedling
13_4_2	Pos	124	87	149	219	293	173	350	144	213	234	125	195	258	CB	seedling
13_4_3	Pos	124	87	149	219	293	173	350	144	213	234	125	195	258	CB	seedling
13_4_4	Pos	124	87	149	219	292	174	350	144	213	234	125	195	258	CB	seedling
13_6_1	Pos	124	87	149	219	294	174	351	144	213	234	125	195	258	CB	seedling

15_1	Pos	124	86	148	218	292	173	350	144	213	234	125	195	259	CB	seedling
16_1	Pos	124	87	149	219	293	173	350	144	213	234	125	195	258	CB	seedling
16_15	Pos	124	87	149	219	293	173	350	144	213	234	125	195	258	CB	seedling
16_22	Pos	124	87	149	219	293	173	350	144	213	234	125	195	258	CB	seedling
16_25	Pos	124	87	149	219	293	173	350	144	213	234	125	195	258	CB	seedling
16_4	Pos	124	87	149	219	293	173	350	144	213	234	125	195	258	CB	seedling
16_5	Pos	124	87	149	219	293	173	350	144	213	234	125	195	258	CB	seedling
16_6	Pos	124	87	149	219	293	173	350	144	213	234	125	195	258	CB	seedling
16_7	Pos	124	87	149	219	293	173	350	144	213	234	125	195	258	CB	seedling
16_8	Pos	124	87	149	219	293	173	350	144	213	234	125	195	258	CB	seedling
201_1	Pos	124	86	148	218	292	173	350	144	213	234	125	195	259	CB	seedling
201_2	Pos	124	86	148	218	292	173	350	144	213	234	125	?	259	CB	seedling
201_3	Pos	124	86	148	218	292	173	350	144	213	234	125	195	259	CB	seedling
201_4	Pos	124	86	148	218	292	173	350	144	213	234	125	195	259	CB	seedling
201_5	Pos	124	86	148	218	292	173	350	144	213	234	125	195	259	CB	seedling
201_8	Pos	124	86	148	218	292	173	350	144	213	234	125	195	259	CB	seedling
332_1	Pos	124	87	148	219	294	173	350	144	213	234	125	195	258	CB	seedling
405_1	Pos	124	87	148	219	292	174	350	144	213	234	126	195	258	CB	seedling
405_2	Pos	124	87	148	219	292	174	350	144	213	234	126	195	258	CB	seedling
405_3	Pos	124	87	148	219	292	174	350	144	213	234	126	195	258	CB	seedling
405_4	Pos	124	87	148	219	292	174	350	144	213	234	126	195	258	CB	seedling
74_2_1	Pos	124	87	149	219	293	173	350	144	213	234	125	195	258	CB	seedling
74_2_2	Pos	124	87	149	219	293	173	350	144	213	234	125	195	258	CB	seedling
M008_1	Pos	124	87	149	219	293	173	350	144	213	234	125	195	258	CB	seedling
M008_2	Pos	124	87	149	219	293	173	350	144	213	234	125	195	258	CB	seedling
M008_3	Pos	124	87	149	219	293	173	350	144	213	234	125	195	258	CB	seedling
M008_4	Pos	124	87	149	219	293	173	350	144	213	234	125	195	258	CB	seedling
M008_5	Pos	n/a	87	149	219	293	173	350	144	213	234	125	195	258	CB	seedling
M193_1	Pos	124	86	148	218	292	173	350	144	213	?	125	195	259	CB	seedling
M193_2	Pos	124	85	148	218	292	173	350	144	213	234	125	195	259	CB	seedling
M193_4	Pos	124	86	148	218	292	173	350	144	213	234	125	195	259	CB	seedling
M193_5	Pos	124	86	148	218	292	173	350	144	213	234	125	195	259	CB	seedling
M193_6	Pos	124	86	148	218	292	173	350	144	213	234	125	195	259	CB	seedling
M397_1	Pos	124	87	149	219	293	173	350	144	213	234	125	195	258	CB	seedling
M397_2	Pos	124	87	149	219	293	173	350	144	213	234	125	195	258	CB	seedling
M397_3	Pos	124	87	149	219	293	173	350	144	213	234	125	195	258	CB	seedling
M397_4	Pos	n/a	87	149	219	293	173	350	144	213	234	125	195	258	CB	seedling
17_10_1	Pos	106	87	149	218	293	174	350	145	213	234	125	195	258	RT	seedling
17_10_2	Pos	106	87	149	218	293	174	350	145	213	234	125	195	258	RT	seedling
17_10_3	Pos	n/a	87	149	218	293	174	350	145	213	234	125	195	258	RT	seedling

17_10_4	Pos	106	87	149	218	293	174	350	145	213	234	125	195	258	RT	seedling
17_10_5	Pos	106	87	149	218	293	174	350	145	213	234	125	195	258	RT	seedling
17_10_9	Pos	106	87	149	218	293	174	350	145	213	234	125	195	258	RT	seedling
404_1	Pos	106	87	148	219	292	172	350	143	214	235	125	195	258	RT	seedling
404_1	Pos	106	87	148	219	292	172	350	143	214	235	125	195	258	RT	seedling
48_6_1	Pos	106	88	145	218	292	193	348	142	213	?	126	195	258	RT	seedling
48_6_2	Pos	106	88	145	218	292	193	348	142	213	?	126	?	258	RT	seedling
48_6_3	Pos	106	88	145	218	292	193	348	142	213	?	126	?	258	RT	seedling
48_6_4	Pos	106	88	145	218	292	193	348	142	213	?	126	?	258	RT	seedling
483_1	Pos	106	87	149	218	293	174	350	145	213	234	125	195	258	RT	seedling
49_1_1	Pos	106	88	145	218	292	193	348	142	213	?	126	193	258	RT	seedling
49_1_2	Pos	106	88	145	218	292	193	348	142	213	?	126	?	258	RT	seedling
49_3_1	Pos	106	88	145	218	292	193	348	142	213	?	126	?	258	RT	seedling
49_6_1	Pos	106	88	145	218	292	193	348	142	213	?	126	?	258	RT	seedling
49_6_2	Pos	106	88	145	218	292	193	348	142	213	?	126	?	258	RT	seedling
49_6_2	Pos	106	88	145	218	292	193	348	142	213	?	126	?	258	RT	seedling
49_6_3	Pos	106	88	145	218	292	193	348	142	213	?	126	?	258	RT	seedling
49_7_4	Pos	106	88	145	218	292	193	348	142	213	?	126	?	258	RT	seedling
49_7_5	Pos	106	88	145	218	292	193	348	142	213	?	126	?	258	RT	seedling
49_7_7	Pos	106	88	145	218	292	193	348	142	213	?	126	?	258	RT	seedling
49_7_9	Pos	106	88	145	218	292	193	348	142	213	?	126	?	258	RT	seedling
74_1_1	Pos	106	87	149	218	293	174	350	145	213	234	125	195	258	RT	seedling
74_1_2	Pos	106	87	149	218	293	174	350	145	213	234	125	195	258	RT	seedling
83_3_1	Pos	106	87	148	219	292	172	350	143	214	235	125	195	258	RT	seedling
83_3_2	Pos	106	87	148	219	292	172	350	143	214	235	125	195	258	RT	seedling
83_3_3	Pos	106	87	148	219	292	172	350	143	214	?	125	195	258	RT	seedling
